# *FAM222A* encodes a protein which accumulates in plaques in Alzheimer’s disease

**DOI:** 10.1038/s41467-019-13962-0

**Published:** 2020-01-21

**Authors:** Tingxiang Yan, Jingjing Liang, Ju Gao, Luwen Wang, Hisashi Fujioka, The Alzheimer Disease Neuroimaging Initiative, Michael W. Weiner, Norbert Schuff, Howard J. Rosen, Bruce L. Miller, David Perry, Paul Aisen, Arthur W. Toga, Gustavo Jimenez, Michael Donohue, Devon Gessert, Kelly Harless, Jennifer Salazar, Yuliana Cabrera, Sarah Walter, Lindsey Hergesheimer, Arthur W. Toga, Karen Crawford, Scott Neu, Lon S. Schneider, Sonia Pawluczyk, Mauricio Becerra, Liberty Teodoro, Bryan M. Spann, Paul Aisen, Ronald Petersen, Clifford R. Jack, Matthew Bernstein, Bret Borowski, Jeff Gunter, Matt Senjem, Prashanthi Vemuri, David Jones, Kejal Kantarci, Chad Ward, Sara S. Mason, Colleen S. Albers, David Knopman, Kris Johnson, Neill R. Graff-Radford, Francine Parfitt, Kim Poki-Walker, William Jagust, Susan Landau, John Q. Trojanowki, Leslie M. Shaw, Jason H. Karlawish, David A. Wolk, Sanjeev Vaishnavi, Christopher M. Clark, Steven E. Arnold, Virginia Lee, Magdalena Korecka, Michal Figurski, Laurel Beckett, Danielle Harvey, Charles DeCArli, Evan Fletcher, Pauline Maillard, John Olichney, Owen Carmichael, Robert C. Green, Reisa A. Sperling, Keith A. Johnson, Gad A. Marshall, Andrew J. Saykin, Tatiana M. Foroud, Li Shen, Kelley Faber, Sungeun Kim, Kwangsik Nho, Martin R. Farlow, Ann Marie Hake, Brandy R. Matthews, Jared R. Brosch, Scott Herring, John Morris, Marc Raichle, David Holtzman, John C. Morris, Nigel J. Cairns, Erin Franklin, Lisa Taylor-Reinwald, Beau Ances, David Winkfield, Maria Carroll, Angela Oliver, Mary L. Creech, Mark A. Mintun, Stacy Schneider, Lew Kuller, Chet Mathis, Oscar L. Lopez, MaryAnn Oakley, Donna M. Simpson, Steven Paul, Norman Relkin, Gloria Chiang, Michael Lin, Lisa Ravdin, Peter Davies, M. Marcel Mesulam, Marek-Marsel Mesulam, Emily Rogalski, Kristine Lipowski, Sandra Weintraub, Borna Bonakdarpour, Diana Kerwin, Chuang-Kuo Wu, Nancy Johnson, Peter J. Snyder, Tom Montine, Michael Donohue, Lean Thal, James Brewer, Helen Vanderswag, Adam Fleisher, Paul Thompson, Ellen Woo, Daniel H. S. Silverman, Edmond Teng, Sarah Kremen, Liana Apostolova, Kathleen Tingus, Po H. Lu, George Bartzokis, Robert A. Koeppe, Jaimie Ziolkowski, Judith L. Heidebrink, Joanne L. Lord, Norm Foster, Marilyn Albert, Chiadi Onyike, Daniel D’Agostino, Stephanie Kielb, Joseph Quinn, Lisa C. Silbert, Betty Lind, Jeffrey A. Kaye, Raina Carter, Sara Dolen, Javier Villanueva-Meyer, Valory Pavlik, Nathaniel Pacini, Ashley Lamb, Joseph S. Kass, Rachelle S. Doody, Victoria Shibley, Munir Chowdhury, Susan Rountree, Mimi Dang, Yaakov Stern, Lawrence S. Honig, Karen L. Bell, Randy Yeh, Daniel Marson, David Geldmacher, Marissa Natelson, Randall Griffith, David Clark, John Brockington, Hillel Grossman, Effie Mitsis, Raj C. Shah, Melissa Lamar, Patricia Samuels, Martin Sadowski, Mohammed O. Sheikh, Jamika Singleton-Garvin, Anaztasia Ulysse, Mrunalini Gaikwad, P. Murali Doraiswamy, Olga James, Salvador Borges-Neto, Terence Z. Wong, Edward Coleman, Charles D. Smith, Greg Jicha, Peter Hardy, Riham El Khouli, Elizabeth Oates, Gary Conrad, Anton P. Porsteinsson, Kim Martin, Nancy Kowalksi, Melanie Keltz, Bonnie S. Goldstein, Kelly M. Makino, M. Saleem Ismail, Connie Brand, Gaby Thai, Aimee Pierce, Beatriz Yanez, Elizabeth Sosa, Megan Witbracht, Steven Potkin, Kyle Womack, Dana Mathews, Mary Quiceno, Allan I. Levey, James J. Lah, Janet S. Cellar, Jeffrey M. Burns, Russell H. Swerdlow, William M. Brooks, Christopher H. van Dyck, Richard E. Carson, Pradeep Varma, Howard Chertkow, Howard Bergman, Chris Hosein, Raymond Scott Turner, Kathleen Johnson, Brigid Reynolds, Neil Kowall, Ronald Killiany, Andrew E. Budson, Alexander Norbash, Patricia Lynn Johnson, Thomas O. Obisesan, Ntekim E. Oyonumo, Joanne Allard, Olu Ogunlana, Alan Lerner, Paula Ogrocki, Curtis Tatsuoka, Parianne Fatica, Sterling Johnson, Sanjay Asthana, Cynthia M. Carlsson, Jerome Yesavage, Joy L. Taylor, Steven Chao, Barton Lane, Allyson Rosen, Jared Tinklenberg, Douglas W. Scharre, Maria Kataki, Rawan Tarawneh, Earl A. Zimmerman, Dzintra Celmins, David Hart, Laura A. Flashman, Marc Seltzer, Mary L. Hynes, Robert B. Santulli, Kaycee M. Sink, Mia Yang, Akiva Mintz, Delwyn D. Miller, Karen Ekstam Smith, Hristina Koleva, Ki Won Nam, Hyungsub Shim, Susan K. Schultz, Amanda Smith, Christi Leach, Balebail Ashok Raj, Kristin Fargher, Eric M. Reiman, Kewei Chen, Pierre Tariot, Anna Burke, Joel Hetelle, Kathryn DeMarco, Nadira Trncic, Adam Fleisher, Stephanie Reeder, Edward Zamrini, Christine M. Belden, Sherye A. Sirrel, Ranjan Duara, Maria T. Greig-Custo, Rosemarie Rodriguez, Charles Bernick, Donna Munic, Zaven Khachaturian, Neil Buckholtz, John Hsiao, William Potter, Howard Fillit, Franz Hefti, Carl Sadowsky, Teresa Villena, Ging-Yuek Robin Hsiung, Benita Mudge, Vesna Sossi, Howard Feldman, Michele Assaly, Elizabeth Finger, Stephen Pasternack, William Pavlosky, Irina Rachinsky, Dick Drost, Andrew Kertesz, Sandra Black, Bojana Stefanovic, Chrinthaka Heyn, Brian R. Ott, Geoffrey Tremont, Lori A. Daniello, Courtney Bodge, Stephen Salloway, Paul Malloy, Stephen Correia, Athena Lee, Godfrey D. Pearlson, Karen Blank, Karen Anderson, Vernice Bates, Horacio Capote, Michelle Rainka, Jacobo Mintzer, Kenneth Spicer, David Bachman, Elizabeth Finger, Stephen Pasternak, Irina Rachinsky, John Rogers, Andrew Kertesz, Dick Drost, Elizabeth Finger, Stephen Pasternak, Irina Rachinsky, John Rogers, Andrew Kertesz, Dick Drost, Nunzio Pomara, Raymundo Hernando, Antero Sarrael, Smita Kittur, Michael Borrie, T.-Y. Lee, Rob Bartha, Richard Frank, Nick Fox, Veronika Logovinsky, Maria Corrillo, Greg Sorensen, Xiaofeng Zhu, Xinglong Wang

**Affiliations:** 1 Department of Pathology, Case Western Reserve University, Cleveland, OH USA; 2 Department of Population and Quantitative Health Sciences, Case Western Reserve University, Cleveland, OH USA; 3 Electron Microscopy Core Facility, Case Western Reserve University, Cleveland, OH USA; 4 University of California, San Francisco, CA USA; 5 University of Southern California, San Francisco, CA USA; 6 Mayo Clinic, Rochester, MN USA; 7 Mayo Clinic, Jacksonville, FL USA; 8 University of California, Berkeley, CA USA; 9 University of Pennsylvania, Philadelphia, PA USA; 10 University of California, Davis, CA USA; 11 Brigham and Women’s Hospital, Boston, MA USA; 12 Indiana University, Indianapolis, IN USA; 13 Washington University, St. Louis, MO USA; 14 University of Pittsburgh, Pittsburgh, PA USA; 15 Cornell University, New York, NY USA; 16 Albert Einstein College of Medicine of Yeshiva University, New York, NY USA; 17 Northwestern University, Chicago, IL USA; 18 Brown University, Providence, RI USA; 19 University of Washington, Seattle, WA USA; 20 University of California, San Diego, CA USA; 21 University of California, Los Angeles, CA USA; 22 University of Michigan, Ann Arbor, MI USA; 23 University of Utah, Salt Lake City, UT USA; 24 Johns Hopkins University, Baltimore, MD USA; 25 Oregon Health & Science University, Portland, OR USA; 26 Baylor College of Medicine, Houston, TX USA; 27 Columbia University Medical Center, New York, NY USA; 28 University of Alabama, Birmingham, AL USA; 29 Mount Sinai School of Medicine, New York, NY USA; 30 Rush University Medical Center, Chicago, IL USA; 31 New York University, New York, NY USA; 32 Duke University Medical Center, Durham, NC USA; 33 University of Kentucky, Lexington, KY USA; 34 University of Rochester Medical Center, Rochester, NY USA; 35 University of California, Irvine, CA USA; 36 University of Texas Southwestern Medical School, Dallas, TX USA; 37 Emory University, Atlanta, GA USA; 38 University of Kansas Medical Center, Kansas City, KS USA; 39 Yale University School of Medicine, New Haven, CT USA; 40 McGill University Montreal-Jewish General Hospital, Montreal, QC Canada; 41 Georgetown University Medical Center, Washington, DC USA; 42 Boston University, Boston, MA USA; 43 Howard University, Washington, DC USA; 44 University Hospitals, Cleveland, OH USA; 45 University of Wisconsin, Madison, WI USA; 46 Stanford University, Stanford, CA USA; 47 Ohio State University, Columbus, OH USA; 48 Albany Medical College, Albany, NY USA; 49 Dartmouth-Hitchcock Medical Center, Lebanon, NH USA; 50 Wake Forest University Health Sciences, Winston-Salem, NC USA; 51 University of Iowa College of Medicine, Iowa City, IA USA; 52 University of South Florida, Health Byrd Alzheimer’s Institute, Tampa, FL USA; 53 Banner Alzheimer’s Institute, Phoenix, AZ USA; 54 Banner Sun Health Research Institute, Sun City, AZ USA; 55 Wien Center, Miami Beach, FL USA; 56 Cleveland Clinic Lou Ruvo Center for Brain Health, Cleveland, OH USA; 57 Prevent Alzheimer’s Disease, Rockville, MD 2020 USA; 58 National Institute on Aging, Baltimore, MD USA; 59 National Institute of Mental Health, Rockville, MD USA; 60 AD Drug Discovery Foundation, New York, NY USA; 61 Acumen Pharmaceuticals, Livermore, CA USA; 62 Premiere Research Institute, Palm Beach Neurology, West Palm Beach, FL USA; 63 U.B.C. Clinic for AD & Related Disorders, Vancouver, BC Canada; 64 Cognitive Neurology - St. Joseph’s, London, ON Canada; 65 Sunnybrook Health Sciences, Vancouver, ON Canada; 66 Rhode Island Hospital, Providence, RI USA; 67 Butler Hospital, Butler, PA USA; 68 Hartford Hospital, Olin Neuropsychiatry Research Center, Hartford, CT USA; 69 Dent Neurologic Institute, Orchard Park, NY USA; 70 Medical University South Carolina, Charleston, SC USA; 71 St. Joseph’s Health Care, London, ON Canada; 72 Nathan Kline Institute, Orangeburg, NY USA; 73 Neurological Care of CNY, Liverpool, NY USA; 74 Parkwood Institute, London, ON USA; 75 Richard Frank Consulting, London, UK; 76 University of London, London, UK; 77 Eli Lilly and Company, Indianapolis, IN USA; 78 Alzheimer’s Association, Chicago, IL USA; 79 Siemens, Henkestr, DE Germany

**Keywords:** Genome-wide association studies, Alzheimer's disease

## Abstract

Alzheimer’s disease (AD) is characterized by amyloid plaques and progressive cerebral atrophy. Here, we report *FAM222A* as a putative brain atrophy susceptibility gene. Our cross-phenotype association analysis of imaging genetics indicates a potential link between *FAM222A* and AD-related regional brain atrophy. The protein encoded by *FAM222A* is predominantly expressed in the CNS and is increased in brains of patients with AD and in an AD mouse model. It accumulates within amyloid deposits, physically interacts with amyloid-β (Aβ) via its N-terminal Aβ binding domain, and facilitates Aβ aggregation. Intracerebroventricular infusion or forced expression of this protein exacerbates neuroinflammation and cognitive dysfunction in an AD mouse model whereas ablation of this protein suppresses the formation of amyloid deposits, neuroinflammation and cognitive deficits in the AD mouse model. Our data support the pathological relevance of protein encoded by *FAM222A* in AD.

## Abstract (web summary)

In this study the authors identify a possible link between the gene *FAM222A* and brain atrophy. The protein it encodes is found to accumulate in plaques seen in Alzheimer’s disease, and functional analysis suggests it interacts with amyloid-beta.

## Introduction

Alzheimer’s disease (AD), the leading cause of dementia named for Dr. Alois Alzheimer, is characterized by pathologic hallmarks amyloid plaques and neurofibrillary tangles, and accompanied by other prominent pathological changes such as progressive atrophy of the brain, neuropil threads, dystrophic neurites, granulovacuolar degeneration, Hirano bodies, and cerebrovascular amyloid^1^. Amyloid plaques are spherical extracellular lesions composed of amyloid-β (Aβ) peptides, whereas neurofibrillary tangles are intracellular lesions made up of hyperphosphorylated form of the microtubule-associated protein tau. Although many risk factors such as aging, lifestyle, and environmental factors are usually considered for the pathogenesis, AD is increasingly proposed to be a genetically dichotomous disease in the early-onset familial form showing classical Mendelian inheritance with little influence from the environment (EOAD), or in the late-onset sporadic form inherited in a non-Mendelian fashion (LOAD)^2^.

Less than 10% of AD cases are EOAD with only a small fraction caused by autosomal dominantly inherited genetic changes in amyloid precursor protein (APP), presenilin 1 (PS1) or presenilin 2 (PS2), all of which are responsible for the overproduction of Aβ and the earlier formation of amyloid plaques^3^. Though more than 90% of AD cases are LOAD referred to as sporadic AD without family history, they have the similar clinical and pathologic phenotypes as EOAD and are heritable^4^. In the past decade, intensive efforts have been made to identify over 25 genes associated with AD^5^. In support of the dominant amyloid cascade hypothesis suggesting Aβ deposition in the brain as the primary cause, a number of AD-associated genes are enriched in the APP processing pathway, and involved in Aβ overproduction and amyloid plaque deposition though their encoded proteins are usually not directly associated with amyloid plaques.

Quantitative structural magnetic resonance imaging (MRI) has been extensively used for assessment of AD-related structural differences in selective brain regions^6^. Genome-wide association studies (GWAS) using MRI measures have identified several AD risk variants^7,8^. Likewise, the MRI changes in statistically-defined regions of interest (ROI) were found closely associated with reported AD risk variants^9,10^. Genetic loci harboring variants can be associated with multiple, sometimes seemingly distinct, traits^11,12^. The test for such associations, i.e., cross-phenotype association test, has been increasingly employed to investigate the genetic overlap between multiple traits and diseases. Our previous study has developed a cross-phenotype association analysis (CPASSOC) that can integrate association evidence from GWAS summary statistics of multiple traits, either correlated, independent, continuous, or binary traits, and has been successfully used to identify four loci associated with hypertension-related traits missed by a single-trait analysis^13^. In this study, we performed CPASSOC analysis of MRI measures and genetic datasets, and identified a possible link between *FAM222A* and AD-related regional brain atrophy. To understand its pathological role in AD, we investigated the protein encoded by *FAM222A* in patients with AD or transgenic mice for AD, and found its characteristic accumulation within the center of amyloid deposits. Further mechanistic study revealed that this protein could physically interact with Aβ and regulate Aβ aggregation and amyloid formation. Our results therefore identify a protein that likely plays an important role in amyloidosis, a finding providing perspective for AD pathogenesis.

## Results

### Susceptibility of regional brain atrophy to FAM222A in AD

**Fig. 1:**
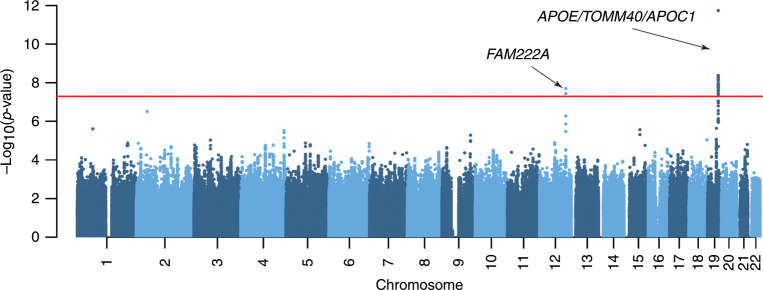
CPASSOC analysis of the ADNI cohort. Manhattan plot of CPASSOC analysis combining GWAS summary statistics of ROIs in the green-colored module (Supplementary Fig. 1m) including left hippocampus, right hippocampus, basal forebrain, entorhinal area, and planum polare. The red line represents the threshold of *P* = 5 × 10^−8^ for the genome-wide significance level. Arrows indicate loci associated with regional brain atrophy. Source data are provided as a Source Data file (Source Data for GWAS in Fig. 1).

To identify brain atrophy-related imaging quantitative trait loci, we employed a genome-wide whole brain approach to analyze the imaging genetic dataset from the Alzheimer’s Disease Neuroimaging Initiative (ADNI) (Supplementary Fig. 1a). After GWAS and the estimation of shared genetic contributions among 145 ROIs spanning the entire brain by linkage disequilibrium (LD) regression method^14^ (Supplementary Fig. 1b, c), we attempted to extract disease-related ROIs and detect genetic variants associated with them. With hierarchical clustering analysis on a genetic correlation network (Supplementary Fig. 1d–k), 16 modules of ROIs with high within-module genetic correlation were generated (Supplementary Fig. 1l, m). We further combined GWAS summary statistics of ROIs in each module using CPASSOC we developed^13^. Previously reported AD top markers, *APOE* single nucleotide polymorphism (SNP) rs429358^15^, *TOMM40* SNP rs2075650^15^, *APOC1* SNP rs12721051^16^, and rs117028417 on *FAM222A* were found in one module (Fig. 1, Supplementary Figs. 2, 3 and Supplementary Table 1), which consists of five ROIs including left hippocampus, right hippocampus, basal forebrain, entorhinal area, and planum polare, brain areas we know are affected by AD^17–19^ and well predict AD (Supplementary Fig. 4). SNP rs117028417 had a minor allele A (frequency = 0.044) with positive effects for all 5 ROIs in the ADNI cohort (*P* = 1.95 × 10^−8^ for CPASSOC analysis; Supplementary Table 2), and was further validated to be associated with the mean volume of hippocampus, one of the earliest affected brain regions in AD, in the Enhancing NeuroImaging Genetics through Meta-Analysis (ENIGMA) consortium cohort comprising MRI images of 30,717 individuals from 50 cohorts^20^ with the same effect direction (*β* = 38.1, *P* = 8.29 × 10^−3^; Supplementary Table 2).

On a genome-wide scale, *FAM222A* rs117028417 was only marginally associated with AD diagnosis in the International Genomics of Alzheimer’s Project (I-GAP, *P* = 0.052)^21^. The positron emission tomography (PET) imaging using radiotracer ^18^F-Florbetapir (AV45) provides quantitative measures of amyloid pathology in vivo^22^. To investigate whether SNP rs117028417 is associated with brain Aβ accumulation, we performed single SNP association test of rs117028417 with AV45 standard uptake value ratio (SUVR) measures for brain regions available in the PET scan dataset from ADNI. The minor allele A of rs117028417 was found significantly associated with longitudinal decrease but not baseline of AV45 SUVR measures in anterior or posterior cingulate (*P* < 0.0125) (Supplementary Table 3), which is relatively consistent with the findings from brain volume associations, where the association direction was positive (i.e. higher volume). rs117028417 is located in an intergenic region that is approximately 5 kb downstream of *FAM222A* and 8 kb downstream of *TRPV4*. We searched among published GWAS studies of AD or AD-related biomarkers and did not identify common variants previously reported on these two genes. We thus further performed gene-based burden and SKAT tests^23,24^ of coding variants on both *FAM222A* and *TRPV4*. All of the coding variants on these two genes available in ADNI whole-genome sequencing data were low-frequency or rare variants with minor allele frequency less than 0.05 (Supplementary Table 4). *FAM222A*, but not *TRPV4*, displayed significant association with AV45 SUVR longitudinal changes in anterior or posterior cingulate or lateral parietal regions in the burden test after adjusting for 10 independent tests (*P* < 0.005) (Supplementary Table 5), collectively suggesting a possible role of *FAM222A* in brain amyloid deposition. However, when we tested rs117028417 for genetic association with AD cerebrospinal fluid (CSF) Aβ and tau biomarkers, only nominal association of rs117028417 with total tau annual change could be discovered, and there was no association with baseline CSF Aβ and tau on both single SNP association tests and variant burden tests (Supplementary Tables 6, 7), indicating that *FAM222A* variant may not have a strong genetic influence on AD biomarkers.

### *FAM222A*-encoded protein accumulates within amyloid plaques

**Fig. 2:**
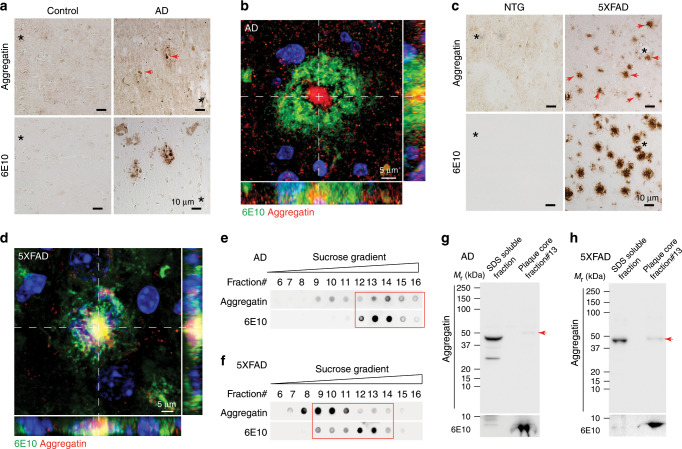
Aggregatin accumulates within the center of amyloid deposits. **a** Representative images of immunohistochemistry of Aggregatin (arrowheads) and amyloid plaques (stained by the 6E10 antibody) in adjacent sections (denoted by asterisks) of cortices of sporadic AD patients. **b** Representative fluorescent images of Aggregatin (Red), amyloid plaques (Green, stained by the 6E10 antibody) and DAPI nuclei staining (Blue) in cortices of sporadic AD. **c** Representative images of immunohistochemistry of Aggregatin (arrowheads) and amyloid plaques (stained by the 6E10 antibody) in adjacent sections (denoted by asterisks) of brains of 6-month-old 5XFAD mice. **d** Representative images of Aggregatin (Red), amyloid plaques (Green, stained by the 6E10 antibody) and DAPI nuclei staining (Blue) in brains of 6-month-old 5XFAD mice. **e**, **f** Representative dot blots of Aggregatin and Aβ (6E10) in serial fractions of amyloid plaques separated by differential centrifugation in sucrose gradient from sporadic AD patients (**e**) or 6-month-old 5XFAD mice (**f**). **g**, **h** Representative immunoblots of Aggregatin and Aβ (6E10) in the SDS-resistant insoluble core-enriched fraction from sporadic AD patients (**g**) or 6-month-old 5XFAD mice (**h**). Arrow heads point Aggregatin. Due to the presence of urea used for plaque core protein extraction, plaque core fractions show slight shifts compared to SDS soluble fraction. All experiments were independently performed at least three times. Source data are provided as a Source Data file (Source Data for Statistics and Blots).

The large independent AD brain imaging dataset including GWAS studies is not available at this time, making it difficult to further validate the genetic link between *FAM222A* and AD-related brain atrophy. However, to elucidate the possible pathological role of *FAM222A* in AD, we carried out experimental validation to focus on its encoded protein, which we designated as Aggregatin. Aggregatin consists of 452 amino acids with a predicted molecular weight of 47 kD, and has not yet been characterized. Using a well-characterized specific antibody against Aggregatin (Supplementary Fig. 5a–e), Aggregatin was found predominantly expressed in the central nervous system (CNS) including both the brain and the spinal cord, but not in other tissues such as heart, spleen, lung, kidney, or liver in mice or humans (Supplementary Fig. 5d, e). There was a slight increase in the expression of Aggregatin in brain lysates from AD patients compared to age-matched control subjects (Supplementary Fig. 5f–h). The most distinct pattern of Aggregatin immunostaining observed in AD was that Aggregatin was remarkably immunoreactive within the center of amyloid plaques, which were stained by the pan-Aβ antibodies 6E10 and 4G8, the N-terminal truncated and modified pyroglutamate Aβ species Aβ[N3pe] antibody 82E1, fibrillar Aβ dye thioflavin-S (Thio-S) or oligomer Aβ antibody NU-4^25^ (Fig. 2a, b and Supplementary Fig. 6a–e). In contrast, all control brain sections lacking detectable amyloid plaques demonstrated weak diffusive Aggregatin immunoreactivity without association with puncta (Fig. 2a).

Robust Aggregatin staining of the central core of amyloid deposits was consistently observed in the brains of multiple mouse models for AD including 5XFAD^26^, TgCRND8^27^, APP/PS1^28^, Tg2576^29^, and 3xTg^30^ transgenic mice overexpressing human mutant APP along with or without human mutant PS1 (Fig. 2c, d and Supplementary Fig. 6f–h). With the exception of 5XFAD or Tg2576 mice in which Aggregatin-positive foci were connected with wispy fibrils, Aggregatin within amyloid deposits of other transgenic mice showed negligible projecting fibrillar structures, similar as in human plaques. Despite the general localization of Aggregatin large puncta to the core of amyloid deposits, they highly co-localized with Aβ in 5XFAD mice but not in AD patients or TgCRND8 mice, together indicating that the processes contributing to amyloid deposition may be different in human and different animal models. Notably, the formation of Aggregatin puncta occurred concurrently with amyloid deposition, but was not present in the pre-depositing young 5XFAD mice (Supplementary Fig. 6i). The characteristic Aggregatin-positive core staining was abolished by the pre-absorption of primary antibodies with human recombinant Aggregatin protein (rAggregatin) purified by combined 10 K dialysis and size-exclusion chromatography, but not Aβ_1-42_ peptides (Supplementary Fig. 7a), further validating the specificity of the anti-Aggregatin antibody. To confirm the presence of Aggregatin within amyloid deposits, we isolated amyloid cores purified by sucrose density gradient fractionation of 2% sodium dodecyl sulfate (SDS) homogenized AD or 5XFAD mouse brains. Dot blot and immunoblot studies of proteins under native and denatured forms respectively confirmed the existence of full-length Aggregatin without noticeable cleaved products in the SDS-resistant insoluble core-enriched fractions positive for 6E10 (Fig. 2e–h).

### Aggregatin physically interacts with Aβ

**Fig. 3:**
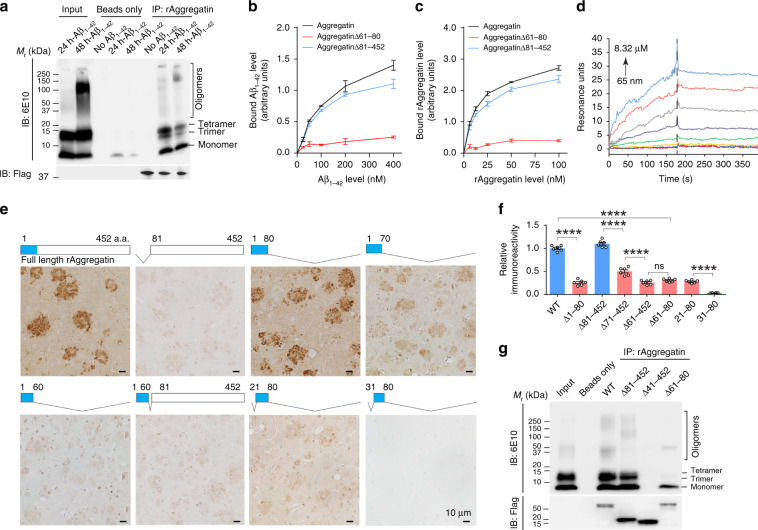
Aggregatin interacts with Aβ. **a** Coimmunoprecipitation of purified Flag-tagged rAggregatin and Aβ_1–42_ (pre-aggregated in vitro for 24 or 48 h). rAggregatin was immunoprecipitated using streptavidin magnetic beads and immunoblotted using the antibody to Flag. **b** Measurement of Aβ_1–42_ levels bound to immobilized rAggregatin (normalized to maximal rAggregatin and Aβ_1–42_ binding). *n* = 3 independent experiments. **c** Measurement of rAggregatin levels bound to immobilized Aβ_1–42_ (normalized to maximal rAggregatin and Aβ_1–42_ binding). *n* = 3 independent experiments. **d** Bio-layer interferometry measurement of the binding kinetics of monomeric Aβ_1–42_ to immobilized rAggregatin. Curves are corresponded to Aβ_1–42_ at 8320, 4160, 2080,1040, 520, 260, 130 and 65 nM from the top to bottom. **e**, **f** Representative immunohistochemistry (**e**) and quantification (**f**) of rAggregatin immunoreactivity (Flag antibody) in 5XFAD mouse brain sections after incubation with 100 nM indicated rAggregatin deletion mutants (*n* = 6 independent experiments in each group). Blocks with blue color on the top of each immunohistochemistry image show NABD. **g** Coimmunoprecipitation analysis of purified Flag-tagged rAggregatin deletion mutations and Aβ_1–42_ using streptavidin magnetic beads. Source data are provided as a Source Data file (Source Data for Statistics and Blots). Data are means ± s.e.m (± is the plus–minus sign). One-way analysis of variance (ANOVA) followed by Tukey’s multiple comparison test. ****P < 0.0001. ns, non-significant.

The radioimmunoprecipitation assay buffer (RIPA) widely used for co-immunoprecipitation failed to extract Aggregatin from AD brains (Supplementary Fig. 7b), making it difficult to examine the likely association between Aggregatin and Aβ in AD. To overcome this obstacle, we performed in vitro pull-down assays using synthetic Aβ_1–40_ or Aβ_1–42_ and rAggregatin. Dynamic light scatting (DLS), circular dichroism (CD), and SDS-PAGE assays of rAggregatin indicated that rAggregatin existed in the soluble partially folded monomeric state (Supplementary Fig. 7c–e). Notably, rAggregatin co-precipitated with different forms of Aβ_1–40_ or Aβ_1–42_ (Fig. 3a and Supplementary Fig. 7f, g). Consistently, immobilized monomeric Aβ_1–40_ or Aβ_1–42_ was also able to pull down rAggregatin (Supplementary Fig. 7h). Further surface binding affinity assays revealed that immobilized Aβ_1–40_ or Aβ_1–42_ bound to rAggregatin, and similarly, immobilized rAggregatin bound to Aβ_1–40_ or Aβ_1–42_ all within the nanomolar ranges (Fig. 3b, c and Supplementary Fig. 7i, j). In agreement with these results, surface plasmon resonance (SPR) measurements confirmed that Aβ_1–42_ bound to immobilized rAggregatin at the low nanomolar dissociation equilibrium constant (Kd) (Fig. 3d). Although no measurement was noted in blank or BSA-immobilized sensor chips (Supplementary Fig. 7k), signal spikes produced in the SPR assays may be in proportion to the mass of Aβ aggregates, making dynamic measurements unlikely consistent with the surface binding affinity assessments at the steady state. To investigate the binding of rAggregatin to Aβ ex vivo, we performed an in situ binding assay in which fixed brain sections of AD patients or 5XFAD mice were incubated with Flag-tagged rAggregatin and stained by an anti-Flag antibody. Remarkably, all amyloid deposits were labelled by rAggregatin (Supplementary Fig. 7l–n). Considering the widespread presence of Aβ in brains, it was not surprising that brain sections also showed background staining after rAggregatin incubation. Notably, amyloid deposits and the background binding of rAggregatin were completely abolished by pre-incubation of rAggregatin with Aβ_1–40_ or Aβ_1–42_ (Supplementary Fig. 7l–n), confirming that rAggregatin binds amyloid deposits by interacting with Aβ. Collectively, these results highlight the pathological relevance of Aggregatin in AD, and show that Aggregatin is a Aβ binding protein with high-affinity.

### Aggregatin binds to Aβ via its N-terminal region

Next, we generated a series of rAggregatin deletion mutants to map the binding region for Aβ. Although rAggregatin alone does not form oligomers or aggregates, the composition of Aβ preparations at the micromolar range quickly changes over time due to the formation of higher order oligomers, which are expected to influence the Aggregatin and Aβ interaction. To quantitatively identify the binding strength of different rAggregatin deletion mutants, the in situ binding assay rather than pull-down assay was used for the binding motif mapping. The deletion of residues from 1 to 80 (designated as NABD, N-terminal Aβ binding domain), but not residues outside of this region, was found to greatly reduce the binding of rAggregatin to amyloid deposits (Fig. 3e–g and Supplementary Fig. 8a, b). Recombinant NABD (rNABD) alone was able to bind to amyloid deposits or Aβ_1–42_ similar as full-length rAggregatin, and caused a dose-dependent decrease in the association between rAggregatin and amyl deposits when co-incubated (Fig. 3b, c, e–g and Supplementary Fig. 8c, d), together suggesting NABD as the domain both necessary and sufficient for Aβ binding. The residues from 61 to 80 appear to be a core motif for NABD though they alone were not sufficient to bind amyloid deposits (Fig. 3e–g and Supplementary Fig. 8a, b). Notably, rNABD bound amyloid deposits in a length-dependent manner, and rAggregatin with partial deletions of every 5 amino acids within the core motif of NABD exhibited weaker but still strong interactions with amyloid deposits (Supplementary Fig. 8a, b), further indicating that NABD may contain multiple sites cooperatively involved in Aβ binding.

### Aggregatin cross-seeds Aβ **via** direct binding

**Fig. 4:**
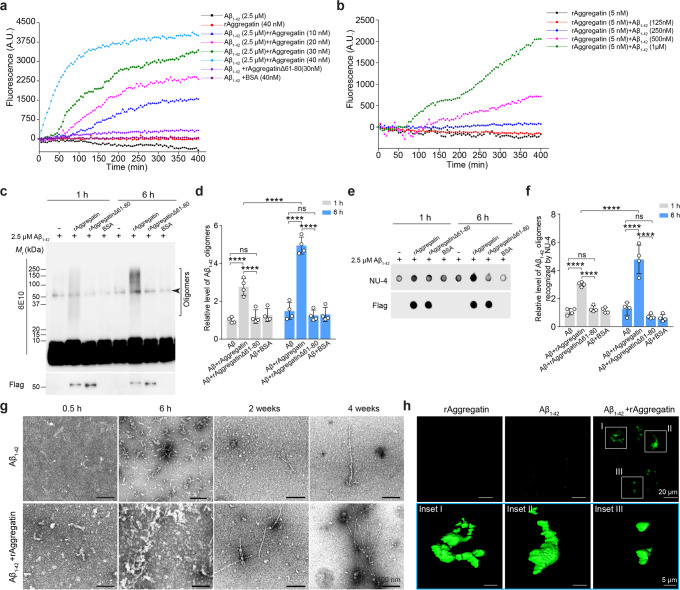
Aggregatin accelerates Aβ aggregation in vitro. **a** ThT-based assay measuring aggregation kinetics of 2.5 µM Aβ_1–42_ in the presence of various concentrations of rAggregatin (*n* = 5 independent experiments in each time points). **b** ThT-based assay measuring aggregation kinetics of various concentrations of Aβ_1–42_ in the presence of 5 nM rAggregatin (*n* = 5 independent experiments in each time points). **c**, **d** Representative immunoblot (**c**, light exposure shown in Fig. S9d) and quantification (d) of Aβ_1–42_ oligomers recognized by 6E10 in the 30 nM rAggregatin and 2.5 µM Aβ_1–42_ mixture collected after 6-h co-incubation (*n* = 4 independent experiments). Arrow head points to non-specific bands due to long exposure. **e**, **f** Representative dot blot (**e**) and quantification (**f**) of Aβ_1–42_ oligomers recognized by the oligomer Aβ specific antibody NU-4 in the 30 nM rAggregatin and 2.5 µM Aβ_1–42_ mixture collected after 6-hour co-incubation (*n* = 4 independent experiments). **g** Negative staining electron microscopy of 2.5 µM Aβ_1–42_ aggregates after 0.5-h, 6-h, 2-week, and 4-week co-incubation with or without 30 nM rAggregatin. **h** Representative 3D images of 2.5 µM Aβ_1–42_ aggregates stained by Thio-S after 4-week co-incubation with or without 30 nM rAggregatin. Source data are provided as a Source Data file (Source Data for Statistics and Blots). Data are means ± s.e.m. One-way analysis of variance (ANOVA) followed by Tukey’s multiple comparison test. ****P < 0.0001. ns, non-significant.

Given the strong interaction between Aggregatin and Aβ, we further set out to determine whether Aggregatin would influence the Aβ aggregation process. Aβ aggregation kinetics were first monitored in vitro using Aβ_1–40_ or Aβ_1–42_ for the thioflavin T (ThT) based fluorescence assay. As illustrated by changes in ThT-associated fluorescence, Aβ self-aggregated only at high concentrations whereas rAggregatin alone did not produce any observable aggregate (Fig. 4a and Supplementary Fig. 9a). Remarkably, once co-incubated with rAggregatin, Aβ was able to form aggregates at low concentrations even in the nanomolar range (Fig. 4a, b and Supplementary Fig. 9a–c). With increasing concentrations of rAggregatin, the lag times of the aggregation reaction were greatly decreased (Fig. 4a and Supplementary Fig. 9b). As a control, rAggregatinΔ61–80 had similar folding as wild type rAggregatin, but failed to induce Aβ_1–42_ aggregation (Fig. 4a and Supplementary Fig. 7d, e). These observations were confirmed using immunoblot and dot blot analyses for Aβ aggregation measurements under denatured and native conditions, which showed that Aggregatin but not rAggregatinΔ61–80 indeed promoted Aβ_1–42_ oligomerization (Fig. 4c–f and Supplementary Fig. 9d). Of note, due to the sensitivity of immunoblot, Aβ oligomer was only detectable with long exposure when Aβ_1–42_ at the low micromolar but not nanomolar was applied. Consistently, transmission electron microscopy analyses revealed that soluble Aβ_1–42_ protofibrils^31^ were more abundant and have more complicated structures in the presence of rAggregatin during the early phase of incubation when Aβ fibrils were absent (Fig. 4g). As expected, the low concentration of Aβ_1–42_ only yielded very few short and un-branched fibrils after long periods of incubation under negative staining (Fig. 4g), and rAggregatin alone did not form identifiable particles or large aggregates (Supplementary Fig. 9e). Strikingly, co-incubation of low micromolar Aβ_1–42_ with rAggregatin lead to the formation of large micrometer-long branched fibrils (Fig. 4g and Supplementary Fig. 9f), which were Thio-S-positive and visible under the fluorescent microscopy (Fig. 4h). Taken together, these data imply Aggregatin as a potent seeding factor for Aβ oligomerization and aggregation.

### Aggregatin regulates amyloid deposition

**Fig. 5:**
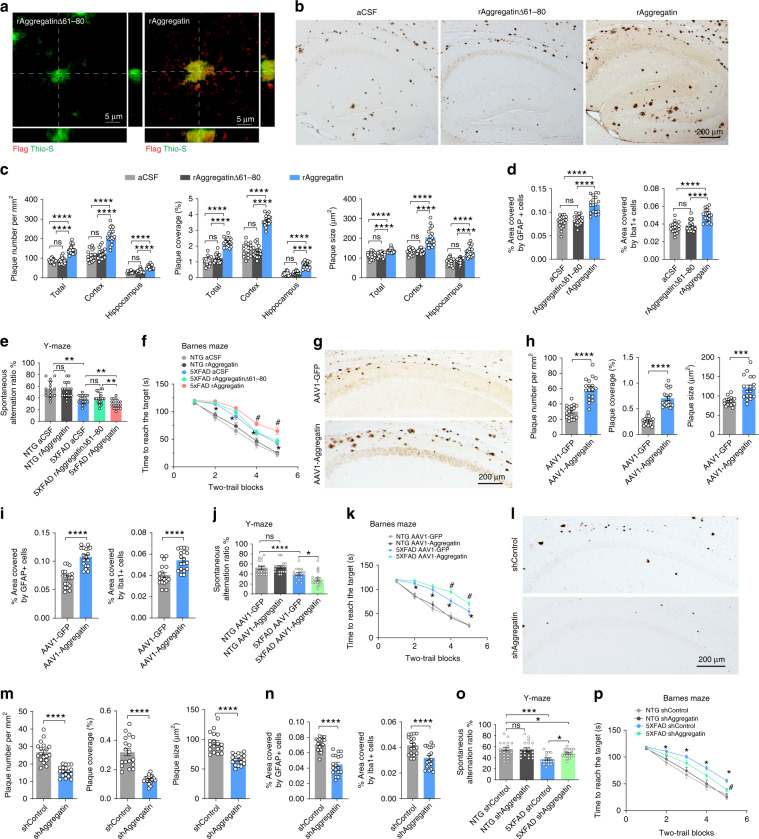
Aggregatin regulates amyloid deposits. 5-month-old 5xFAD mice were ICV infused with Flag-tagged rAggregatinΔ61–80 or rAggregatin for 4 weeks. **a** Representative images of Flag-tagged Aggregatin (Red) and amyloid plaques (Green, Thio-S) in the brain. **b**, **c** Representative images (**b**) and quantification (**c**) of plaque by NU-4 antibody in the total brain (Total), cortex or hippocampus (*n* = 18 mice in each group). **d** Quantification of astrogliosis and microgliosis in hippocampus (representative images shown in Supplementary Fig. 10f). **e**, **f** Y-maze (**e**) and Barnes maze (**f**) performance (*n* = 15, 17, 18, 18, and 18 mice for NTG aCSF, NTG rAggregatin, 5XFAD aCSF, 5XFAD rAggregatinΔ61–80, and 5XFAD rAggregatin respectively). 5-month-old 5XFAD mice were injected with AAV1-GFP or AAV1-Aggregatin at 1.5 month-old. **g**, **h** Representative images (**g**) and quantification (**h**) of plaques stained by NU-4 in the hippocampus (*n* = 18 mice in each group). **i** Quantification of astrogliosis and microgliosis in the hippocampus (*n* = 18 mice in each group). **j**, **k** Y-maze (**j**) and Barnes maze (**k**) performance (*n* = 18 mice in each group). Representative images are shown in Supplementary Fig. 11g. 5-month-old 5XFAD mice were injected with AAV1-shControl or AAV1-shAggregatin at 1.5-month-old. **l**, **m** Representative images (**h**) and quantification (**i**) of plaques stained by NU-4 in the hippocampus (*n* = 18 mice in each group). **n** Quantification of astrogliosis and microgliosis in the hippocampus (*n* = 18 mice in each group). Representative images are shown in Supplementary Fig. 12f. **o**, **p** Y-maze (**o**) and Barnes maze (**p**) performance (*n* = 18 mice in each group). Source data are provided as a Source Data file (Source Data for Statistics and Blots). Data are means ± s.e.m. Student’s *t*-test or one and two-way analysis of variance (ANOVA) followed by Tukey’s multiple comparison test. *P < 0.05, ^#^*P* < 0.05 (relative to aCSF, AAV1-GFP or shControl AAV1), ***P < 0.001, ****P < 0.0001. ns, non-significant.

Aβ levels are low in young especially predepositing 5XFAD mice^26^. To examine the effect of extracellular Aggregatin on amyloid deposition with unrestricted access to predeposit-state Aβ, we performed intracerebroventricular (ICV) infusion of Flag-tagged rAggregatin or rAggregatinΔ61–80 into 5XFAD mice at 4-month-old, when Aβ rises to high levels^26^ (Supplementary Fig. 10a). Infusion did not cause the death of mice or histological abnormalities in the brain. Importantly, the levels of total Aβ, APP or BACE1 remained unchanged 4 weeks after rAggregatin infusion, indicating that rAggregatin did not affect Aβ production or degradation (Supplementary Fig. 10b, c). ICV infused rAggregatin was detected in amyloid deposit (Fig. 5a). Remarkably, compared to age-matched control mice infused with artificial cerebrospinal fluid (aCSF), rAggregatin-infused mice showed greatly increased amyloid deposition spreading the brain at 5 months of age, which could be completely blocked by the deletion of NABD core motif (Fig. 5b, c and Supplementary Fig. 10d, e). As prominent AD pathological features, microgliosis and astrogliosis are closely associated with amyloid deposits in 5XFAD mice^26,32^. Corresponding to increased plaque load, 5XFAD mice infused with rAggregatin but not rAggregatinΔ61–80 exhibited more microgliosis and astrogliosis compared to aCSF-infused control 5XFAD mice (Fig. 5d and Supplementary Fig. 10f). 5XFAD mice begin to show cognitive deficits at around 4-months-old^33,34^. Compared with NTG mice, FAD mice exhibited significantly impaired Y-maze and Barnes-maze performance, both of which were significantly exacerbated in transgenic mice with rAggregatin but not rAggregatinΔ61–80 infusion (Fig. 5e, f). To further examine the role of neuronal Aggregatin in amyloid deposition, we injected adeno-associated virus serotype 1 encoding human Aggregatin or GFP alone under the neuron specific promoter *eSYN* (AAV1-Aggregatin or AAV1-GFP) into the hippocampus CA1 of young predepositing 5XFAD mice at 1.5-month-old (Supplementary Fig. 11a). When analyzed at 5 months of age, in line with ICV infusion experiments, intrahippocampal injection of AAV1-Aggregatin significantly increased amyloid deposition without any effect on total Aβ levels in the GFP-positive hippocampal region, but not in the brain areas without AAV1-Aggregatin delivery (Fig. 5g, h and Supplementary Fig. 11b–f), together suggesting that Aggregatin is sufficient to enhance amyloid deposition in vivo. Consistently, amyloid deposition associated microgliosis, astrogliosis, and cognitive deficits were also worsened by neuronal Aggregatin overexpression (Fig. 5i–k and Supplementary Fig. 11g). To investigate whether Aggregatin was required for amyloid deposition, we performed intrahippocampal injection of AAV1 co-expressing GFP and a short hairpin RNA targeting Aggregatin (AAV1-shAggregatin) or control shRNAi (AAV1-shControl) in predepositing 5XFAD mice (Supplementary Fig. 5b, c). It was observed that decreasing Aggregatin was not associated with neuronal loss or altered total Aβ (Supplementary Fig. 12a). At 5 months of age, the injection of AAV1-shAggregatin significantly alleviated amyloid deposition in the GFP-positive areas of hippocampus compared to AAV1-shControl injection, but not in the GFP-negative brain areas (Fig. 5l, m and Supplementary Fig. 12b–e). Likewise, Aggregatin reduction significantly alleviated amyloid deposit associated microgliosis, astrogliosis, and cognitive impairment (Fig. 5n–p and Supplementary Fig. 12f). Taken together, these results further imply that Aggregatin is also an important factor necessary for amyloid deposition.

## Discussion

Here, we report on Aggregatin, the protein encoded by *FAM222A*, as a plaque core protein directly binding Aβ and facilitating Aβ aggregation, a process thought to be central in AD onset. Therefore, this work provides strong experimental evidence supporting a pathophysiological role for Aggregatin in AD.

In people diagnosed with AD or mild cognitive impairment (MCI), a proportion of whom can progress to AD, *FAM222A* is associated with the module enriched for atrophy in AD-affected brain regions. *FAM222A* association with hippocampal volume could be validated in the replication ENIGMA cohort, together pointing to a potential mechanism by which *FAM222A* may affect regional brain atrophy. Notably, our cross phenotype association analysis also led to the identification of long-established AD risk genes *APOE*, *TOMM40*, and *APOC1* exclusively in the same module, suggesting possible genetic interplays between *FAM222A* and AD risking genes. Interestingly, although we only discovered marginal association between rs117028417 and AD diagnosis, *FAM222A*, but not the nearby gene *TRPB4*, was found significantly associated with longitudinal increase of brain amyloid deposition. Along this line, as AD is a genetically complex and multifactorial disease with different etiological subtypes, *FAM222A* variants or pathogenic mutations strongly associated with AD may be present in subsets of AD patients. Nevertheless, although our genetic discovery study did not observe a strong influence of *FAM222* variant on AD risk and biomarkers, the module enriched for *FAM222A* and previously reported AD risk variants likely represents a statistical AD-specific cluster worthy of further investigation using independent AD neuroimaging databases.

Due to the limited sample size, only a slight, but not significant increase of Aggregatin mRNA level was observed in AD cortices collected in our laboratory (Supplementary Fig. 13a, b). However, analysis of previously published microarray data of the Mount Sinai Brain Bank (MSBB) cohort^35^ found significantly increased levels of Aggregatin mRNA in many brain regions especially cortices of AD, which were also tested to be associated with AD neuropathologies (Supplementary Fig. 13c, d and Supplementary Tables 8, 9). DNA methylation is one of several epigenetic mechanisms regulating gene expression^36^ and has been implicated in AD^37^. Interestingly, several methylation sites on *FAM222A* associated with AD could be identified (Supplementary Fig. 14 and Supplementary Table 10), indicating the likely involvement of *FAM222A* in AD pathogenesis through epigenetic regulation of its gene expression. Considering Aggregatin mRNA levels were usually measured at one time-point, future studies may be interesting to investigate longitudinal changes of Aggregatin gene expression and their relationship with neuropathologies during the progression of disease. While the relationship between *FAM222A* DNA methylation, transcription, translation, and posttranslational modification remains to be determined, these interesting findings provide further genetic evidence supporting the association of *FAM222A* to AD.

Consistent with the genetic association of *FAM222A* with longitudinal brain Aβ deposition, pathologically accumulated Aggregatin, the protein encoded by *FAM222A*, is readily noted in plaques in AD and amyloid deposits in multiple APP transgenic mice, strongly illustrating the pathological function of Aggregatin. Of note, there are remarkable differences in the morphology of Aggregatin puncta and their co-localization with Aβ. Similarly, as plaques in AD patients are more complex structures than amyloid deposits in APP transgenic mice^38^, it could be expected that Aggregatin is also present differentially in amyloid core-enriched fractions from AD patients and 5XFAD mice. A number of explanations may account for the discrepancy regarding the pattern of Aggregatin puncta or presence of Aggregatin in plaques, including but not limited to differences in disease stages, the effects of Aβ clearance and degradation pathways or the length of time spent for plaque deposition. This notion is indeed supported by the observation that while only one or several condensed Aggregatin foci were present in single plaque in AD, amyloid deposits in cortex from patients with Down’s syndrome (DS), a complex genetic abnormality developing AD-like pathology, were largely associated with multiple foci (Supplementary Fig. 15).

It is still unclear how Aggregatin becomes accumulated within the center of plaques without the ability for self-aggregation. Aggregatin appears to bind Aβ_1–40_ and Aβ_1–42_ with different affinities. Along this line, amyloid plaques are made up of different N or C-terminally truncated and modified Aβ species^39^. Interestingly, we found that Aggregatin was present in exosomes (Supplementary Fig. 16). Although Aggregatin has no signal sequence and is not predicted to be secreted, this data supports the possibility that Aggregatin can be exported into the interstitial fluid. Of note, the presence of exogenously expressed Aggregatin in exosomes of cultured cells is physiologic. There may be other mechanisms responsible for Aggregation secretion under pathological conditions. As Aggregatin protein levels were upregulated in AD, there may be a complex interplay among Aβ specific forms, Aggregatin expression, post-translational modification, extracellular secretion, and other unknown factors responsible for this. Nevertheless, on the basis of the facts that Aggregatin puncta appear concurrently with amyloid plaques and does not exist in the predepositing mice, Aggregatin should accumulate in plaques before or concurrent with rather than after the well formation of plaques. Aggregatin did not form intraneuronal accumulation in AD patients and 5XFAD mice. Not surprisingly, we did not observe the presence of Aggregatin puncta in neurons bearing neurofibrillary tangles (Supplementary Fig. 17a). Along this line, intraneuronal APP and/or Aβ immunoreactivity assessed by 6E10 was not changed by Aggregatin in 5XFAD mice. Therefore, Aggregatin may not be involved in intraneuronal protein aggregation. Noteworthily, Aggregatin does not physically interact with tau and other previously reported plaque-associated proteins such as α-synuclein and APOE (Supplementary Fig. 17b–d), further implicating the likely specific link between Aggregatin and Aβ. However, as AD is a multifactorial disease, further detailed investigation will still be needed to determine the spatiotemporal relationship between Aggregatin and other AD-related pathologies especially considering the presence of Aggregatin immunoreactivity outside of plaques.

Aggregatin facilitates Aβ aggregation in vitro although it is not clear whether Aggregatin influences the primary or secondary nucleation. Increasing Aggregatin enhances, whereas reduced Aggregatin suppresses amyloid deposition and associated neuroinflammation and cognitive deficits. Of note, in addition to exacerbate Aβ pathology in adult 5XFAD mice, Aggregatin infusion causes further amyloid deposition in aged 5XFAD mice when amyloid deposit size and number largely plateau (Supplementary Fig. 18). Therefore, Aggregatin is likely an unrecognized co- or even limiting factor both necessary and sufficient for Aβ aggregating into the fibrils to form plaques. Although the bioinformatics analysis of Aggregatin amino acid sequence reveals that Aggregatin does not contain any known conserved functional motifs, our CD characterization of Aggregatin indicated it as at least a partially folded protein containing α-helix, β-sheet, and intrinsically disordered element(s) (Supplementary Fig. 7e). While the structure and physiological function of Aggregatin is still under investigation, we found that Aggregatin was exclusively expressed in the CNS. The substantial loss of Aggregatin in hippocampus does not cause neuronal death, suggesting that Aggregatin may not be vital for neuronal survival. Future studies may be feasible to generate mice with global or neuronal specific deletion of Aggregatin to test whether the knockout of Aggregatin is sufficient to completely abolish amyloid deposition and further validate the pathological role of Aggregatin in amyloid plaque formation and disease progression.

The genetic inhibition of Aggregatin-Aβ interaction was able to suppress Aggregatin-induced Aβ aggregation or amyloid deposits, suggesting that Aggregatin should directly interact with Aβ to regulate its pathology. Of note, although rNABD (i.e. Aggregatin 1–80 or Aggregatin Δ81–452) alone is able to bind Aβ, it does not induce Aβ_1–42_ aggregation or promote amyloid deposits (Supplementary Fig. 19), suggesting that the C-terminal fragment is also required for Aggregatin-induced Aβ aggregation and plaque formation. The exact mechanism for Aggregatin-mediated Aβ aggregation is still under investigation. Noteworthily, likely due to the high Aβ binding affinity of Aggregatin, the specific anti-Aggregatin antibody used in this study does not dissociate the Aggregatin-Aβ interaction or prevent Aggregatin-induced aggregation of Aβ, and likewise, ICV infusion of the specific anti-Aggregatin antibody failed to alleviate Aβ pathologies in 5XFAD mice. Thus, the Aggregatin targeted immunotherapy for AD may require the generation of high-affinity monoclonal antibodies. The genetic manipulation or infusion of Aggregatin did not change APP or Aβ levels, suggesting that Aggregatin is unlikely involved in Aβ production or degradation. However, even though Aggregatin large puncta do not overlap with neurons, microglia or astrocytes, our results cannot rule out the possibility that Aggregatin may regulate amyloidosis indirectly through neuronal function or microglial or astrocytic Aggregatin, which is also worthy of further clarification. APOE4 is the strongest genetic risk factor for LOAD. Although the involvement of APOE in Aβ metabolism might complicate the interpretation of data^40^, future studies will also be interesting to investigate Aggregatin-mediated amyloidosis in vivo on the human ApoE knock-in or ApoE knockout background as previously reported^41^.

In conclusion, we have reported *FAM222A* as a likely gene associated with AD-related regional brain atrophy, which encodes an amyloid plaque core protein pathologically involved in Aβ assembly and amyloid deposition. Our findings therefore not only inform future genetic studies of *FAM222A*, but also encourage detailed pathophysiological investigation of its encoded Aggregatin for AD and related dementia.

## Methods

### Samples, genotyping, and imputation

Data used in the preparation of this article were obtained from the ADNI database (http://www.loni.ucla.edu/ADNI). The Illumina SNP genotyping data, demographic information, APOE genotype and baseline diagnosis information from 754 ADNI-1 participants, including 213 cognitive normal individual controls, 175 AD patients, and 366 patients with mild cognitive impairment (MCI) were downloaded from ADNI database. All participants provided written informed consent and study protocols were approved by participating sites’ Institutional Review Board.

SNP genotyping of 620,901 markers on ADNI-1 participants were generated using Illumina BeadStudio 3.2 software from bead intensity data. All SNP genotypes are publicly available for download at the ADNI website. For genotype imputation analysis, only SNPs fulfilling the following criteria were included (1) per-SNP call rate ≥ 0.98; (2) minor allele frequency (MAF) ≥ 0.01; (3) *P*-value for Hardy-Weinberg equilibrium (HWE) ≥ 10^–6^ in our sample set. Imputation was performed using the software MACH-ADMIX^42^ using the 1000 Genomes Project Phase 3 V.5 (http://www.internationalgenome.org) as a reference panel. We excluded SNPs with R^2^ < 0.3, MAF < 0.01 and all INDELs from the imputed genotype data to obtain genotypes for 7,512,167 SNPs for subsequent association analyses.

### MRI analysis and extraction of imaging phenotypes

Dr. Christos Davatzikos’ group from University of Pennsylvanian analyzed the baseline MRI T1 scans of ADNI1 participants and generated the 145 ROIs spanning the entire brain by using the Multi-atlas region Segmentation (MUSE) framework^43^. In this framework, multiple atlases with semi-automatically extracted ground-truth ROI labels were first warped individually to the target image using non-linear registration methods^44,45^. To fuse the ensemble into a final segmentation, they adopted a spatial adaptive weighted voting strategy, in which a local similarity term was used for ranking and weighting ground truth labels from different atlases and an image intensity based term was used for modulating the segmentations at the boundaries of the ROIs according to the intensity profile of the subject image^43^. In validation experiments, the multi-atlas approach was showed to achieve significantly higher accuracy in comparison to single-atlas based segmentation^43^. In this study, we downloaded the volume measures of ROIs from ADNI.

### ROI-wise genome-wide association analysis in ADNI1

Autosomal chromosome SNP associations for volumes from 145 ROIs spanning whole brain were assessed by linear regression under the assumption of an additive genetic model. All models were adjusted for age, gender, education, handedness and 3 principal components to control population stratification. The genomic control for 145 GWASs ranged between 0.98 to1.02.

### Genetic correlation network analysis of brain ROIs in ADNI1

In multivariate quantitative genetics, a genetic correlation (***r***_***g***_) is the proportion of variance that two traits share due to additive genetic effects, which estimates the degree of pleiotropy or causal overlap^12,14,46^. The cross phenotype association analysis (CPASSOC) is a method proposed to integrate association evidence of multiple traits from multiple GWAS and detect cross-phenotype associations^13^. Thus, CPASSOC analysis of genetic correlated AD-related brain imaging traits could improve power to identify genetic variants associated with multiple AD-imaging traits. To identify groups of highly genetic correlated ROIs, we used the estimated pairwise ROI genetic correlations to define the brain genetic correlation network. In this network, nodes are brain ROIs while edges are estimated genetic correlations between ROIs. To extract modules from this network, we adopted a weighted gene co-expression network analysis (WGCNA) framework and used the method of topological overlap matrix (TOM) elements in hierarchical clustering to identify modular structures^47^. A flowchart for constructing a ROI genetic correlation network, extracting network modules and identifying genetic variants associated with modules using CPASSOC is presented in Supplementary Fig. 1a.

Pairwise ROI genetic correlations were estimated by the technique of cross-trait LD score regression method^14^ using the GWAS summary statistics of ROIs. For 10,400 pairs among 145 ROIs, genetic correlations were not correctly estimated for 3,255 pairs because the estimated values were either “NA”, above 1 or below −1, which might be driven by the small sample size, and these pairs were then filtered out. However, this filter may reduce power to identify variants associated with ROIs. The pairwise genetic correlations are presented in Supplementary Fig. 1 and we observed high genetic correlations among the ROIs.

We used the ROI genetic correlation matrix and power adjacency function^47^ to generate network adjacent matrix:

1 $$a_{ij} = \left| {r_{gij}} \right|^\beta$$

while *r*_*gij*_ is the genetic correlation between nodes ROI *i* and ROI *j*, and *a*_*ij*_is the connection strength between two nodes.

To choose the parameter *β* and genetic correlation *P*-value threshold, we used the scale-free network model to construct an image network. The scale-free network assumes that most nodes in a network are sparsely connected with the exception of a few hub nodes that are densely connected with other nodes^48^. In the scale-free network models, more connections are likely to occur for those hub nodes with already-high connectivity, which meet biological criteria^47,48^. We used the power law $p(k) \sim k^{ - \gamma }$to estimate the scale-free property, where *k* is the connectivity for each node and equals the number of its direct connections to other node. To generate the network, we assessed different power adjacency function parameter *β* = 2, 4, 6 and 8 and filtered the genetic correlation with different genetic correlation (*r*_*g*_) *P*-value thresholds of 0.5, 0.3, 0.2 and 0.1. For each *P*-value threshold, if the estimated genetic correlation *P*-value was larger than that, we set the genetic correlation to be 0. Using the four thresholds, we generated different networks for β = 2, 4, 6 and 8 and accessed their corresponding scale-free topology using linear regression model fitting index *R*^2^between log_10_(*p*(*k*)) and log_10_(*k*) for all nodes. We observed that a *P*-value threshold of 0.2 with *β* = 6 corresponded a network with the scale-free topology and had the largest *R*^2^of 0.61. The histogram of connectivity *k* and scale-free topology plots for networks with *β* = 6 and different P-value threshold were showed in Supplementary Fig. 2. Thus, we used the network adjacent matrix generated under this criterion for further analysis. In this network, 40 out of 145 ROIs had *k* equal to 0 and 105 ROIs were carried out in module identification analysis.

We adopted the methods introduced by WGCNA framework^47^ to identify network modules. The adjacent matrix was transformed into a topological overlap matrix (TOM) with element defined as

2 $$w_{ij} = \frac{{l_{ij} + a_{ij}}}{{\min \left\{ {k_i,k_j} \right\} + 1 - a_{ij}}}$$

where $l_{ij} = \mathop {\sum}\nolimits_u {a_{ij}a_{uj}}$ and $k_i = \mathop {\sum}\nolimits_u {a_{iu}}$is the node connectivity.

TOM based dissimilarity measure was generated by

3 $$d_{ij}^w = 1 - w_{ij}$$

This dissimilarity matrix was used as the input for average linkage hierarchical clustering. The hierarchical clustering grouped the closet ROIs and formed the branches to identify module. For the genetic correlation network, we identified 16 modules spanning the whole brain with the largest module containing 17 ROIs and the smallest containing 3 ROIs (Supplementary Fig. 2).

### CPASSOC analysis within modules

We applied the CPASSOC package developed by Zhu et al.^13^ to combine association evidence of ROIs within each module. CPASSOC can integrate association evidence from summary statistics of multiple traits and improves power when variant is associated with at least one trait. CPASSOC provides two statistics, S_Hom_ and *S*_Het_. *S*_Hom_ is similar to the fixed effect meta-analysis method^49^ but accounting for the correlation of summary statistics among cohorts induced by potential overlapped or related samples. In brief, assuming we have summary statistical results of GWAS from *J* cohorts with *K* phenotypic traits. In each cohort, single SNP-trait association was analyzed for each trait separately. Let *T*_*jk*_ be a summary statistic for a SNP, *j*^*th*^ cohort and *k*^*th*^ trait. Let ${{{\mathbf{T}}}} = (T_{11}, \cdots ,T_{J1}, \cdots ,T_{1K}, \cdots ,T_{JK})^T$ represents a vector of test statistics for testing the association of a SNP with *K* traits. Let ${{{\mathbf{\beta }}}} = \left( {\beta _{11,...,}\beta _{J1,...,}\beta _{1K,...,}\beta _{JK}} \right)^T$be the effect sizes of the SNP. The null hypothesis is H_0_: **β**=**0** and the alternative hypothesis H_1_ is that at least one of the elements of **β** is not equal to zero. We used a Wald test statistic$T_{jk} = \widehat \beta _{jk}/\widehat s_{jk}$, where $\hat \beta _{jk}$ and $\hat s_{jk}$ are the estimated coefficient and corresponding standard error for the *k*^*th*^ trait in the *j*^*th*^ cohort. It is reasonable to assume that **T** follows a multivariate normal distribution with mean **0** and correlation matrix *R* under the null hypothesis. When the effect is homogeneous, the most powerful test statistic *S*_*Hom*_ is defined as

4 $$S_{{{{\mathrm{Hom}}}}} = \frac{{e^T({{{\mathrm{RW}}}})^{ - 1}T(e^T({{{\mathrm{RW}}}})^{ - 1}T)^T}}{{e^T({{{\mathrm{WRW}}}})^{ - 1}e}}$$

which follows a *χ*^2^ distribution with one degree of freedom, where ${{{\mathbf{e}}}}^{{{\mathbf{T}}}} = (1,...,1)$ has length *J* *×* *K* and *W* is a diagonal matrix of weights for the individual test statistics. We used the sample sizes for the weights, $w_{jk} = \sqrt {n_j}$, *n*_*j*_ is sample size of the *j*^*th*^ cohort.

To further allow for different effect directions of a variant for different traits in different cohorts, we define *S*_*Het*_. We first let

5 $$S(\tau ) = \frac{{e^T\left( {R(\tau )W(\tau )} \right)^{ - 1}T(\tau )\left( {R(\tau )W(\tau )} \right)^{ - 1}\left. {T(\tau )} \right)^T}}{{e^TW(\tau )^{ - 1}R(\tau )^{ - 1}W(\tau )^{ - 1}e}}$$

Where **T**(**τ**) is the sub-vector of **T** satisfying $\left| {T_{jk}} \right| > \tau$ for a given $\tau > 0$, $R\left( \tau \right)$is a sub-matrix of *R* representing the correlation matrix, and $W\left( \tau \right)$be the diagonal submatrix of *W*, corresponding to ${{{\mathbf{T}}}}\left( {{{\mathbf{\tau }}}} \right)$. The test statistic is then

6 $$S{}_{Het} = \mathop{\max}\limits_{\tau > 0}S(\tau )$$

The asymptotic distribution of *S*_Het_ does not follow a standard distribution but can be evaluated using simulation. *S*_Het_ is an extension of *S*_Hom_ but power can be improved when the genetic effect sizes vary for different traits. The distribution of *S*_Het_ under the null hypothesis can be obtained through simulations or approximated by an estimated beta distribution.

We applied both *S*_Hom_ and *S*_Het_ to combine summary statistics for ROIs within each module. The CPASSOC analysis of multiple genetic correlated traits in identified module would allow us to identify variants that are likely to be missed by conventional GWAS of single trait and reduce the multiple comparison burden in the genetic analysis of hundreds of neuroimaging traits. Finally, we identified 15 loci with CPASSOC test *P*-value less than 1 × 10^–7^ in nine modules (Supplementary Table 1). Importantly, three previously reported AD-associated SNPs, rs429358, rs2075650 and rs439401 and the *FAM222A* SNP rs117028417 were exclusively found in one module, which were green colored in Supplementary Fig. 1M and Supplementary Table 1. The Manhattan plots and Q-Q plots of CPASSOC analysis and single ROI GWAS for this module were showed in Fig. 1 and Supplementary Fig. 2.

### Genetic analysis of AV-45 PET imaging

^18^F-Florbetapir (AV-45) PET imaging was performed at baseline and 2-year follow-up for participants enrolled in the ADNI GO and two phases^22^. UC Berkeley extracted weighted AV-45 standardized uptake value ratio (SUVR) means for four main cortical regions: frontal, anterior, and posterior cingulate, lateral parietal and lateral temporal regions (version 2019.4.12) for ADNI-GO2 participants. They also calculated composite SUVR for cortical which is weighted SUVR mean in frontal, cingulate, parietal and temporal regions. These data can be downloaded from the ADNI database. We used the SUVR mean of composite region including whole cerebellum, pons/brainstem and eroded white matter as reference. Mean AV-45 SUVR of frontal, cingulate, lateral parietal, lateral temporal and composite cortical relative to the reference were calculated. The annual percent change in SUVR means at 2-year follow-up compared to baseline was used as the main quantitative phenotype for genetic analysis. The annual percent changes in AV-45 SUVR for all five brain regions were approximately normally distributed (Supplementary Fig. 20). We collected 369 individuals with both SUVR measures for baseline and 2-year follow-up and whole-genome sequencing data. The samples included 120 healthy people, 26 people with AD, 64 people with late mild cognitive impairment (LMCI) and 159 people with early mild cognitive impairment (EMCI) diagnosed at baseline. The samples characteristics and demographics for samples are shown in Supplementary Table 11.

WGS data from 817 ADNI participants were downloaded from the ADNI dataset. WGS was performed using blood-derived genomic DNA samples and sequenced on the Illumina HiSeq2000 using paired-end read chemistry and read lengths of 100 bp at 30–40X coverage^50^. As previously described using Broad GATK and BWA-mem, reads were mapped and aligned to the human genome (build 37), then variants were called^50,51^.

For single SNP association test, association test of SNP rs117028417 with phenotypes were performed using linear regression under an additive genetic model in PLINK. Baseline age and gender were included as covariates. For gene-based association test, we extracted 8 and 6 functional coding variants defined as missense, in frame deletion/insertion, stop gained/lost, start gained/lost, splice acceptor/donor, or initiator/start codon for *FAM222A* and *TRPV4* respectively. All of those variants are rare with minor allele frequency (MAF) < 0.01 in ADNI samples. Gene-based association tests were performed using burden and SKAT^52^, adjusting age and sex as covariates.

### Genetic analysis of CSF Aβ and Tau

Collection and processing of ADNI CSF samples was described in the ADNI procedures manual (http://www.adni-info.org/). We downloaded UPENNBIOMKs dataset.csv file from ADNI website. We collected 617 individuals with both CSF Aβ_42_, tTau and pTau at baseline level and WGS data. For baseline data, since raw CSF biomarkers were skewed or bimodal skewed distributed, rank normal transformations were conducted for each biomarker separately (Supplementary Fig. 21a–f). To conduct CSF biomarkers longitudinal change genetic association, we collected 274 individuals with both baseline and 24-month follow-up CSF biomarkers and WGS data. The CSF biomarkers raw data at baseline and 2-year follow-up in 218 individuals were used to calculate annual changes in Aβ_42_, tTau and pTau separately. The annual changes of three CSF biomarkers were approximately normally distributed (Supplementary Fig. 21g–i). The samples characteristics and demographics for CSF biomarker traits association analysis are shown in Supplementary Tables 12, 13.

Association test of SNP rs117028417 with phenotypes were performed using linear regression under an additive genetic model in PLINK. Baseline age and sex were included as covariates. We extracted 8 and 15 coding variants defined as missense, in frame deletion/insertion, stop gained/lost, start gained/lost, splice acceptor/donor, or initiator/start codon for *FAM222A* and *TRPV4* respectively (Supplementary Table 4). All of those variants are rare with minor allele frequency (MAF) < 0.01 in ADNI samples. Gene-based association tests were performed using burden and SKAT^52^, adjusting age and sex as covariates.

### Analysis of FAM222A mRNA in AD

The development of the Mount Sinai Brain Bank (MSBB) cohort was described in the previous studies^35,53^. MSBB is a large AD cohort and now holds over 2,040 well-characterized human brains^53^. The datasets we used assessed a total of 125 human brains which was assembled after applying stringent inclusion/exclusion criteria and represents the full spectrum of cognitive and neuropathological disease severity^35^. Detailed sample demographic information and description of the cognitive and neuropathological traits can be seen in previously published paper by Dr. Bin Zhang’s lab^35^. We downloaded the normalized microarray data of MSBB Array Tissue Panel Study from the Synapse at http://www.synapse.org/#!Synapse:syn3157699. The RNA samples from 19 brain regions isolated from 125 MSBB specimens were collected and profiled on the Affymetrix 133AB and Affymetrix 133Plus2 platforms. RNA quality was assessed using a combination of a 260/280 ratio derived from resolution electrophoresis system (LabChipTM, Agilent Technologies, Palo Alto, CA, USA) and 3′–5′ hybridization ratios for GAPDH probes^35^. Not all brain regions for all subjects were available for analysis. There was an approximately 60 samples (40 AD, 20 controls) per brain region available for analysis. The array probes were annotated according to the Ensemble version 72 (genome build GRCh37) using the R/Biomart library. The raw microarray data were quantile normalized with all probe sets on the arrays using RMA^54^ method implemented in the R/Bioconductor package affy (v1.44) with the default parameters. The data were then corrected for covariates including sex, postmortem interval (PMI), pH and race using a linear regression model. The *FAM222A* gene expression data was identified by probe set *226487_at*. The processed FAM222A mRNA level means for groups of AD and control were compared using two-sided Welch t-test using R.

### Association analysis of *FAM222A* DNA methylation

We downloaded two datasets, E-GEOD-45775 and E-GEOD-76105, with DNA methylation profiling from the European Bioinformatics Institute (EMBL-EBI) ArrayExpress website https://www.ebi.ac.uk/arrayexpress/. Samples of dataset E-GEOD-45775 included 5 controls, 5 AD Braak stage I-II and 5 AD Braak stage V-VI (Supplementary Table 14). The methylation values were adjusted and normalized using BeadStudio software v3.2 to obtain normalized beta and average Beta detect P-value. The array used the HumanMethylation27_270596_v.1.2 design and one methylation site cg01335367 was identified located on chr12:109734355–109734404 (*GRCh38.p12*), associated with *FAM222A*. We analyzed the association between methylation in cg01335367 with AD using logistic regression and adjusted for sex. We also performed one-way analysis of variance (ANOVA) to determine differences between methylation levels of control and different Alzheimer Braak stage groups. Study E-GEOD-70615 investigated DNA methylation profiling in the superior temporal gyrus (STG). Samples included 34 AD and 34 non-demented controls, which had 52 European, 8 Hispanic, 6 African, 1 Asian Americans and 1 unknown (Supplementary Table 15). The Beta values from the probes were quantile normalized using lumi package in R. We performed association analysis in 52 European Americans only. The association between methylation in those sites with AD were analyzed using logistic regression model adjusting age, gender and estimated cellular proportions (neuronal vs. glial).

### Mice and human tissues

Mouse surgery and procedures were performed according to the NIH guidelines and were approved by the Institutional Animal Care and Use Committee (IACUC) at Case Western Reserve University. 5xFAD transgenic mice (B6.Cg-Tg(APPSwFlLon, PSEN1*M146L*L286V) 6799Vas/Mmjax, JAX#008730) were purchased from the Jackson Laboratory. The use of all human tissue samples was approved by the University Hospitals Institutional Review Board (IRB) for human investigation at University Hospitals Case Medical Center at Cleveland. Human brain tissues obtained postmortemly from University Hospitals of Cleveland were fixed, and 6-μm-thick consecutive sections were prepared. The information of fixed or frozen human tissues were listed in Supplementary Tables 16, 17.

### Immunocytochemistry, immunofluorescence and immunoblot

Immunocytochemistry was performed by the peroxidase anti-peroxidase protocol. Taken briefly, paraffin embedded brain tissue sections were first deparaffinized in xylene and rehydration in graded ethanol and incubated in Tris Buffered Saline (TBS, 50 mM Tris-HCl and 150 mM NaCl, pH 7.6) for 10 min before antigen retrieval in 1X Immuno/DNA retriever with citrate (BioSB, Santa Barbara, CA) under pressure using BioSB’s TintoRetriever pressure cooker. Sections were rinsed with distilled H_2_O, and blocked with 10% normal goat serum (NGS) in TBS at room temperature (RT) for 30 min. Tissue sections were further incubated with primary antibodies in TBS containing 1% NGS overnight at 4 °C, and immunostained by the peroxidase-antiperoxidase based method. For double Immunofluorescence staining, paraffin embedded tissue sections were deparaffinized in xylene and re-hydrated in graded ethanol without H_2_O_2_ incubation as described above. The sections were incubated in phosphate buffered saline (PBS) at RT for 10 min followed by block with 10% NGS in PBS for 45 min at RT. The sections were incubated with primary antibodies in PBS containing 1% NGS overnight at 4 °C. After being washed with 1% NGS in PBS for 10 min, the sections were incubated in 10% NGS for 10 min and followed by three quick washes with 1% NGS in PBS. Then, the sections were incubated with Alexa Fluor 488 or 568 dye labeled secondary antibodies (1:300, Invitrogen, Carlsbad, CA) for 2 h at RT in dark, washed three times with PBS, stained with DAPI, washed again with PBS for three times, and finally mounted with Fluoromount-G mounting medium (Southern Biotech, Birmingham, AL). For thioflavin-S staining, slides were incubated with 1% thioflavin-S (Santa Cruz Biotechnology, Dallas, TX) for 8 min, washed 2 times with 80% ethanol, and 1 time with 95% ethanol and PBS, then stained with DAPI. For immunoblot, human or mice tissue samples were all lysed with TBS plus 1 mM phenylmethylsulfonyl fluoride (PMSF) (Millipore, Burlington, MA), protease inhibitor cocktail (Sigma Aldrich, St. Louis, MO) and phosphatase inhibitor cocktail (Sigma Aldrich, St. Louis, MO). Equal amounts of total protein extract were resolved by SDS-PAGE and transferred to Immobilon-P (Millipore, Burlington, MA). Following blocking with 10% nonfat dry milk, primary and secondary antibodies were applied and the blots developed with Immobilon Western Chemiluminescent HRP Substrate (Millipore, Burlington, MA). Images were taken by ChemiDoc Touch Imager (Bio-rad, Hercules, CA). Primary antibodies used in this study are listed in Supplementary Table 18. The dilution of antibodies used for IF or IHC. 4G8 (BioLegend, SIG-39220; IF, 1:1000), 6E10 (BioLegend, 803001; IF and IHC, 1:1000), 82E1 (IBL, 10323; IF, 1:1000), Aggregatin (Abcam, ab122626; IF/IHC, 1:100), Aggregatin (LifeSpan BioSciences, LS-C170630; IHC, 1:1000), Aggregatin (Aviva Systems Biology, ARP69038_P050; IHC, 1:1000), Flag (Sigma Aldrich, F1804; IF/IHC, 1:1000), Flag (Thermo Fisher, PA1–984B; IHC, 1:200), Flag (Cell Signaling Technology, 2368; IHC, 1:200), Flag-HRP (Proteintech, HRP-66008; IHC, 1:1000), GFP (Abcam, ab32146; IHC, 1:500), Myc (Thermo Fisher, MA1–21316; IHC, 1:1000), Myc (Cell Signaling Technology, 2276; IHC, 1:500), and Nu4 (Klein lab, IF/IHC, 1:2000). All uncropped and unprocessed blots are provided in the Source Data file (Source Data for Statistics and Blots).

### Expression vectors and recombinant proteins

pcDNA3.1(+) (Invitrogen, Carlsbad, CA) plasmid was modified to express recombinant proteins to express recombinant proteins containing a 4xFlag-Twin-Strep-tag at their N-terminal. The cDNA of full length or truncated human Aggregatin were inserted into the modified pcDNA3.1(+) plasmid. All primers and cDNA constructs used in this study are listed in Supplementary Data 1 and Supplementary Table 19. Eight micrograms plasmid was used to transfect one 10 cm dish of Lenti-293T cells with TransIT^®^−293 Transfection Reagent (Mirus, Madison, WI). Cells were collected at 24 h after transfection and lysed by lysis buffer (100 mM Tris-HCl, 150 mM NaCl, 1 mM EDTA and 1% NP40, pH 8.0) containing 1 mM PMSF (Millipore, Burlington, MA), protease inhibitor cocktail (Sigma Aldrich, St. Louis, MO) and phosphatase inhibitor cocktail (Sigma Aldrich, St. Louis, MO). The lysate was centrifuged at 14,000 g for 15 min at 4 °C. Supernatant was incubated with MagStrep type3 XT beads (IBA Lifesciences, Goettingen, Germany) overnight at 4 °C. Beads were washed three times with lysis buffer, and eluted with BXT buffer (IBA Lifesciences, Goettingen, Germany) overnight at 4 °C. At last, the eluted recombinant proteins were subjected to dialysis using 10 kD Slide-A-Lyzer™ Dialysis Cassettes (Thermo Fisher Scientific, Waltham, MA), concentration with 10 kD Spin Column (Abcam, Cambridge, MA) and purification by size-exclusion chromatography.

### Stereotaxic injection and ICV infusion

Mice surgery were performed according to the NIH guidelines and were approved by the Institutional Animal Care and Use Committee (IACUC) at Case Western Reserve University. All AAVs with 10^13^ genome copies per mL (GC per mL) were obtained from Vigene Biosciences (Jinan, China). For stereotaxic injection, mice were anesthetized with isoflurane and immobilized using the stereotactic frame equipped with a heating blanket to maintain body temperature throughout the procedure. After hair removal and the cleaning of the shaved area with betadine and alcohol, mice were injected with bupivacaine/lidocaine and a small incision was made to expose the skull surface. Two small holes were drilled in the skull (relative to bregma: anteroposterior −2.1 mm, medial lateral ±2 mm; Note that ± is the plus-minus sign throughout this study) followed by injection of 2 μl AAVs using Hamilton syringes into the hippocampal CA1 at dorsal ventral −1.45 mm. Injection speed was pump controlled at 0.2 μl per min. The needle was left in place for 5 min before it was slowly withdrawn. For ICV infusion, the mini-osmotic pump (Model 1004, Alzet, Cupertino, CA; flow rate of 0.11 μl per hour, 28 days) and brain infusion cannula attached with 2.5–3 cm catheter tubes (Brain infusion kit 3, Alzet, Cupertino, CA) were filled with recombinant protein in artificial cerebrospinal fluid (aCSF), followed by pump incubation in aCSF at 37 °C for 48 h according to the manufacturer’s instructions. For implant surgery, a hole was drilled in the skull (relative to bregma: anteroposterior −0.5 mm, medial lateral 0.75 mm). The cannula was positioned on the skull with the needle plug 2.5 mm into the ventricle. The cannula was fixed and secured by cyanoacrylate glue.

### Behavioral tests

Mice behavioral tests were also performed according to the NIH guidelines and were approved by the Institutional Animal Care and Use Committee (IACUC) at Case Western Reserve University. The Barnes maze consisted of a white acrylic circular disk 92 cm in diameter with 20 equally spaced holes (5 cm in diameter) located 2 cm from the edge of the disk. The maze was illuminated by two 60 W lamps to provide an aversive, bright disk surface. An acrylic escape box (7 × 7 × 5 cm) could be fitted under any of the holes in the maze. The maze was raised 30 cm from the floor and rested on a pedestal that enabled it to be rotated 360° on a horizontal plane. An acrylic start bin with 15 cm diameter and 15 cm height was used. Trials were recorded using a webcam and analyzed by video tracking software (EthoVision XT, Noldus, Leesburg, VA). Each trial began with the start bin positioned in the center of the maze with the mouse placed inside. The mouse remained in the start bin for 30 s, providing a standard starting context for each trial and ensuring that initial orientation of the mouse in the maze varied randomly from trial to trial. Each mouse was allowed to explore the maze freely for 2 min. After the mouse entered the escape hole, the mouse was left in the escape box for 90 s before being returned to its home cage. If the mouse did not enter the escape box within 120 s, it was gently picked up by the experimenter and placed over the target hole and allowed to enter the escape box. After each trial, the maze and escape box were cleaned carefully with a 10% alcohol solution to dissipate odor cues and provide a standard olfactory context. Five training sessions consisting of two trials each were run on subsequent days and escape latencies were measured. For Y maze test, mice were placed in a Plexiglas Y maze (with arms 60 cm in length) and allowed to explore the maze freely for 10 min. When put in the Y maze, the mice were recorded using the ANY-maze tracking system, and the time and frequency in the spontaneous alteration ratio were counted automatically. All tests were performed at the Case Behavior Core, with the investigator blinded to mouse genotype.

### Plaque isolation

Amyloid plaque cores were isolated as previously described^55^. Briefly, whole mouse brain or human brain gray matters were homogenized, boiled in lysis buffer (2% SDS, 50 mM Tris-HCl pH 7.5, 50 mM DTT), and centrifuged at 100,000 g for 1 h at 10 °C. The pellet was solubilized in fraction buffer (1% SDS, 50 mM Tris-HCl pH 7.5, 50 mM DTT) and centrifuged at 100,000 g for 1 h at 10 °C. The pellet was further suspended in fraction buffer and loaded on top of a discontinuous sucrose gradient (1.0, 1.2, 1.4 and 2.0 M sucrose in 50 mM Tris pH 7.5 containing 1% SDS), centrifuged at 220,000 g for 20 h at 10 °C and fractionated into sixteen fractions (300 µl per fraction). Plaque-core-enriched fraction #13 were further diluted in fraction buffer and centrifuged at 220,000 g for 2 h at 10 °C. The resulting pellet was dissolved in 70% formic acid and subsequently dried using a SpeedVac system. Solubilized proteins were further resuspended in 1X SDS sample buffer with 8 M Urea.

### Aβ preparation, pull-down, and co-sedimentation assay

Synthetic human Aβ_1–42_ and Aβ_1–40_ peptides (GL Biochem, Shanghai) were dissolved in hydroxyl-fluro-isopro-panol (HFIP) and subsequently dried using a SpeedVac system. Both Aβ_1–42_ and Aβ_1–40_ monomers were prepared by dissolving the lyophilized Aβ in dimethyl sulfoxide (DMSO) at 5 mM, sonicated for 10 min and diluted in PBS buffer (NaCl 137 mM, KCl 2.7 mM, Na_2_HPO_4_ 10 mM, KH_2_PO_4_ 1.8 mM, pH 7.4) to different concentrations. Aβ_1–42_ oligomers were prepared in DMSO/PBS and oligomerized by incubation at 4 °C for 24 or 48 h. Monomeric or oligomer Aβ_1–40_ (100 μM) and Aβ_1–42_ solutions (50 μM) supplemented with or without rAggregatin bound Strevdin-avdin beads were incubated in IP buffer (NaCl 300 mM, KCl 2.7 mM, Na2HPO4 10 mM, KH2PO4 1.8 mM, pH7.4) at RT with shaking for 2 h. After 4 times wash with IP buffer, beads were eluted by 1XSDS sample buffer (32.9 mM Tris-HCl pH6.8, 13% Glycerol, 1% SDS and 0.005 % bromophenol blue) and analyzed by 10–20% SDS/Tricine protein gels (Invitrogen, Carlsbad, CA). For Aβ_1–42_ oligomer formation and co-sedimentation assay, HFIP dissolved synthetic Aβ_1–42_ peptides were solubilized in 30 mM NaOH to a final concentration of 100 μM, diluted to 2.5 μM in PBS and incubated with and without 30 nM rAggregatin at 37 °C for different time points. After 10-minute centrifuge at 14,000 g, pellets and supernatants were collected and analyzed by 10–20% SDS/Tricine protein gels (Invitrogen, Carlsbad, CA).

### Dynamic light scattering

Dynamic light scattering (DLS) experiments were carried out with DynaPro™ instrument from Wyatt technology with a wavelength of 633 nm and a scattering angle of 173°. The measurements of Aggregatin or Aggregatin Δ61–80 at 100 nM were performed at 25 °C after 2 min equilibration with correlation times defined on 10 s per run with 30 runs for each measurement. The results were plotted as intensity of distribution (%) of particles versus hydrodynamic radius (nm).

### Circular dichroisms

The spectra were recorded over a wavelength range of 260–190 nm with standard sensitivity at the 50 nm per min scan speed with 1‐nm resolution and 1‐s time constant at room temperature using a spectropolarimeter (Jasco J-815). All the proteins were dissolved in phosphate buffer (pH8.0). The final concentration of all samples was 1 µM. The secondary structure content was calculated from the Circular dichroisms (CD) spectra using the online software K2D3.

### Surface plasmon resonance

Surface plasmon resonance (SPR) was determined using BIAcore3000 (GE Healthcare Life Sciences, Pittsburgh, PA). rAggregatin (0.1 mg per ml) was immobilized on the CM5 sensor surface (GE Healthcare Life Sciences, Pittsburgh, PA) in 10 mM acetate buffer (pH = 4.5). Running buffer was 1% DMSO in PBS-P buffer (0.02 M phosphate, 2.7 mM KCl, 137 mM NaCl and 0.05% Tween 20). Binding of a dilution series comprising Aβ_1–42_ monomers to rAggregatin was analyzed and fitted to the 1:1 binding model using BIAevaluation software (GE Healthcare Life Sciences, Pittsburgh, PA).

### Solid phase binding assay

rAggregatin was coated onto Nunc MaxiSorp 96-well plates (Thermo Fisher Scientific, Waltham, MA) at 0.1 μg per well in PBS at 4 °C overnight. After blocking in 1% BSA in PBS for 2 h at RT, Aβ_1–42_ at 6.25, 12.5, 25, 50, 100, or 200 nM or Aβ_1–40_ at 0.5, 1, 2, 4, or 8, or 16 μM monomers were added to the plates at 4 °C overnight. Plates were washed with PBS 4 times and incubated with 6E10 antibody at 4 °C overnight, followed by 4 times PBS wash and development in TMB solution (Thermo Fisher Scientific, Waltham, MA). The reaction was stopped by sulfuric acid and assessed using a Synergy H1 microplate reader (BioTek, Winooski, VT). Likewise, 0.2 μg Aβ_1–42_ or Aβ_1–40_ monomers were immobilized on plates and incubated with 3.125, 6.25, 12.5, 25, 50, or 100 nM rAggregatin. Bound rAggregatin were detected by an anti-Flag antibody and developed in TMB solution as described above.

### ThT fluorescence assay

HFIP treated Aβ_1–40_ or Aβ_1–42_ peptides were solubilized in 30 mM NaOH to a final concentration of 400 μM, sonicated for 5 min in a water bath and stored at −80 °C until further use. To monitor Aβ_1–40_ and Aβ_1–42_ fibrillization, a ThT assay was performed according previous studies^56,57^. Briefly, a stock solution of Aβ was diluted to in PBS with 20 μM ThT. Then rAggregatin were added at desired concentrations to the final volume of 100 µl. All samples were transferred to a black 96-well nonbinding Surface microplate with clear bottom (Corning, Corning, NY), and sealed with a polyester-based sealing film (Corning, Corning, NY). Samples were incubated at 37 °C with stirring. Real-time ThT fluorescence was measured every 5 min for at least 12 h at the excitation and emission wavelengths of 446 nm and 482 nm respectively by a Synergy H1 microplate reader (BioTek, Winooski, VT).

### Aβ_1–42_ aggregates stained by Thio-S

To evaluate Aβ aggregates formed in vitro, rAggregatin (30 nM) and 2.5 μM Aβ in PBS were incubated at 37 °C for 4 weeks. 20 μl of protein solution were applied to the glass slides and completely air dry for 30 min. After washing with PBS, the samples were stained by 1% Thio-S for 10 min. The 3D confocal images were analyzed by using Imaris (Bitplane, Concord, MA) and the structure surface were extracted by using the SURFACE tools following the manufacturer’s instructions.

### Negative electric microscopy

HFIP dissolved synthetic Aβ_1–42_ peptides were solubilized in 30 mM NaOH to a final concentration of 100 μM. Then diluted to 2.5 μM in PBS and incubated with and without 30 nM rAggregatin at 37 °C. Immediately following the indicated incubation time, 20 μl of protein solution were applied to the support surface of the grids, which were autoclaved by UV irradiation overnight. The grids were washed with 20 μl droplets of water 4 times, followed by a 20 μL droplet of uranyl acetate solution, then examined in an FEI Tecnai Spirit (T12) with a Gatan US4000 4kx4k CCD.

### Total Aβ measurement by ELISA

Brains were homogenized in TBS Buffer (50 mM Tris-HCl and 150 mM NaCl, pH 7.6) containing 1 mM PMSF (Millipore, Burlington, MA), protease inhibitor cocktail (Sigma Aldrich, St. Louis, MO) and phosphatase inhibitor cocktail (Sigma Aldrich, St. Louis, MO). Total protein concentrations were determined using the BCA kit (Thermo Fisher Scientific, Waltham, MA). ELISA of total Aβ was carried out in 96-well high-binding microtiter plates. Monoclonal antibody 6E10 raised against residues Aβ1–16 was used as a capture antibody (diluted in PBS pH 7.4) and incubated over night at 4 °Cin a humid chamber. After removal of the capture antibody, the plate surface was blocking with 1% BSA for 1.5 h. After washing with PBS, 0.5 µg total protein were added and incubated at 4 °Covernight. Monoclonal antibody MOAB-2 coupled to horseradish peroxidase diluted in PBS were used as secondary antibodies and again incubated over night at 4 °C. After three times washing with PBS, 100 μl of TMB ELISA peroxidase substrate (Thermo Fisher Scientific, Waltham, MA) was added and incubated for 1–10 min at RT in darkness. The reaction was stopped with 100 μl 2 M H_2_SO_4_ and absorbance was measured in a microplate reader at 450 nm. For generation of standard curves, synthetic Aβ1–42 peptides freshly dissolved in DMSO from 1 ng per µL to 10 pg per µL.

### Isolation of exosomes

Lenti-293T cells were transfected with empty vector or pCDNA-4xFlag-Aggregatin using TransIT®−293 Transfection Reagent (Mirus, Madison, WI). Twenty-four hours after transfection, cells were cultured in the DMEM medium supplemented with exosome-free FBS. Forty-eight hours later, the cell culture medium was collected and centrifuged at 300 g for 15 min to remove cells and debris. The supernatant was further filtered through a 0.22 μm filter and centrifuged at 100,000 g for 2 h at 4 °C. The pellets enriched with exosomes were resuspended in the lysis buffer (100 mM Tris-HCl, 150 mM NaCl, 1 mM EDTA and 1% NP40, pH 8.0) containing 1 mM PMSF (Millipore, Burlington, MA), protease inhibitor cocktail (Sigma Aldrich, St. Louis, MO), and phosphatase inhibitor cocktail (Sigma Aldrich, St. Louis, MO) followed by immunoblot analysis.

### Confocal microscopy and image analysis

All fluorescence images were imaged on a Leica TCS SP8 gSTED confocal microscopy (Leica Microsystems, Buffalo Grove, IL) equipped with a motorized super Z galvo stage, two PMTs, three Hyd SP GaAsP detectors for gated imaging, and the AOBS system lasers including a 405 nm, Argon (458, 476, 488, 496, 514 nm), a tunable white light (470 to 670 nm), and a 592 nm STED depletion laser. Series of confocal images with optical thickness of 300 nm were collected using the ×100 oil objective. All 3D confocal images of plaque were reconstructed using Imaris (Bitplane, Concord, MA) after background subtraction. Quantification of Aggregatin foci in plaques and measurement of plaque load and size were performed with open-source image analysis programs WCIF ImageJ (developed by W. Rasband).

### Statistical analysis

Statistical analysis was done with one-way analysis of variance (ANOVA) followed by Tukey’s multiple comparison test or student-t-test using GraphPad Prism (GraphPad, CA). Data are means ± SEM. *p* < 0.05 was considered to be statistically significant.

### Reporting summary

Further information on research design is available in the Nature Research Reporting Summary linked to this article.

## Supplementary information

Supplementary Information

Description of Additional Supplementary Files

Supplementary Data 1

Reporting Summary

## Data Availability

The data that support the findings of this study are available on request from the corresponding author X.L.W. Case Western Reserve University supports the NIH Guidelines for the Sharing of Research Resources including “the Sharing of Biomedical Research Resources: Principles and Guidelines for Recipients of NIH Grants and Contracts”. If any intellectual property is pursued, the data will be shared and distributed following advice from the authorities of Case Western Reserve University. The source data underlying Figs. 1, 2g, 2h, 3a–d, 3f, 3g, 4a–f, 5c–f, 5h–k, 5m–p, and Supplementary Figs. 3a–e, 4, 5c–h, 7d, 7e, 7i–k, 8b, 8d, 9a–d, 10b, 10c, 10e, 11b–f, 12a, 12c–e, 13a–d,14, 15e–h, 16, 17b–d, 18b, 18c, 19a, 19c, and 19e are provided as a Source Data file. Source Data for GWAS in Fig. 1: 10.6084/m9.figshare.11336657 (https://figshare.com/s/2d59f2f0b7cfd04d31ca). Source Data for GWAS in Supplementary Fig. 2a: 10.6084/m9.figshare.11336666 (https://figshare.com/s/c094e1a5c2ea562b6aef). Source Data for GWAS in Supplementary Fig. 2b: 10.6084/m9.figshare.11337272 (https://figshare.com/s/1449069e2b80a75b30cf). Source Data for GWAS in Supplementary Fig. 2c: 10.6084/m9.figshare.11337281 (https://figshare.com/s/4cc10442ea2f51333cd7). Source Data for GWAS in Supplementary Fig. 2d: 10.6084/m9.figshare.11337284 (https://figshare.com/s/220153dbfce30bca2802). Source Data for GWAS in Supplementary Fig. 2e: 10.6084/m9.figshare.11337290 (https://figshare.com/s/8a1f80899f69b462f284).

## Source data

Source Data
