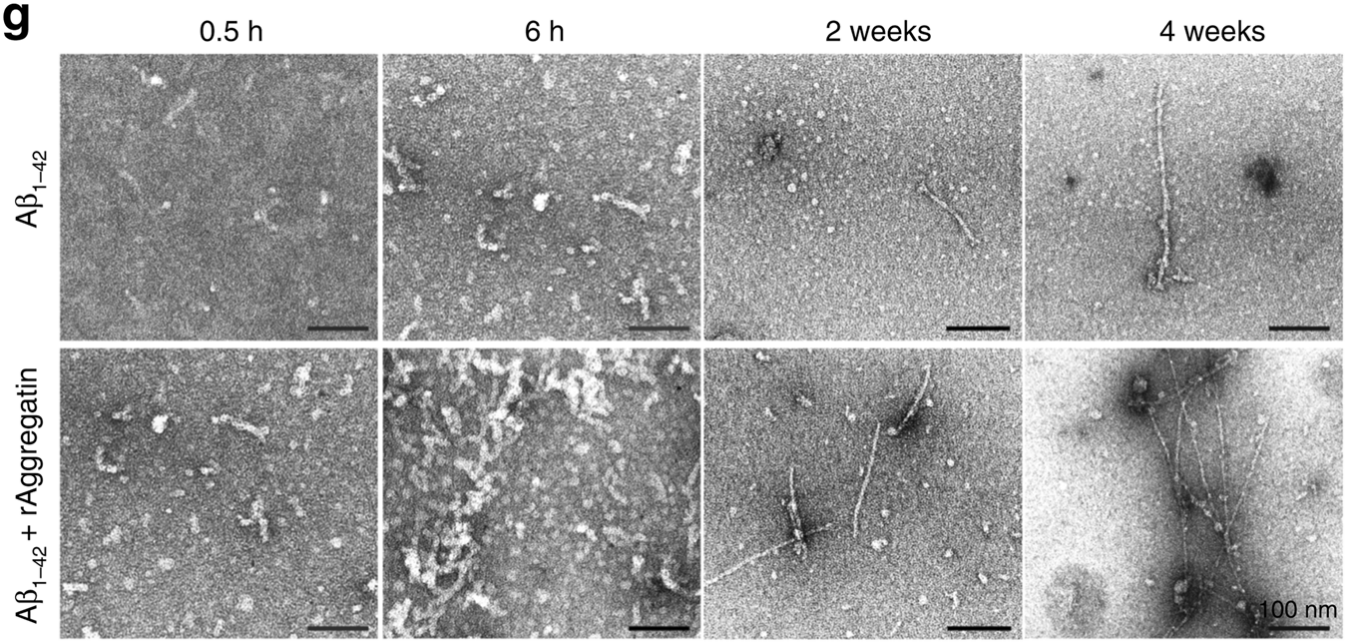# Author Correction: *FAM222A* encodes a protein which accumulates in plaques in Alzheimer’s disease

**DOI:** 10.1038/s41467-022-31711-8

**Published:** 2022-07-11

**Authors:** Tingxiang Yan, Jingjing Liang, Ju Gao, Luwen Wang, Hisashi Fujioka, The Alzheimer Disease Neuroimaging Initiative, Michael W. Weiner, Norbert Schuff, Howard J. Rosen, Bruce L. Miller, David Perry, Paul Aisen, Arthur W. Toga, Gustavo Jimenez, Michael Donohue, Devon Gessert, Kelly Harless, Jennifer Salazar, Yuliana Cabrera, Sarah Walter, Lindsey Hergesheimer, Arthur W. Toga, Karen Crawford, Scott Neu, Lon S. Schneider, Sonia Pawluczyk, Mauricio Becerra, Liberty Teodoro, Bryan M. Spann, Paul Aisen, Ronald Petersen, Clifford R. Jack, Matthew Bernstein, Bret Borowski, Jeff Gunter, Matt Senjem, Prashanthi Vemuri, David Jones, Kejal Kantarci, Chad Ward, Sara S. Mason, Colleen S. Albers, David Knopman, Kris Johnson, Neill R. Graff-Radford, Francine Parfitt, Kim Poki-Walker, William Jagust, Susan Landau, John Q. Trojanowki, Leslie M. Shaw, Jason H. Karlawish, David A. Wolk, Sanjeev Vaishnavi, Christopher M. Clark, Steven E. Arnold, Virginia Lee, Magdalena Korecka, Michal Figurski, Laurel Beckett, Danielle Harvey, Charles DeCArli, Evan Fletcher, Pauline Maillard, John Olichney, Owen Carmichael, Robert C. Green, Reisa A. Sperling, Keith A. Johnson, Gad A. Marshall, Andrew J. Saykin, Tatiana M. Foroud, Li Shen, Kelley Faber, Sungeun Kim, Kwangsik Nho, Martin R. Farlow, Ann Marie Hake, Brandy R. Matthews, Jared R. Brosch, Scott Herring, John Morris, Marc Raichle, David Holtzman, John C. Morris, Nigel J. Cairns, Erin Franklin, Lisa Taylor-Reinwald, Beau Ances, David Winkfield, Maria Carroll, Angela Oliver, Mary L. Creech, Mark A. Mintun, Stacy Schneider, Lew Kuller, Chet Mathis, Oscar L. Lopez, MaryAnn Oakley, Donna M. Simpson, Steven Paul, Norman Relkin, Gloria Chiang, Michael Lin, Lisa Ravdin, Peter Davies, M. Marcel Mesulam, Marek-Marsel Mesulam, Emily Rogalski, Kristine Lipowski, Sandra Weintraub, Borna Bonakdarpour, Diana Kerwin, Chuang-Kuo Wu, Nancy Johnson, Peter J. Snyder, Tom Montine, Michael Donohue, Lean Thal, James Brewer, Helen Vanderswag, Adam Fleisher, Paul Thompson, Ellen Woo, Daniel H. S. Silverman, Edmond Teng, Sarah Kremen, Liana Apostolova, Kathleen Tingus, Po H. Lu, George Bartzokis, Robert A. Koeppe, Jaimie Ziolkowski, Judith L. Heidebrink, Joanne L. Lord, Norm Foster, Marilyn Albert, Chiadi Onyike, Daniel D’Agostino, Stephanie Kielb, Joseph Quinn, Lisa C. Silbert, Betty Lind, Jeffrey A. Kaye, Raina Carter, Sara Dolen, Javier Villanueva-Meyer, Valory Pavlik, Nathaniel Pacini, Ashley Lamb, Joseph S. Kass, Rachelle S. Doody, Victoria Shibley, Munir Chowdhury, Susan Rountree, Mimi Dang, Yaakov Stern, Lawrence S. Honig, Karen L. Bell, Randy Yeh, Daniel Marson, David Geldmacher, Marissa Natelson, Randall Griffith, David Clark, John Brockington, Hillel Grossman, Effie Mitsis, Raj C. Shah, Melissa Lamar, Patricia Samuels, Martin Sadowski, Mohammed O. Sheikh, Jamika Singleton-Garvin, Anaztasia Ulysse, Mrunalini Gaikwad, P. Murali Doraiswamy, Olga James, Salvador Borges-Neto, Terence Z. Wong, Edward Coleman, Charles D. Smith, Greg Jicha, Peter Hardy, Riham El Khouli, Elizabeth Oates, Gary Conrad, Anton P. Porsteinsson, Kim Martin, Nancy Kowalksi, Melanie Keltz, Bonnie S. Goldstein, Kelly M. Makino, M. Saleem Ismail, Connie Brand, Gaby Thai, Aimee Pierce, Beatriz Yanez, Elizabeth Sosa, Megan Witbracht, Steven Potkin, Kyle Womack, Dana Mathews, Mary Quiceno, Allan I. Levey, James J. Lah, Janet S. Cellar, Jeffrey M. Burns, Russell H. Swerdlow, William M. Brooks, Christopher H. van Dyck, Richard E. Carson, Pradeep Varma, Howard Chertkow, Howard Bergman, Chris Hosein, Raymond Scott Turner, Kathleen Johnson, Brigid Reynolds, Neil Kowall, Ronald Killiany, Andrew E. Budson, Alexander Norbash, Patricia Lynn Johnson, Thomas O. Obisesan, Ntekim E. Oyonumo, Joanne Allard, Olu Ogunlana, Alan Lerner, Paula Ogrocki, Curtis Tatsuoka, Parianne Fatica, Sterling Johnson, Sanjay Asthana, Cynthia M. Carlsson, Jerome Yesavage, Joy L. Taylor, Steven Chao, Barton Lane, Allyson Rosen, Jared Tinklenberg, Douglas W. Scharre, Maria Kataki, Rawan Tarawneh, Earl A. Zimmerman, Dzintra Celmins, David Hart, Laura A. Flashman, Marc Seltzer, Mary L. Hynes, Robert B. Santulli, Kaycee M. Sink, Mia Yang, Akiva Mintz, Delwyn D. Miller, Karen Ekstam Smith, Hristina Koleva, Ki Won Nam, Hyungsub Shim, Susan K. Schultz, Amanda Smith, Christi Leach, Balebail Ashok Raj, Kristin Fargher, Eric M. Reiman, Kewei Chen, Pierre Tariot, Anna Burke, Joel Hetelle, Kathryn DeMarco, Nadira Trncic, Adam Fleisher, Stephanie Reeder, Edward Zamrini, Christine M. Belden, Sherye A. Sirrel, Ranjan Duara, Maria T. Greig-Custo, Rosemarie Rodriguez, Charles Bernick, Donna Munic, Zaven Khachaturian, Neil Buckholtz, John Hsiao, William Potter, Howard Fillit, Franz Hefti, Carl Sadowsky, Teresa Villena, Ging-Yuek Robin Hsiung, Benita Mudge, Vesna Sossi, Howard Feldman, Michele Assaly, Elizabeth Finger, Stephen Pasternack, William Pavlosky, Irina Rachinsky, Dick Drost, Andrew Kertesz, Sandra Black, Bojana Stefanovic, Chrinthaka Heyn, Brian R. Ott, Geoffrey Tremont, Lori A. Daniello, Courtney Bodge, Stephen Salloway, Paul Malloy, Stephen Correia, Athena Lee, Godfrey D. Pearlson, Karen Blank, Karen Anderson, Vernice Bates, Horacio Capote, Michelle Rainka, Jacobo Mintzer, Kenneth Spicer, David Bachman, Elizabeth Finger, Stephen Pasternak, Irina Rachinsky, John Rogers, Andrew Kertesz, Dick Drost, Elizabeth Finger, Stephen Pasternak, Irina Rachinsky, John Rogers, Andrew Kertesz, Dick Drost, Nunzio Pomara, Raymundo Hernando, Antero Sarrael, Smita Kittur, Michael Borrie, T.-Y. Lee, Rob Bartha, Richard Frank, Nick Fox, Veronika Logovinsky, Maria Corrillo, Greg Sorensen, Xiaofeng Zhu, Xinglong Wang

**Affiliations:** 1 Department of Pathology, Case Western Reserve University, Cleveland, OH USA; 2 Department of Population and Quantitative Health Sciences, Case Western Reserve University, Cleveland, OH USA; 3 Electron Microscopy Core Facility, Case Western Reserve University, Cleveland, OH USA; 4 University of California, San Francisco, CA USA; 5 University of Southern California, San Francisco, CA USA; 6 Mayo Clinic, Rochester, MN USA; 7 Mayo Clinic, Jacksonville, FL USA; 8 University of California, Berkeley, CA USA; 9 University of Pennsylvania, Philadelphia, PA USA; 10 University of California, Davis, CA USA; 11 Brigham and Women’s Hospital, Boston, MA USA; 12 Indiana University, Indianapolis, IN USA; 13 Washington University, St. Louis, MO USA; 14 University of Pittsburgh, Pittsburgh, PA USA; 15 Cornell University, New York, NY USA; 16 Albert Einstein College of Medicine of Yeshiva University, New York, NY USA; 17 Northwestern University, Chicago, IL USA; 18 Brown University, Providence, RI USA; 19 University of Washington, Seattle, WA USA; 20 University of California, San Diego, CA USA; 21 University of California, Los Angeles, CA USA; 22 University of Michigan, Ann Arbor, MI USA; 23 University of Utah, Salt Lake City, UT USA; 24 Johns Hopkins University, Baltimore, MD USA; 25 Oregon Health & Science University, Portland, OR USA; 26 Baylor College of Medicine, Houston, TX USA; 27 Columbia University Medical Center, New York, NY USA; 28 University of Alabama, Birmingham, AL USA; 29 Mount Sinai School of Medicine, New York, NY USA; 30 Rush University Medical Center, Chicago, IL USA; 31 New York University, New York, NY USA; 32 Duke University Medical Center, Durham, NC USA; 33 University of Kentucky, Lexington, KY USA; 34 University of Rochester Medical Center, Rochester, NY USA; 35 University of California, Irvine, CA USA; 36 University of Texas Southwestern Medical School, Dallas, TX USA; 37 Emory University, Atlanta, GA USA; 38 University of Kansas Medical Center, Kansas City, KS USA; 39 Yale University School of Medicine, New Haven, CT USA; 40 McGill University Montreal-Jewish General Hospital, Montreal, QC Canada; 41 Georgetown University Medical Center, Washington, DC USA; 42 Boston University, Boston, MA USA; 43 Howard University, Washington, DC USA; 44 University Hospitals, Cleveland, OH USA; 45 University of Wisconsin, Madison, WI USA; 46 Stanford University, Stanford, CA USA; 47 Ohio State University, Columbus, OH USA; 48 Albany Medical College, Albany, NY USA; 49 Dartmouth-Hitchcock Medical Center, Lebanon, NH USA; 50 Wake Forest University Health Sciences, Winston-Salem, NC USA; 51 University of Iowa College of Medicine, Iowa City, IA USA; 52 University of South Florida, Health Byrd Alzheimer’s Institute, Tampa, FL USA; 53 Banner Alzheimer’s Institute, Phoenix, AZ USA; 54 Banner Sun Health Research Institute, Sun City, AZ USA; 55 Wien Center, Miami Beach, FL USA; 56 Cleveland Clinic Lou Ruvo Center for Brain Health, Cleveland, OH USA; 57 Prevent Alzheimer’s Disease, Rockville, MD 2020 USA; 58 National Institute on Aging, Baltimore, MD USA; 59 National Institute of Mental Health, Rockville, MD USA; 60 AD Drug Discovery Foundation, New York, NY USA; 61 Acumen Pharmaceuticals, Livermore, CA USA; 62 Premiere Research Institute, Palm Beach Neurology, West Palm Beach, FL USA; 63 U.B.C. Clinic for AD & Related Disorders, Vancouver, BC Canada; 64 Cognitive Neurology - St. Joseph’s, London, ON Canada; 65 Sunnybrook Health Sciences, Vancouver, ON Canada; 66 Rhode Island Hospital, Providence, RI USA; 67 Butler Hospital, Butler, PA USA; 68 Hartford Hospital, Olin Neuropsychiatry Research Center, Hartford, CT USA; 69 Dent Neurologic Institute, Orchard Park, NY USA; 70 Medical University South Carolina, Charleston, SC USA; 71 St. Joseph’s Health Care, London, ON Canada; 72 Nathan Kline Institute, Orangeburg, NY USA; 73 Neurological Care of CNY, Liverpool, NY USA; 74 Parkwood Institute, London, ON USA; 75 Richard Frank Consulting, London, UK; 76 University of London, London, UK; 77 Eli Lilly and Company, Indianapolis, IN USA; 78 Alzheimer’s Association, Chicago, IL USA; 79 Siemens, Henkestr, DE Germany

**Keywords:** Genome-wide association studies, Alzheimer's disease

Correction to: *Nature Communications* 10.1038/s41467-019-13962-0, published online 21 January 2020.

In the original version of the manuscript, the image shown in Figure 4g, bottom row (Aβ1–42 + rAggregatin), under “6h” was incorrect. This image incorrectly showed the same sample as shown in the original Figure 4g, top row (Aβ1–42), under “0.5h”.

The correct version of figure 4g is as follows:
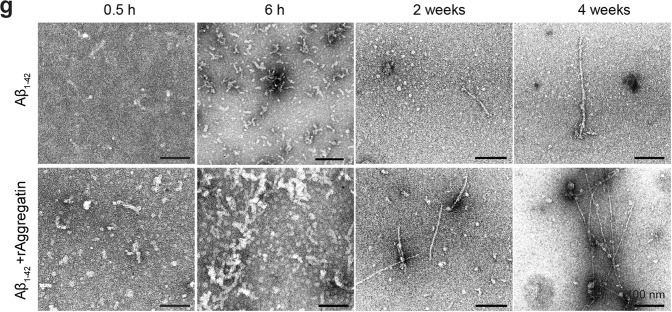


which replaces the previous incorrect version: