# Supplementary material for: FAM222A encodes a protein which accumulates in plaques in Alzheimer’s disease
Source: Nat Commun. 2020 Jan 21;11:411. doi: 10.1038/s41467-019-13962-0 (PMC6972869; doi:10.1038/s41467-019-13962-0)
Supplement: Supplementary file 1 — Supplementary Information [file 41467_2019_13962_MOESM1_ESM.pdf]

Supplementary information

FAM222A encodes a protein which accumulates in plaques in Alzheimer’s disease

Yan et al.

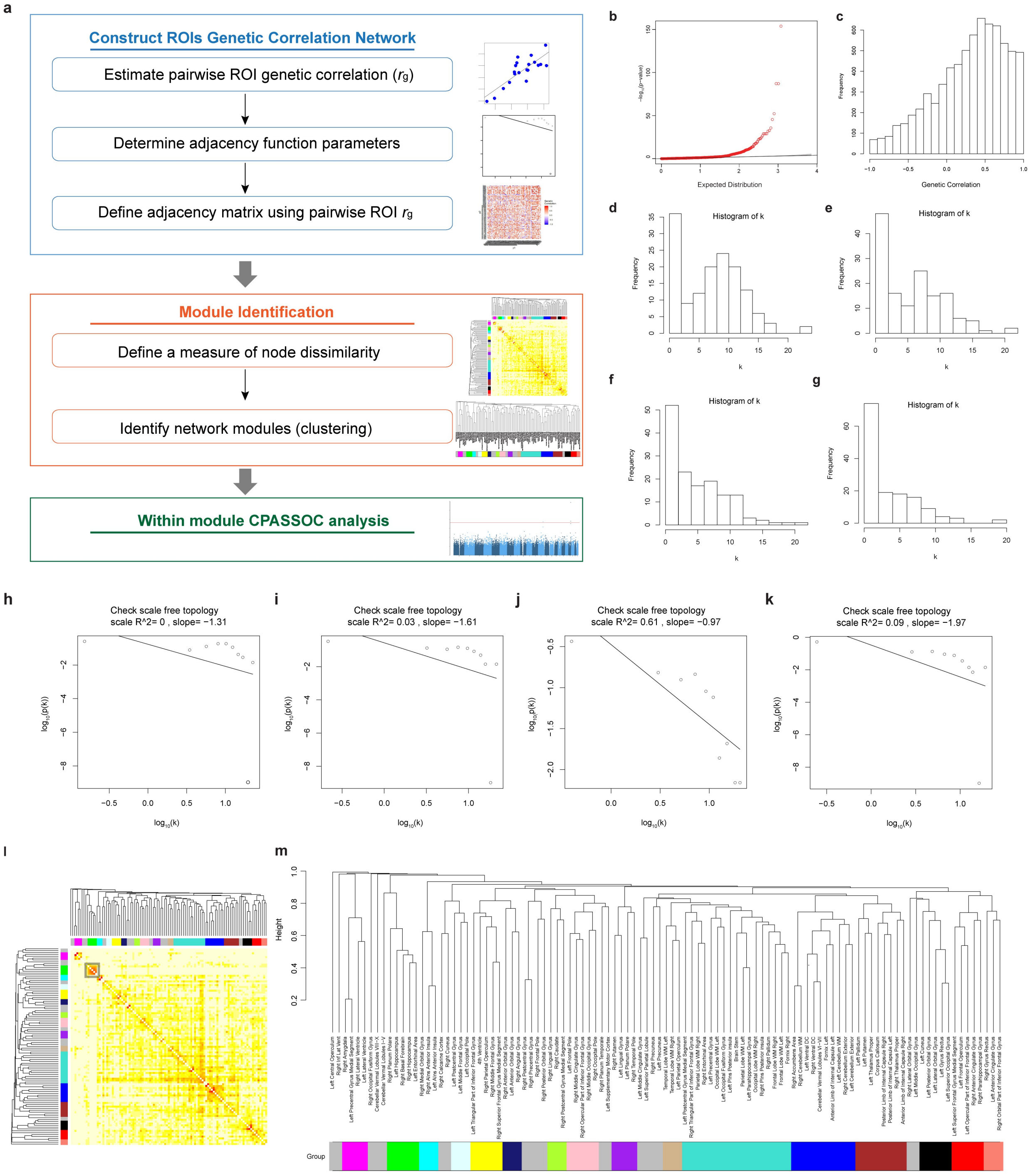

Supplementary Figure 1. Genetic correlation network analysis of brain ROIs volume. (**a**) The flowchart of genetic correlation network and CPASSOC analysis. (**b**) Quantile-quantile plot for expected versus observed *P*-values from pairwise tests of genetic correlation of volumes of 145 ROIs spanning the entire brain. (**c**) Histogram of estimated pairwise ROI genetic correlations. (**d–k**) Histogram of connectivity k (d–g) and scale free topology plot (h–k) of estimated genetic correlation network using different threshold of *P*-values. Pairwise ROIs genetic correlations estimated by LDSC method are selected by different *P*-value threshold to generate ROI genetic correlation network. Genetic network using genetic correlation with *P*-

value < 0.5 is shown in d and h; *P*-value < 0.3 in e and i; *P*-value < 0.2 in f and j; *P*-value < 0.1 in g and k. (**l**) Topological overlap matrix heatmap of genetic correlation network among MRI volumes of 105 brain ROIs. Light color represents low overlap and progressively darker red color represents high overlap. Blocks of darker colors along the diagonal show identified modules. The dendrograms on the upper and left sides outline the hierarchical clustering of ROIs and module assignments. The diagonal block framed by gray lines corresponds to the green-colored module comprising ROIs of left and right hippocampus, right basal forebrain, left entorhinal area and right planum polare. (**m**) Hierarchical clustering dendrogram and module assignment for brain ROIs genetic correlation network**.** Topological overlap dissimilarity measure is clustered by average linkage hierarchical clustering. And module assignments (dynamic hybrid algorithm) are denoted in the color bar (bottom). ROIs are clustered into 16 modules. The gray color is reserved for genes outside of all modules. The green-color module comprises ROIs of left and right hippocampus, right basal forebrain, left entorhinal area and right planum polare. Source data are provided as a Source Data file (Source Data for Statistics and Blots).

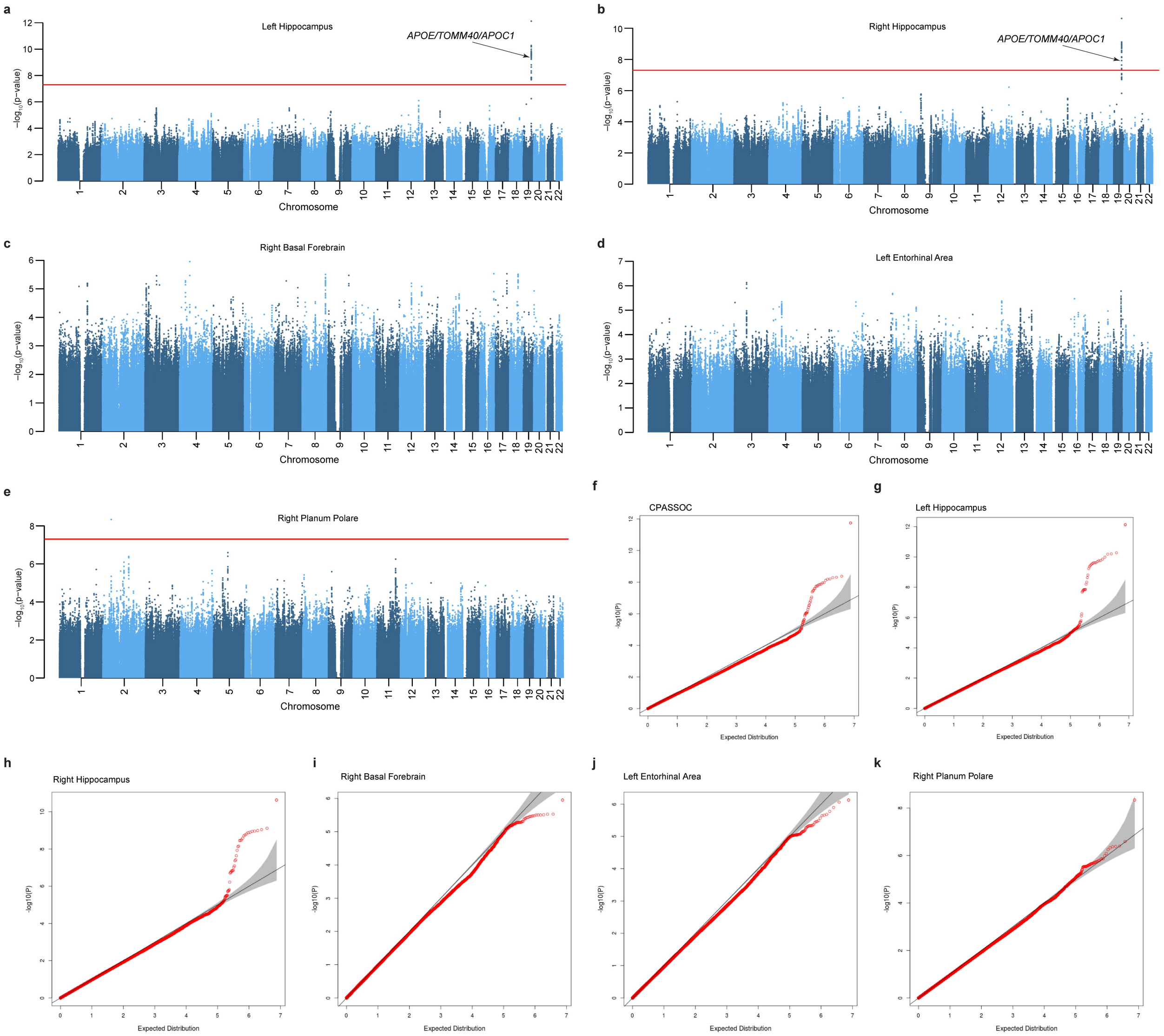

Supplementary Figure 2. Single ROI volume GWAS. (**a–e**) Manhattan plot of single ROI GWAS of left hippocampus (a), right hippocampus (b), right basal forebrain (c), left entorhinal area (d), and right planum polare (e). The red lines denote the genome-wide significant cutoff of $P = 5.0\times10^{-8}$. (**f–k**) Quantile-quantile plots of CPASSOC analysis (f) and individual ROI GWAS of left hippocampus (g), right hippocampus (h), right basal forebrain (i), left entorhinal area (j), and right planum polare (k). Source data are provided as a Source Data file (Source Data for GWAS in Supplementary Fig. 2a, Source Data for GWAS in Supplementary Fig. 2b, Source Data for GWAS in Supplementary Fig. 2c, Source Data for GWAS in Supplementary Fig. 2d, and Source Data for GWAS in Supplementary Fig. 2e).

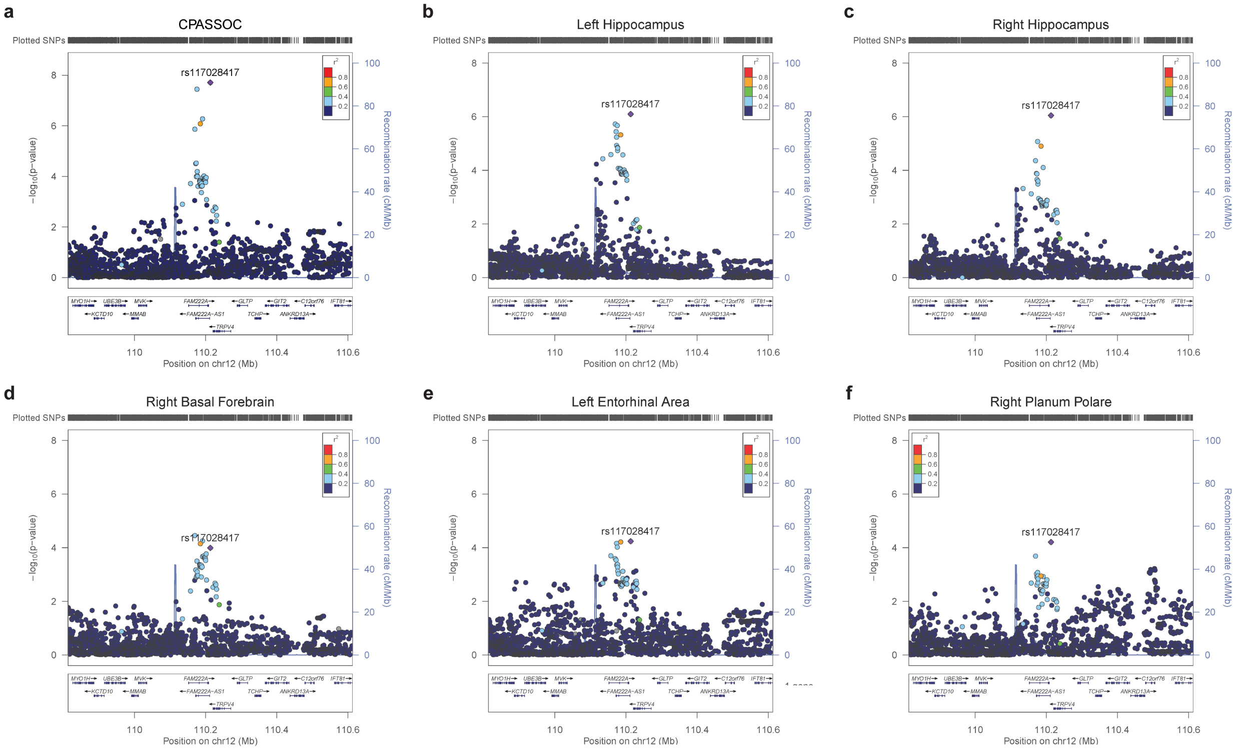

Supplementary Figure 3. Regional association plots. (**a**) Regional association plots of SNP rs117028417 at *12q24.11* in CPASSOC analysis combining 5 ROIs in the green-colored module in Fig. S2M. (**b–f**) Regional association plots of SNP rs117028417 at *12q24.11* in single ROI GWAS in the green-colored module in Fig. S2M: left hippocampus (b), right hippocampus (c), right basal forebrain (d), left entorhinal area (e), and right planum polare (f). Source data are provided as a Source Data file (Source Data for Statistics and Blots).

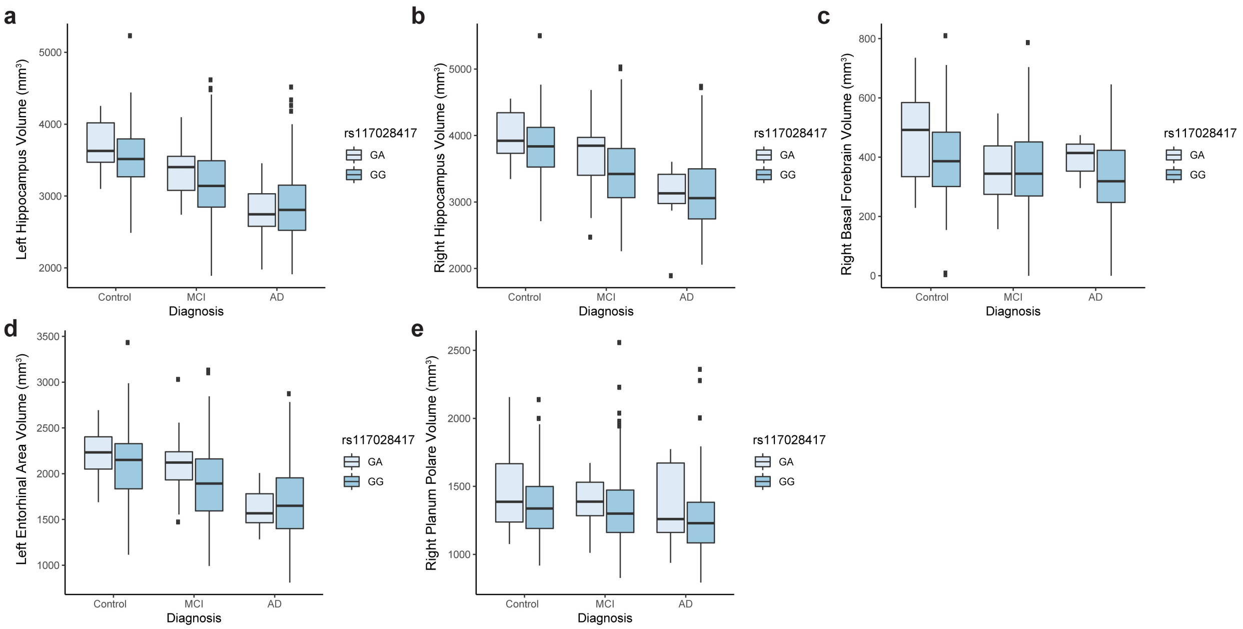

Supplementary Figure 4. Single ROI association with AD. (**a–e**) Refined analysis of ROI volumes in relation to rs117028417 on *FAM222A* and baseline diagnosis for left hippocampus (a), right hippocampus (b), right basal forebrain (c), left entorhinal area (d) and right planum polare (e). Two-way ANOVAs are applied to examine the effects of rs117028417 on *FAM222A* and baseline diagnosis on 5 ROIs. The analysis includes age, gender, education and 10 principal components for ancestry adjustment as covariates. Subjects involved are: 175 AD (7 GA, 168 GG); 366 MCI (17 GA, 349 GG); and 212 CONTROL (15 GA, 197 GG). The p-values for the main effect of diagnosis (DX), the main effect of SNP and the interaction effect of SNP-by-diagnosis (DX×SNP) for each ROI are: DX: $P < 0.001$, SNP: P = 0.006, DX×SNP: $P = 0.665$ for left hippocampus; DX: $P < 0.001$, SNP: $P = 0.007$, DX×SNP: $P = 0.636$ for right hippocampus; DX: $P < 0.001$, SNP: $P = 0.009$, DX×SNP: $P = 0.351$ for right basal forebrain; DX: $P < 0.001$, SNP: $P = 0.061$, DX×SNP: $P = 0.653$ for left entorhinal area; and DX: $P < 0.001$, SNP: $P = 0.069$, DX×SNP: $P = 0.345$ for right planum polare. Source data are provided as a Source Data file (Source Data for Statistics and Blots).

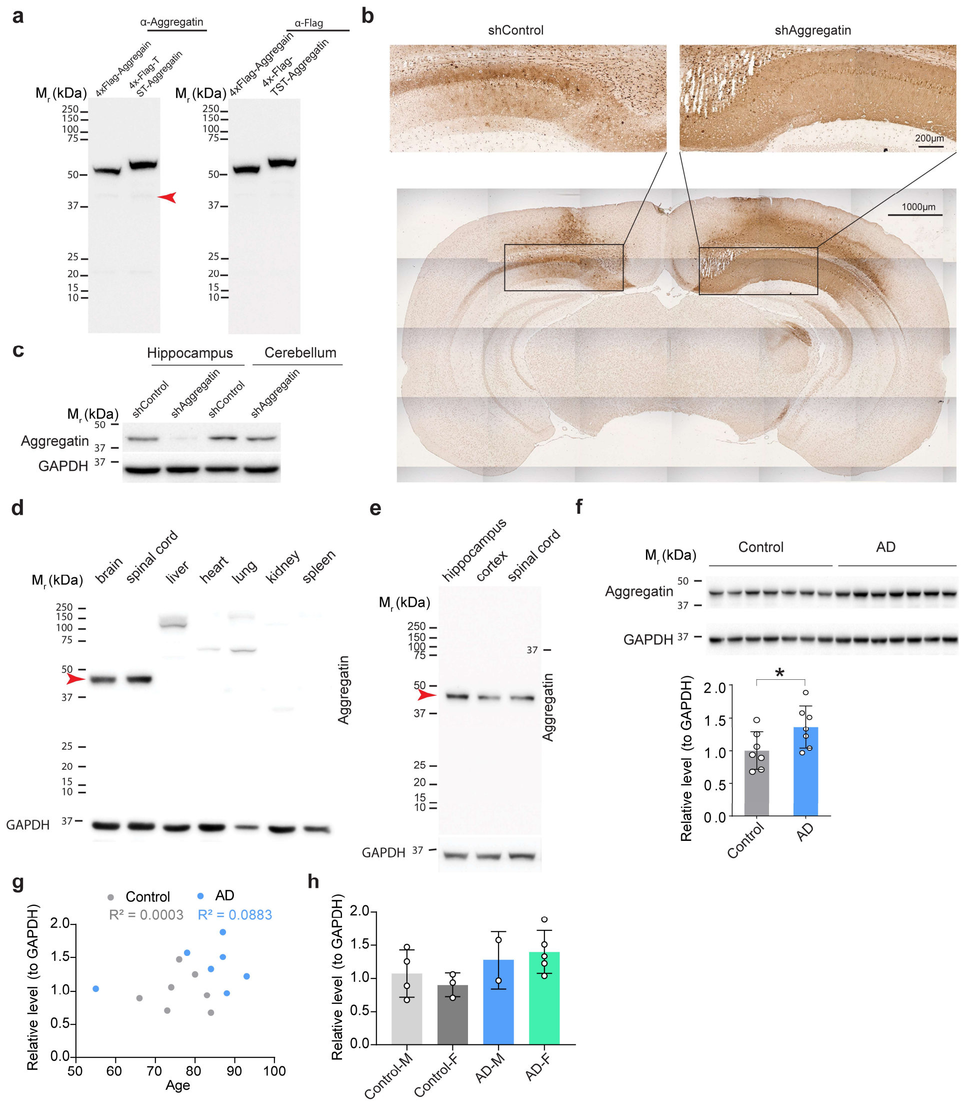

Supplementary Figure 5. Expression of Aggregatin in the CNS. (**a**) Representative immunoblot of Aggregatin by either the Aggregatin antibody (left panel) or Flag antibody (right panel) in HEK293 cells expressing indicated tagged human Aggregatin. The arrow points the faint bands denoting endogenous Aggregatin. (**b**) Representative immunohistochemistry of GFP in 3 month-old 5XFAD mice injected with 2 μl AAV1-shAggregatin or AAV1-shControl into the left and right hippocampal CA1 respectively and scarified 4 weeks later. (**c**) Representative immunoblot of Aggregatin protein levels in the hippocampus and cerebellum of 3 month-old mice injected with 2 μl AAV1-shControl or AAV1-shAggregatin into the left and right hippocampal CA1 respectively and scarified 4 weeks later. (**d** and **e**) Representative immunoblot of Aggregatin protein levels in different tissues of a wild type 6-month old mouse (d) or normal human subject (e). (**f–h**) Representative immunoblot (f), quantification (f) and statistical analysis (g and h) of Aggregatin levels in AD cortices (n = 7 biologically independent samples) compared with age-matched controls (n = 7 biologically independent samples). Source data are provided as a Source Data file (Source Data for Statistics and Blots). Data are means ± s.e.m. All experiments were independently performed at least three times. For "**f**", P-value of logistic regression association analysis between AD and Aggregation expression levels is 0.103 (Odds Ratio = 1.61). The analysis includes age and gender as covariates. ns, non-significant. Student's t-test. *P < 0.05.

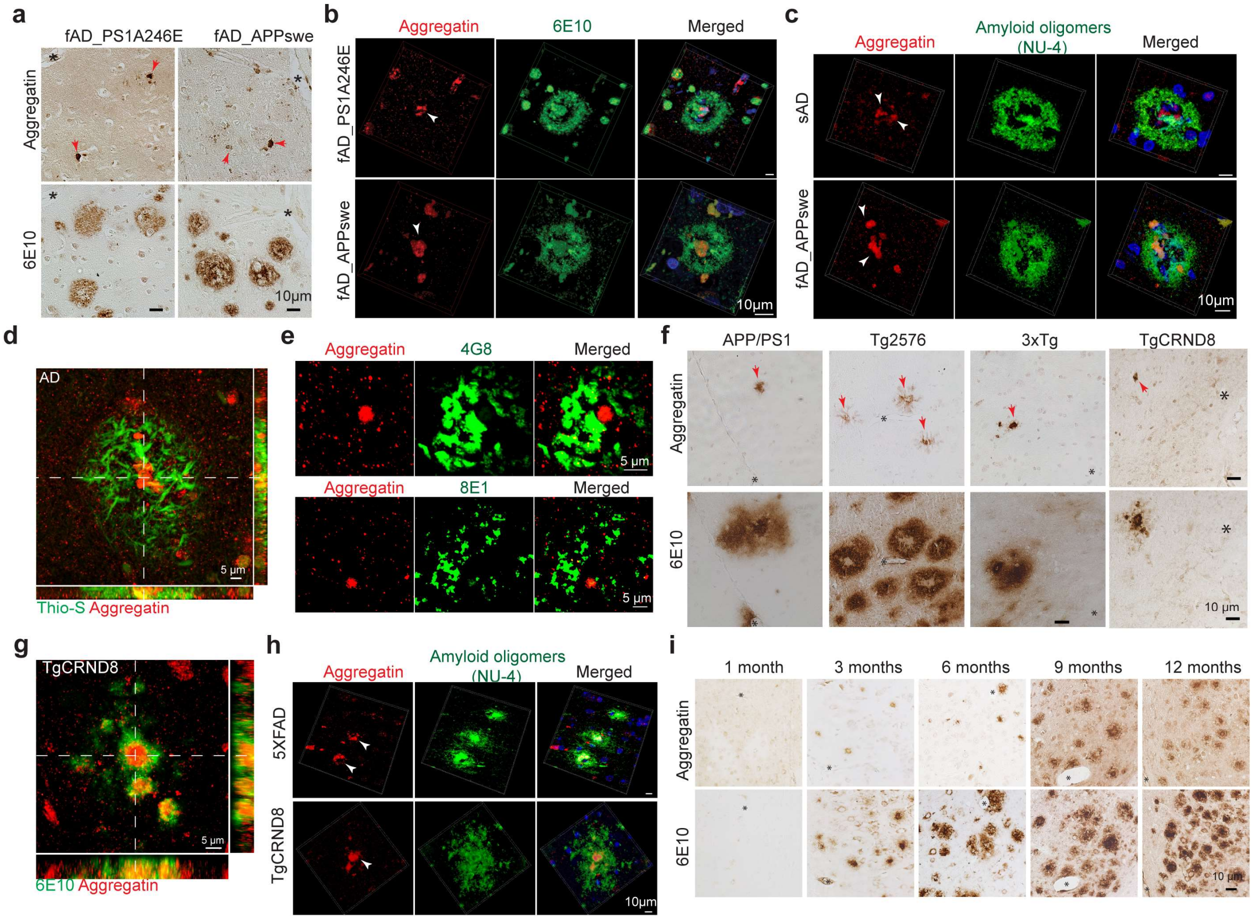

Supplementary Figure 6. Aggregatin accumulates within the center of amyloid plaques in AD and APP transgenic mice for AD. (**a**) Representative images of immunohistochemistry of Aggregatin (arrowheads) and amyloid plaques (stained by the 6E10 antibody) in adjacent sections (denoted by asterisks) of cortices of a familial AD (fAD) patient bearing PS1A246E mutation (fAD_PS1A246E) or a fAD patient bearing APP Swedish mutation (fAD_APPswe). (**b** and **c**) Representative images of Aggregatin foci (Red), amyloid plaques (Green, stained by the 6E10 (b) or NU-4 (c) antibody) and DAPI nuclei staining (Blue) in cortices of sporadic AD (sAD) or fAD patients. (**d**) Representative images of Aggregatin (Red), amyloid plaques (Green, stained by Thio-S) and DAPI nuclei staining (Blue) in cortices of sporadic AD patients. (**e**) Representative images of Aggregatin (Red) and amyloid plaques (Green, stained by 4G8 or 82E1) in cortices of sporadic AD patients. (**f**) Representative images of immunohistochemistry of Aggregatin (arrowheads) and amyloid plaques (stained by the 6E10 antibody) in adjacent sections (denoted by asterisks) of brains of 9 month-old APP/PS1, 17 month-old Tg2576 or 17 month 3xTg mice. (**g**) Representative images of Aggregatin (Red), amyloid plaques (Green, stained by the 6E10 antibody) and DAPI nuclei staining (Blue) in brains of 6 month-old TgCRND8 mice**.** (**h**) Representative images of Aggregatin foci (Red), amyloid plaques (Green, stained by the NU-4 antibody) and DAPI nuclei staining (Blue) in brains of 6 month-old 5xFAD or TgCRND8 mice. (**i**) Representative images of immunohistochemistry of Aggregatin (arrowheads) and amyloid plaques (stained by the 6E10 antibody) in 5XFAD mice at different ages.

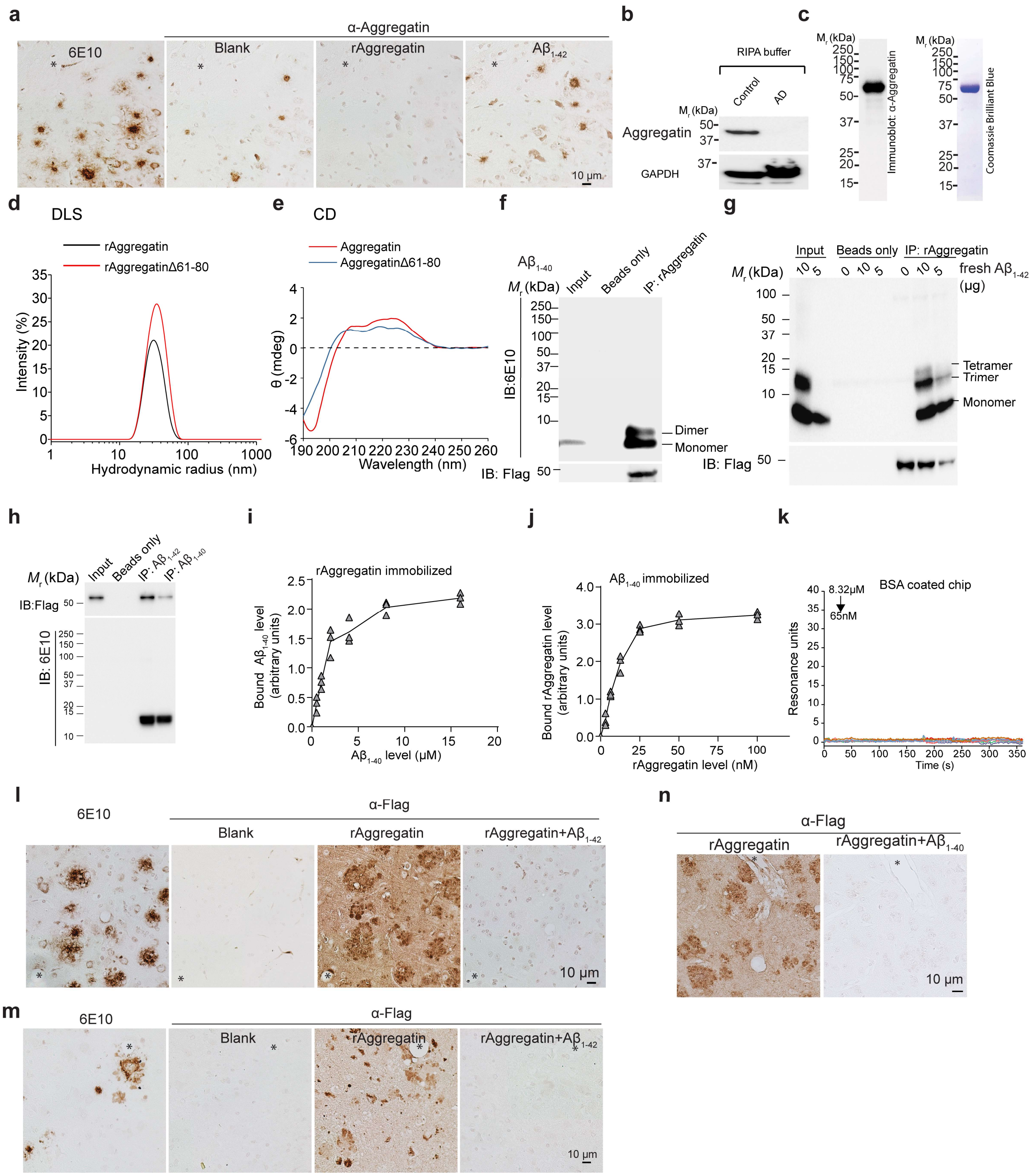

Supplementary Figure 7. The binding of Aggregatin to amyloid plaques or Aβ. (**a**) Representative images of immunohistochemistry of amyloid plaques (6E10 antibody) and Aggregatin in adjacent 6-month old 5xFAD mouse brain sections pre-incubated with or without 100 nM Flag-tagged rAggregatin or 10 μM Aβ$_{1\text{-}42}$. (**b**) Representative immunoblot of Aggregatin in brain extracts from control or AD cortices by RIPA buffer. (**c**) Representative immunoblot (left panel, recognized by the Aggregatin antibody) and Coomassie blue gel staining of 4xFlag-TST tagged rAggregatin. (**d**) Particle size distribution from dynamic light scattering of rAggregatin. Similar ~35 nm peaks were observed in both rAggregatin and rAggregatin Δ61-80 groups. (**e**) Averaged circular dichroism spectra of rAggregatin and rAggregatin Δ61-80 at 1 μM in 10 mM

phosphate buffer (pH8.0). Characteristic far-UV CD spectra for an all-α-helix, an all-β-sheet and a random coil protein. The spectrum for an all-α-helix protein has two negative bands of similar magnitude at 222 and 208 nm, and a positive band at ~ 190 nm. The spectrum for an all β-sheet protein has in general a negative band between 210-220 nm and a positive band between 195-200 nm. The spectrum for a disorderly (random) protein has a negative band of great magnitude at around 200 nm. CD spectra of Aggregatin showed a negative band of great magnitude at around 200 nm, which is characteristic of an intrinsically disordered protein. Further calculation by K2D3 indicates that the content in α-helix and β-sheet was found to be 2.98% and 33.5% in wild-type Aggregatin, respectively, whereas 2.63% and 33.87% was found in Aggregatin Δ61-80. (**f**) Coimmunoprecipitation of purified Flag-tagged rAggregatin and freshly prepared $A\beta_{1-42}$ without pre-aggregated *in vitro* (loaded at different amounts). rAggregatin was immunoprecipitated using streptavidin magnetic beads and immunoblotted using the antibody to Flag. (**g**) Coimmunoprecipitation of purified Flag-tagged rAggregatin and freshly prepared $A\beta_{1-40}$. rAggregatin was also immunoprecipitated using streptavidin magnetic beads and immunoblotted using the antibody to Flag. (**h**) Reverse IP experiment. Strep tagged $A\beta_{1–40}$ or $A\beta_{1–42}$ was purified by Strevdin-avdin beads from HEK293 cells. rAggregatin (3μg/reaction) was immunoprecipitated using $A\beta_{1–40}$ or $A\beta_{1–42}$ bound streptavidin magnetic beads and immunoblotted using the antibody to Flag. Strep tagged $A\beta_{1–40}$ or $A\beta_{1–42}$ was immunoblotted using the 6E10 antibody. (**i**) Measurement of $A\beta_{1-40}$ levels bound to immobilized rAggregatin (normalized to maximal rAggregatin and $A\beta_{1-40}$ binding). n = 3 biologically independent samples. (**j**) Measurement of rAggregatin levels bound to immobilized $A\beta_{1-40}$ (normalized to maximal rAggregatin and $A\beta_{1-40}$ binding). n = 3 biologically independent samples. (**k**) Bio-layer interferometry measurement of the binding kinetics of monomeric $A\beta_{1-42}$ to immobilized BSA (as negative controls). Curves are corresponded to $A\beta_{1-42}$ at 8320, 4160, 2080,1040, 520, 260, 130 and 65 nM from the top to bottom. (**l**) Representative images of immunohistochemistry of amyloid plaques (6E10 antibody) and rAggregatin (Flag antibody) in adjacent 6-month old 5xFAD mouse brain sections pre-incubated with or without 100 nM Flag-tagged rAggregatin and 10 μM $A\beta_{1-42}$. Asterisks denote landmarks in adjacent sections. (**m**) Representative images of immunohistochemistry of amyloid plaques (6E10 antibody) and rAggregatin (Flag antibody) in adjacent brain sections of sporadic AD patients pre-incubated with or without 100 nM Flag-tagged rAggregatin and 10 μM $A\beta_{1-42}$. (**n**) Representative images of immunohistochemistry of rAggregatin (Flag antibody) in adjacent 6-month old 5xFAD mouse brain sections pre-incubated with or without 100 nM Flag-tagged rAggregatin and 50 μM $A\beta_{1-40}$. Source data are provided as a Source Data file (Source Data for Statistics and Blots).

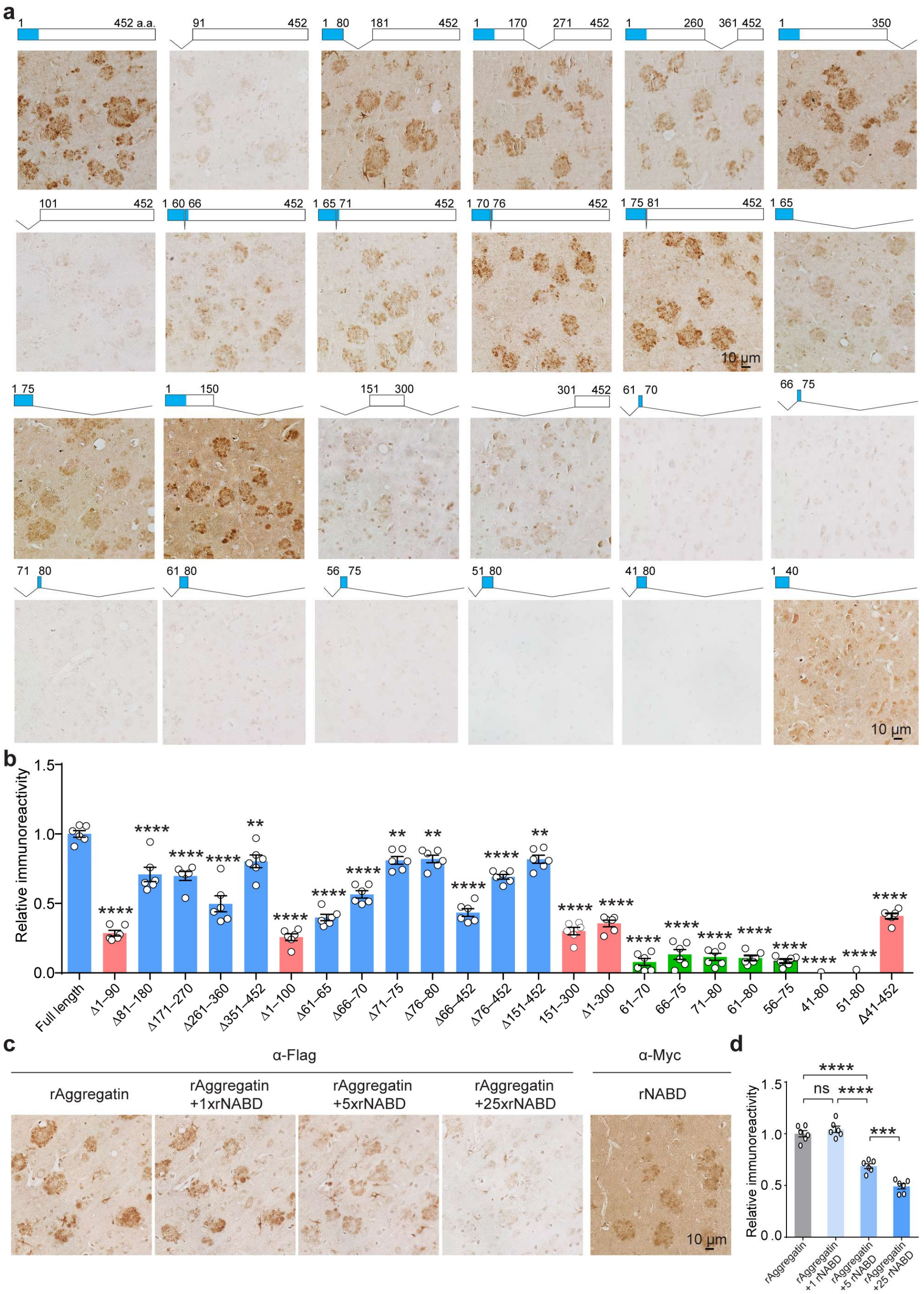

Supplementary Figure 8. Identification of the binding motif of Aggregatin to amyloid plaques. (**a** and **b**) Representative immunohistochemistry (a) and quantification (b) of rAggregatin immunoreactivity (Flag antibody) in 5xFAD mouse brain after incubation with 100 nM indicated rAggregatin deletion mutants (*n* = 6 biologically independent samples in each group). (**c** and **d**) Representative immunohistochemistry (c) and quantification (d) of rAggregatin immunoreactivity (Flag antibody) in 5xFAD mouse brain co-incubated with 100 nM Flag-tagged rAggregatin and different ratios of Myc-tagged rNABD (i.e, 100 nM, 500 nM and 2,500 nM, *n* = 6 biologically independent samples in each group). Source data are provided as a Source Data file (Source Data for Statistics and Blots). Data are means ± s.e.m. One-way analysis of variance (ANOVA) followed by Tukey's multiple comparison test. ***P*< 0.01, ****P*< 0.001, *****P*< 0.0001. ns, non-significant.

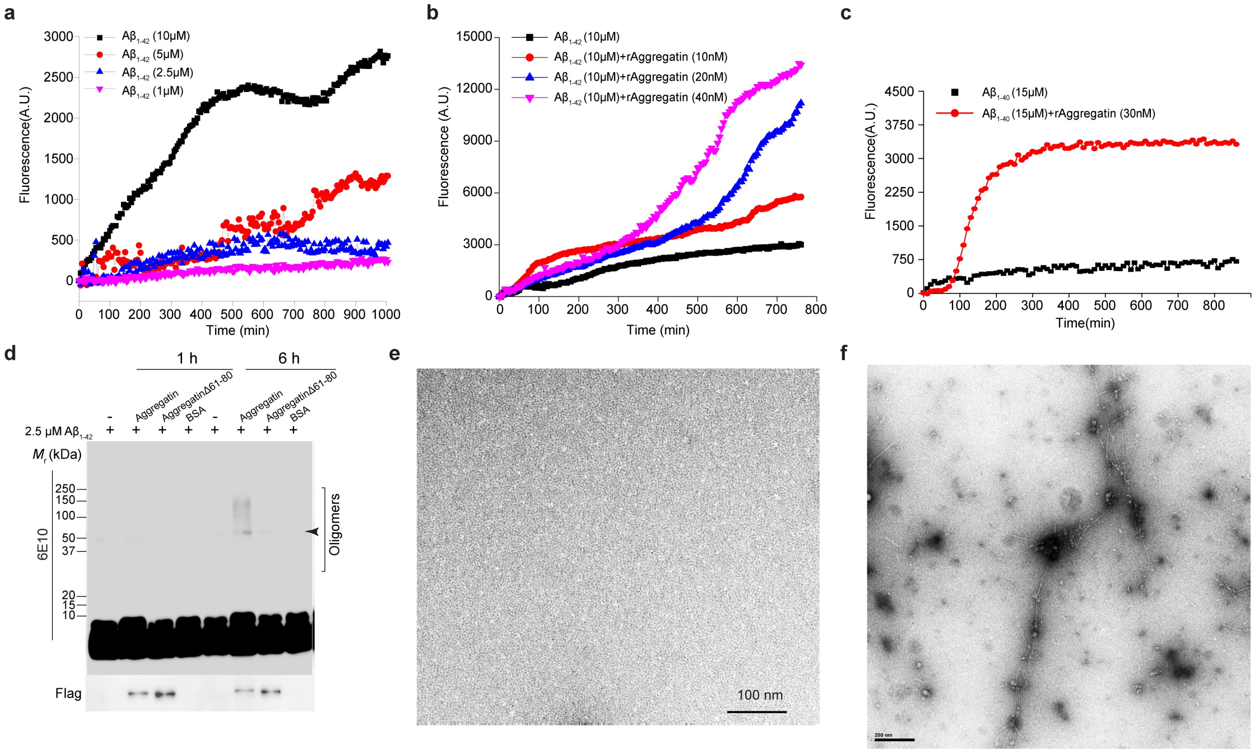

Supplementary Figure 9. Aggregatin enhances Aβ aggregation *in vitro*. (**a**) ThT-based assay measuring aggregation kinetics of various concentrations of $A\beta_{1-42}$ indicating that the low concentration of $A\beta_{1-42}$ at 2.5 μM alone is not sufficient to induce ThT fluorescent increase *in vitro* (*n* = 5 biologically independent samples in each time points). (**b**) ThT-based assay measuring aggregation kinetics of the high concentration of $A\beta_{1-42}$ (10 μM, which alone causes greatly increased ThT fluorescent *in vitro*, as shown in A) in the presence of various concentrations of rAggregatin (*n* = 5 biologically independent samples in each time points). (**c**) ThT-based assay measuring aggregation kinetics of $A\beta_{1-40}$ (15 μM) in the presence of 30 nM rAggregatin (*n* = 5 biologically independent samples in each time points). (**d**) Representative light exposure of $A\beta_{1-42}$ oligomers recognized by 6E10 in the 30 nM rAggregatin and 2.5 μM $A\beta_{1-42}$ mixture collected after 6-hour co-incubation. (**e** and **f**) Representative large filed images of negative staining electron microscopy of rAggregatin (no detectable aggregates) or $A\beta_{1-42}$ (2.5 μM) aggregates 4 weeks after co-incubation with rAggregatin (30 nM). Source data are provided as a Source Data file (Source Data for Statistics and Blots).

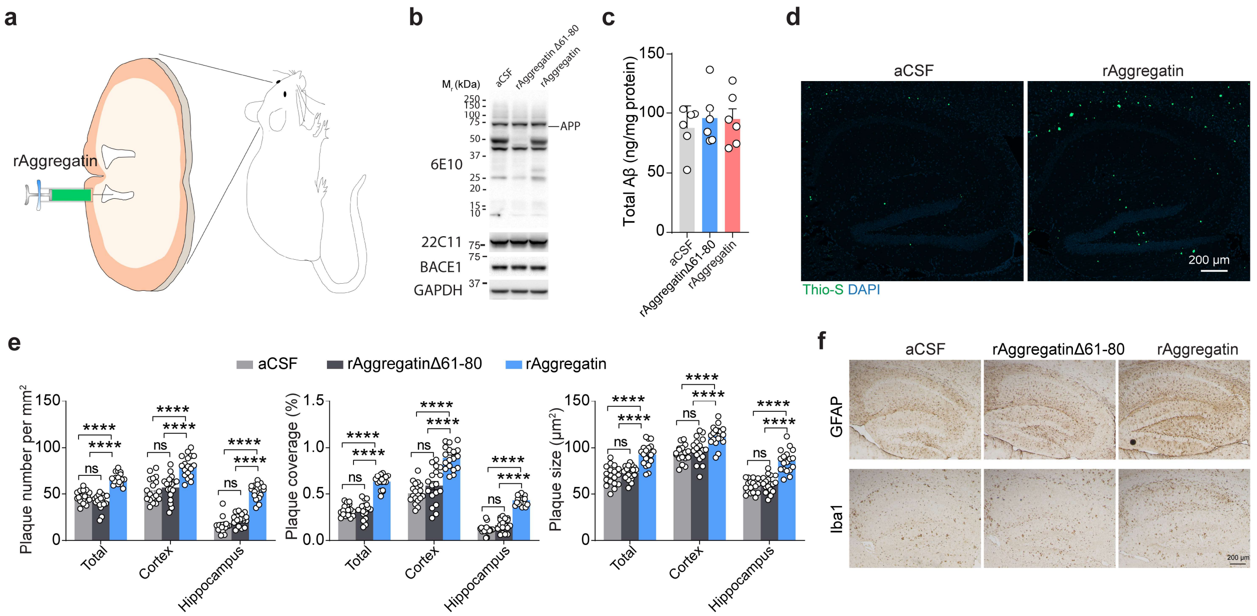

Supplementary Figure 10. rAggregatin ICV infusion exacerbates amyloid deposits and related neuroinflammation in 5XFAD mice. (**a**) Schematic of rAggregatin ICV infusion. (**b**) Representative immunoblot of human APP, total APP (human and mouse APP) and BACE1 in brains of 5 month-old mice with ICV infusion of Flag-tagged rAggregatinΔ61-80 or rAggregatin in right half brain at 4 month-old for 4 weeks. (**c**) Measurements of total Aβ levels in brains of mice with ICV infusion (n = 6 biologically independent samples in each group). (**d** and **e**) Representative images (d) and quantification (e) of plaque density, load and size by staining a broader range of amyloid plaques using fibrillar dense-core amyloid plaques by Thio-S (d and e) in the total brain (Total), cortex or hippocampus of 5-month old 5xFAD mice with ICV infusion of Flag-tagged rAggregatinΔ61-80 or rAggregatin for 4 weeks (n = 18 biologically independent samples in each group). (**f**) Representative images of astrogliosis (stained by GFAP) and microgliosis (stained by Iba1) in hippocampus of 5-month old 5xFAD mice infused with Flag-tagged rAggregatinΔ61-80 or rAggregatin for 4 weeks. Source data are provided as a Source Data file (Source Data for Statistics and Blots). Data are means ± s.e.m. All experiments were independently performed at least three times. Student's t-test or One-way analysis of variance (ANOVA) followed by Tukey's multiple comparison test. ****$P < 0.0001$. ns, non-significant.

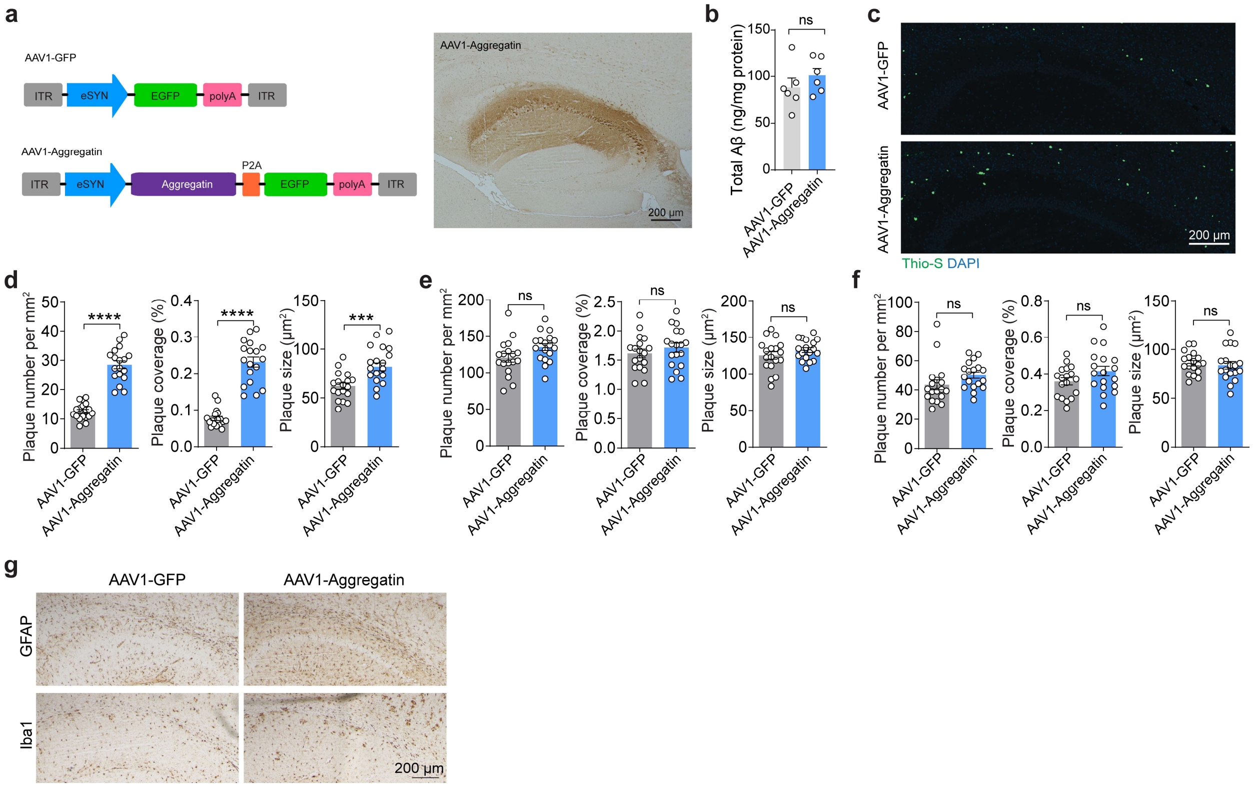

Supplementary Figure 11. Overexpression of Aggregatin in neurons enhances amyloid deposition and associated neuroinflammation. (**a**) Schematic diagram of AAV1-Aggregatin and AAV1-GFP. ITR, inverted terminal repeats; eSYN, a hybrid promoter consisting of cytomegalovirus enhancer and human Synapsin I promoter; P2A, porcine teschovirus 2A peptide sequence. P2A autocleavage generates Aggregatin separately from GFP. Right panel shows the representative immunohistochemistry of GFP in 5 month-old 5XFAD mice injected with 2 μl AAV1-Aggregatin into the hippocampal CA1 at 1.5 month-old. (**b**) Measurements of total Aβ levels in isolated hippocampus of 5 month-old 5XFAD mice injected with 2 μl AAV1-Aggregatin into the hippocampal CA1 at 1.5 month-old. (n = 6 biologically independent samples in each group). (**c** and **d**) Representative images (c) and quantification (d) of fibrillar dense-core amyloid plaques by Thio-S in the hippocampus of 5 month-old 5XFAD mice injected with AAV1-GFP or AAV1-Aggregatin at 1.5 month-old (n = 18 biologically independent samples in each group). (**e** and **f**) Quantification of amyloid plaques stained by NU-4 (e) or Thio-S (f) in the brain stem not infected with AAV1 (GFP-negative, n = 18 biologically independent samples in each group). (**g**) Representative images of astrogliosis (stained by GFAP) and microgliosis (stained by Iba1) in the hippocampus of 5 month-old 5XFAD mice injected with AAV1-GFP or AAV1-Aggregatin at 1.5 month-old (n = 18 biologically independent samples in each group). Source data are provided as a Source Data file (Source Data for Statistics and Blots). Data are means ± s.e.m. Student's t-test or One-way analysis of variance (ANOVA) followed by Tukey's multiple comparison test. ***$P < 0.001$ and ****$P < 0.0001$. ns, non-significant.

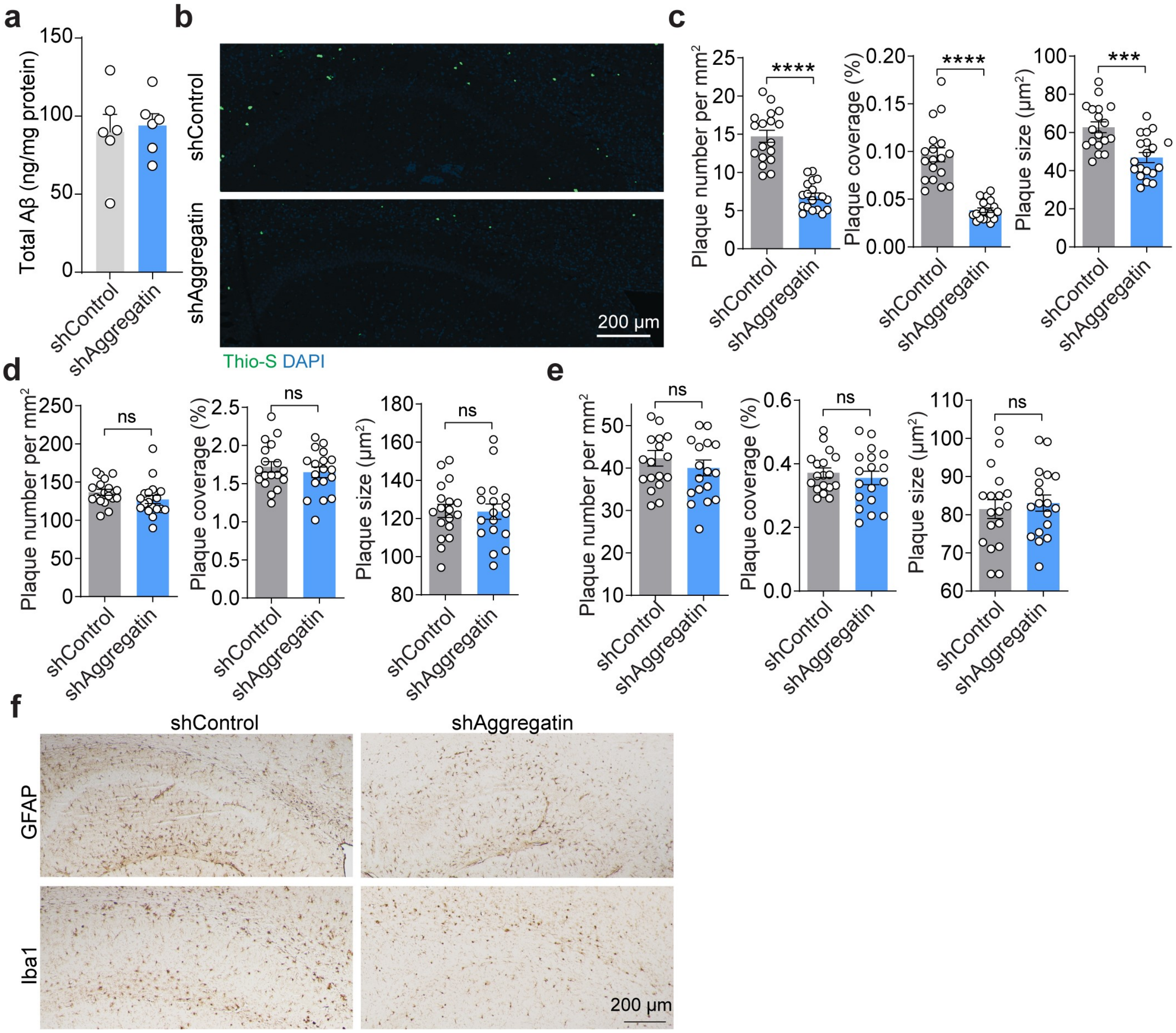

Supplementary Figure 12. Aggregatin deficiency inhibits amyloid deposition and associated neuroinflammation. (**a**) Measurements of total Aβ levels in isolated hippocampus of 5 month-old 5XFAD mice injected with 2 μl AAV1-shAggregatin or AAV1-shControl into the hippocampal CA1 at 1.5 month-old. (*n* = 6 biologically independent samples in each group). (**b** and **c**) Representative images (b) and quantification (c) of fibrillar dense-core amyloid plaques by Thio-S in the hippocampus of 5 month-old 5XFAD mice injected with AAV1-shAggregatin or AAV1-shControl at 1.5 month-old (*n* = 18 biologically independent samples in each group). (**d** and **e**) Quantification of amyloid plaques stained by NU-4 (d) or Thio-S (e) in the brain stem not infected with AAV1 (GFP-negative, *n* = 18 biologically independent samples in each group). (**f**) Representative images of astrogliosis (stained by GFAP) and microgliosis (stained by Iba1) in the hippocampus of 5 month-old 5XFAD mice injected with AAV1-shControl or AAV1-shAggregatin at 1.5 month-old (n = 18 biologically independent samples in each group). Source data are provided as a Source Data file (Source Data for Statistics and Blots). Data are means ± s.e.m. Student's *t*-test or One-way analysis of variance (ANOVA) followed by Tukey's multiple comparison test. ***P < 0.001 and ****P < 0.0001. ns, non-significant.

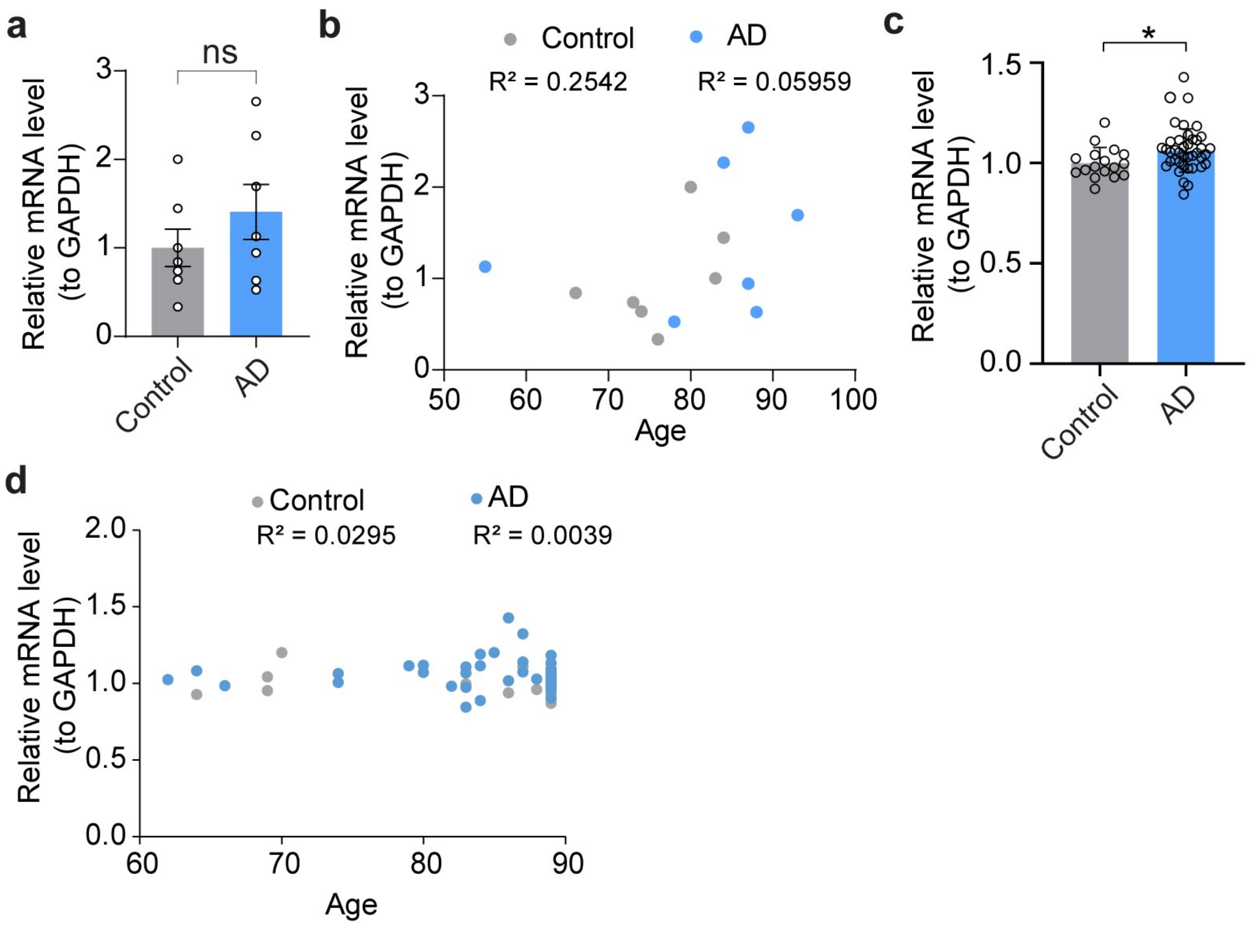

Supplementary Figure 13. The expression of Aggregatin mRNA in AD. (**a** and **b**) Quantification of Aggregatin mRNA levels (a) and correlation study of Aggregatin mRNA with age (b) in AD cortices (*n* = 7 biologically independent samples) compared with age-matched controls (*n* = 7 biologically independent samples) (using samples collected in our laboratory and measured by RT-PCR). All experiments were independently performed at least three times. (**c** and **d**) Quantification of Aggregatin mRNA levels (c) and correlation study of Aggregatin mRNA with age (d) in AD prefrontal cortices (*n* = 39 biologically independent samples) compared to age-matched prefrontal cortices (*n* =17 biologically independent samples) based on published MSBB microarray data[1]. Source data are provided as a Source Data file (Source Data for Statistics and Blots). For "**a**", *P*-value of logistic regression association analysis between AD diagnosis and Aggregation mRNA level is 0.532 (Odds Ratio = 1.86). The analysis includes age and gender as covariates. For "**c**", *P*-value of logistic regression association analysis between AD diagnosis and Aggregation mRNA level is 0.0422 (Odds Ratio = 2.21). The analysis also includes age and gender as covariates. Data are means ± s.e.m. Student's t-test. *$P < 0.05$, ns, non-significant.

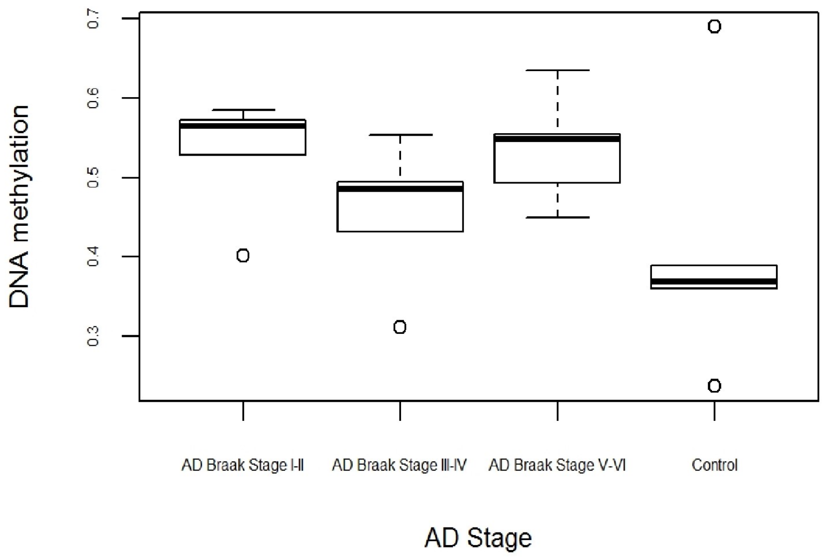

Supplementary Figure 14. Boxplot of DNA methylation level of cg01335367 on *FAM222A* in hippocampus across AD Braak stages. One-way ANOVA test difference between methylation levels of control and three AD Braak stage groups P-value is 0.127. Source data are provided as a Source Data file (Source Data for Statistics and Blots).

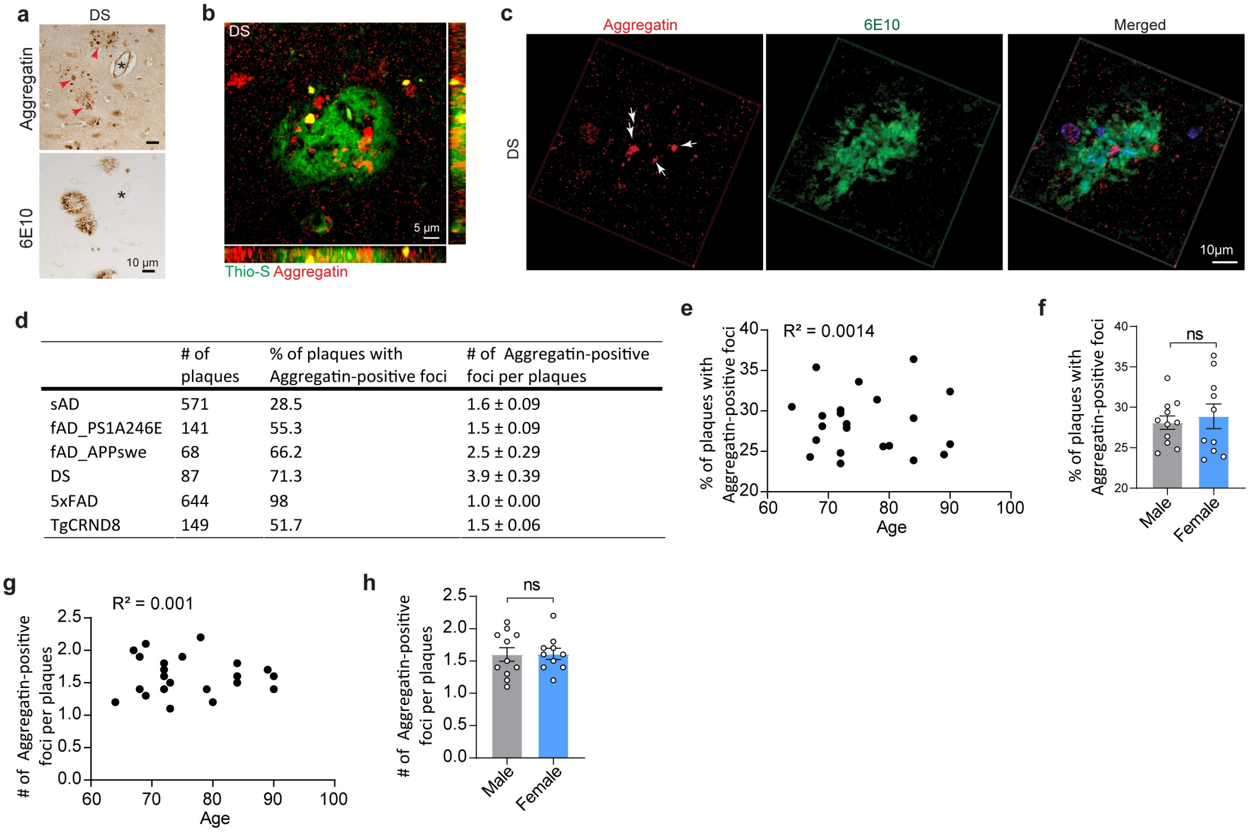

	# of plaques	% of plaques with Aggregatin-positive foci	# of Aggregatin-positive foci per plaques
sAD	571	28.5	1.6 ± 0.09
fAD_PS1A246E	141	55.3	1.5 ± 0.09
fAD_APPswe	68	66.2	2.5 ± 0.29
DS	87	71.3	3.9 ± 0.39
5xFAD	644	98	1.0 ± 0.00
TgCRND8	149	51.7	1.5 ± 0.06

Supplementary Figure 15. Aggregatin accumulates within the center of amyloid plaques in DS. (**a**) Representative images of immunohistochemistry of Aggregatin (arrowheads) and amyloid plaques (stained by the 6E10 antibody) in adjacent sections (denoted by asterisks) of cortices of sporadic DS patients. (**b** and **c**) Representative images of Aggregatin foci (Red), amyloid plaques (Green, stained by Thio-S (b) or 6E10 antibody (c), and DAPI nuclei staining (Blue) in cortices of sporadic DS patients. (**d**) Quantification of the percentage of plaques containing Aggregatin foci and the number of Aggregatin foci in each plaque in sAD, fAD or DS patients, 6-month old 5xFAD mice and 6-month old TgCRND8 mice. (**e–h**) Correlation study of the percentage of plaques containing Aggregatin foci (e, f) and the number of Aggregatin foci (g, h) with age (e and g) or gender (f and h) in each plaque in sAD patients. ns, non-significant. Source data are provided as a Source Data file (Source Data for Statistics and Blots).

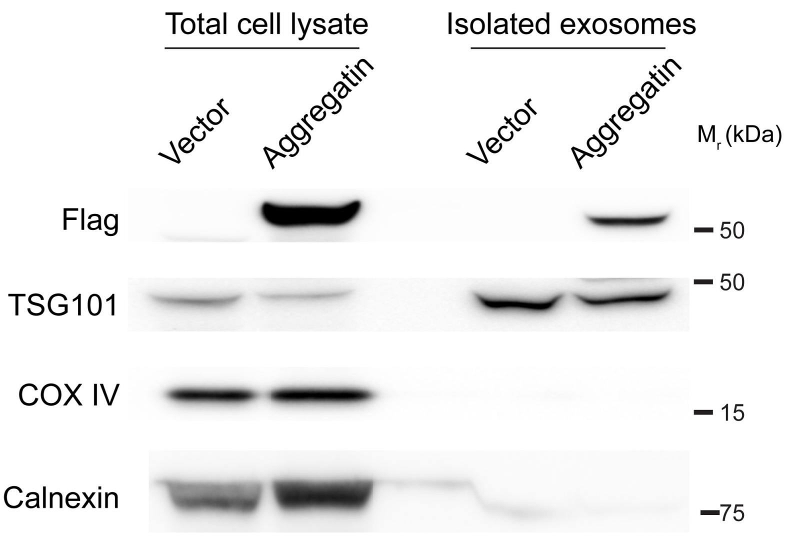

Supplementary Figure 16. The presence of Aggregatin in exosomes. Representative Immunoblot of Aggregatin in exosomes isolated from 293 cells expressing Flag tagged Aggregatin. TSG101 was used as the exosome marker. COX IV and Calnexin, markers for mitochondria and ER respectively, were used as negative markers for exosome. Source data are provided as a Source Data file (Source Data for Statistics and Blots).

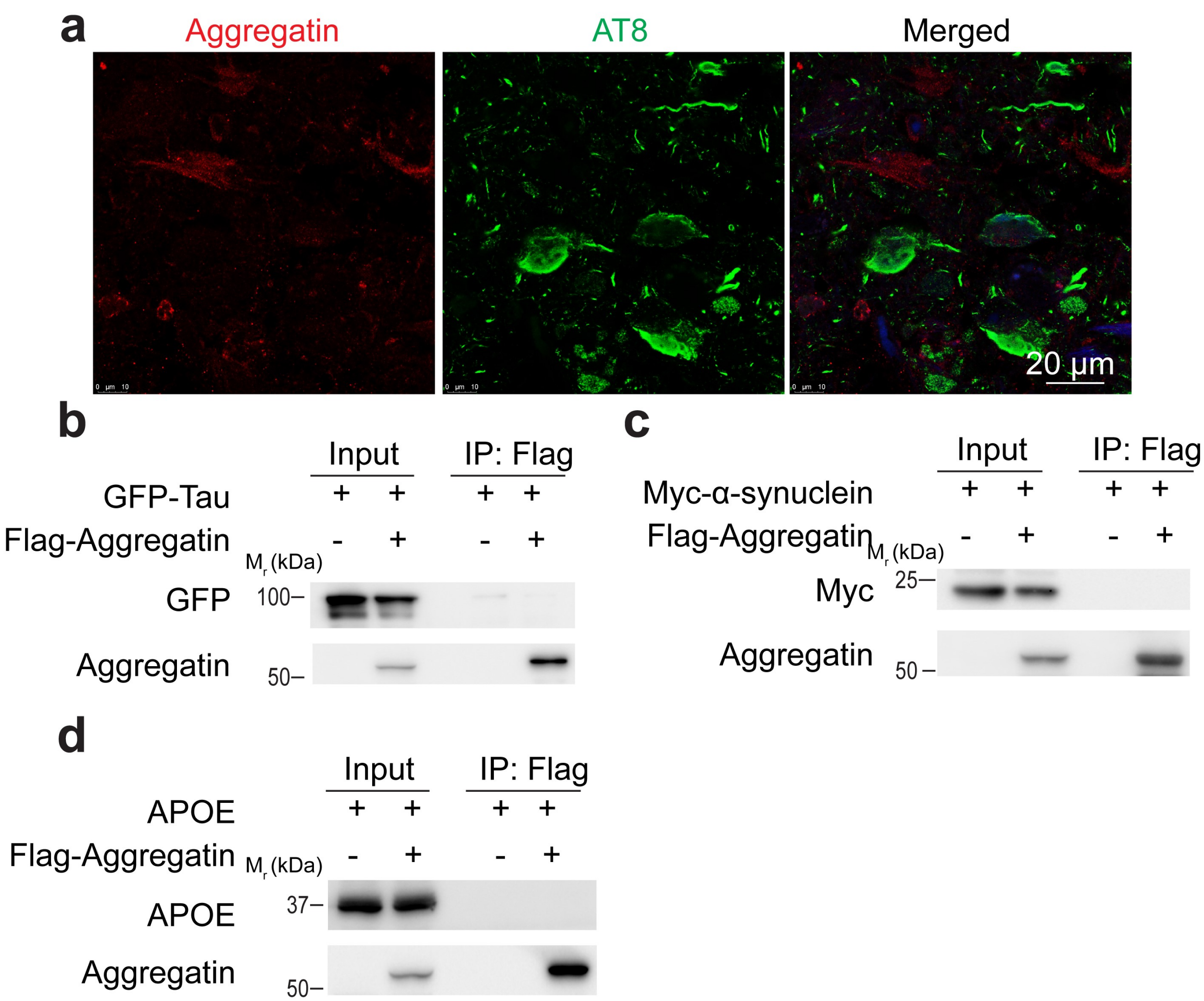

Supplementary Figure 17. Aggregatin does not interact with Tau, α-synuclein or APOE. (**a**) Representative images of Aggregatin (Red) and neurofibrillary tangles (Green, stained by the AT8 antibody), in cortices of sAD (blue: DAPI staining). (**b**). Co-IP of Flag-tagged Aggregatin with GFP-tagged Tau in HEK293 cells. Aggregatin was precipitated using streptavidin magnetic beads followed by immunoblot using the antibody to Aggregatin or GFP. (**c**) Co-IP of Flag-tagged Aggregatin with Myc-tagged α-synuclein in HEK293 cells. Aggregatin was precipitated using streptavidin magnetic beads followed by immunoblot using the antibody to Aggregatin or Myc. (**d**) Co-IP of Flag-tagged Aggregatin with APOE in HEK293 cells overexpressing human APOE. Aggregatin was precipitated using streptavidin magnetic beads followed by immunoblot using the antibody to Aggregatin or APOE. Source data are provided as a Source Data file (Source Data for Statistics and Blots).

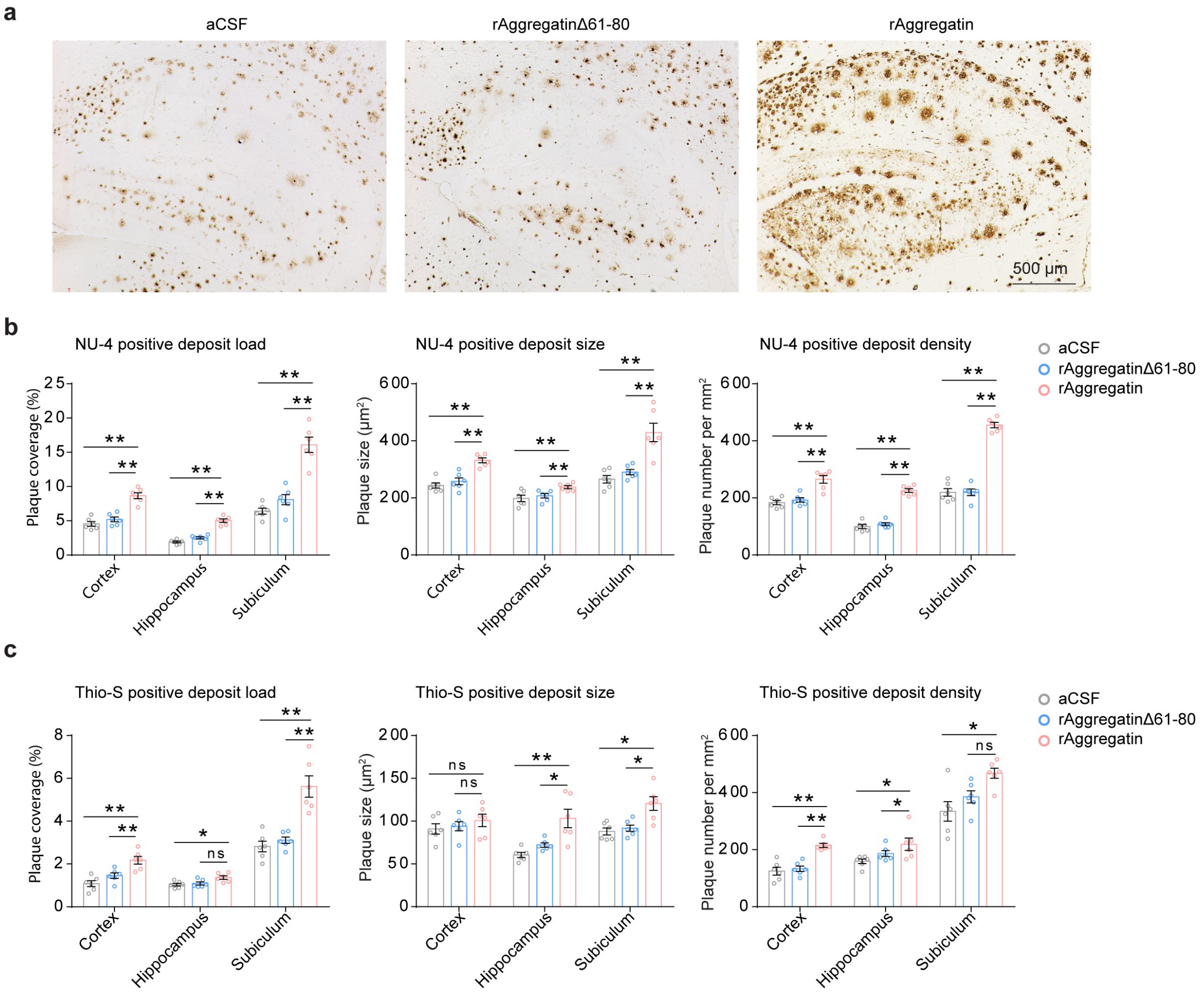

Supplementary Figure 18. ICV of rAggregatin accelerates amyloid deposition in aged 5XFAD mice. (**a–c**) Representative images (a) and quantification (b and c) of plaque load and size by staining a broader range of amyloid plaques using NU-4 (b) or fibrillar dense-core amyloid plaques by Thio-S (c) in the total brain (Total), cortex or hippocampus of 12-month old 5xFAD mice with ICV infusion of Flag-tagged rAggregatinΔ61-80 or rAggregatin for 4 weeks (*n* = 6 biologically independent samples in each group). Source data are provided as a Source Data file (Source Data for Statistics and Blots). Data are means ± s.e.m.

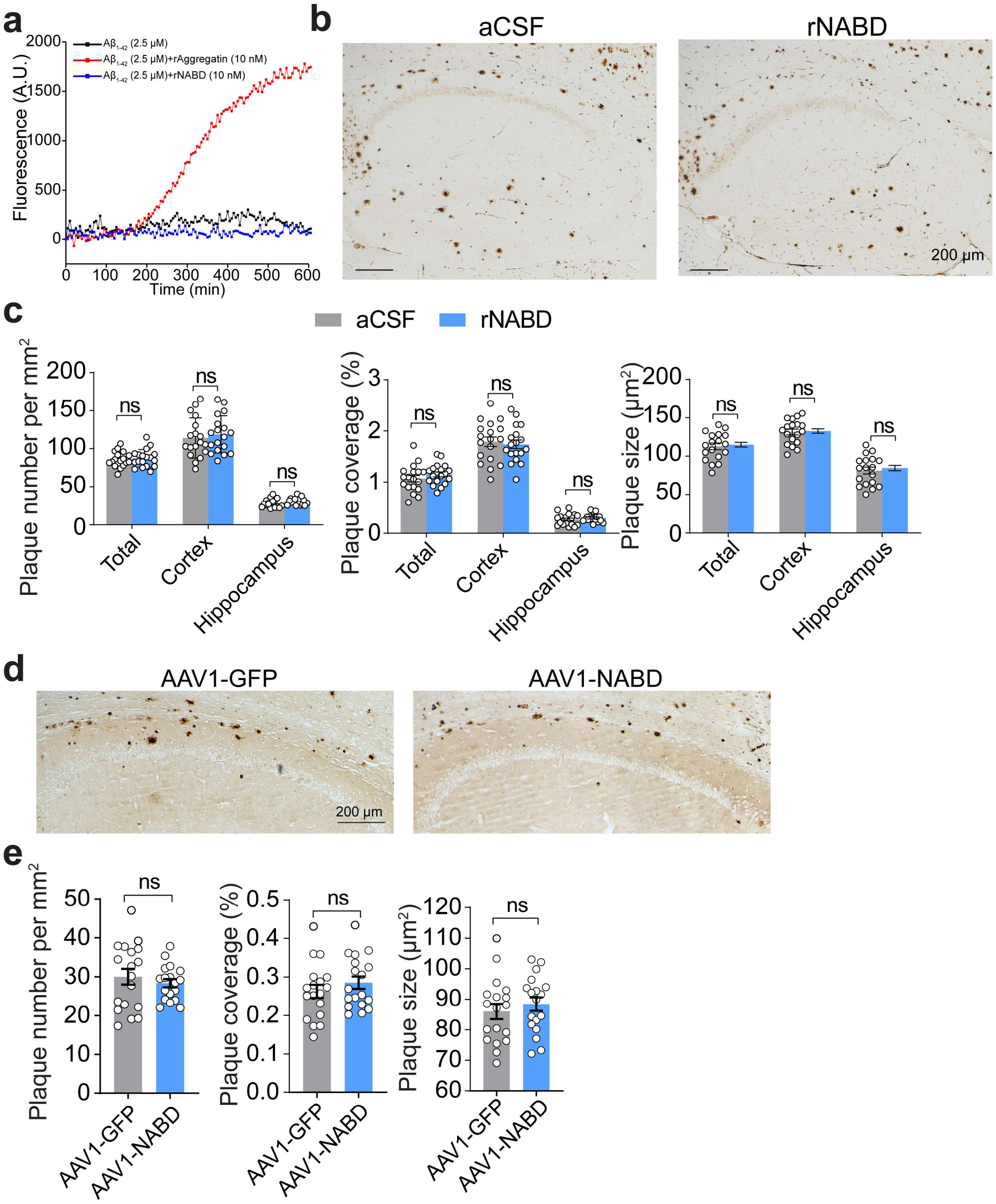

Supplementary Figure 19. rNABD (rAggregatin1-80 or rAggregatinΔ81-452) has no effect on Aβ aggregation or amyloid deposits. (**a**). ThT-based assay measuring aggregation kinetics of $Aβ_{1-42}$ in the presence of rAggregatin or rNABD indicating that rNABD is not sufficient to induce ThT fluorescent increase *in vitro* (*n* = 5 biologically independent samples in each time points). (**b** and **c**) Representative images (b) and quantification (c) of plaque density, load, and size by staining a broader range of amyloid plaques using NU-4 in the total brain (Total), cortex or hippocampus of 5-month old 5xFAD mice with ICV infusion of Flag-tagged rNABD (i.e., rAggregatinΔ81-452) for 4 weeks (n = 18 biologically independent samples in each group). (**d** and **e**) Representative images (d) and quantification (e) of the density, load, and size of plaques stained by NU-4 in the hippocampus CA1 of 5 month-old 5XFAD mice injected with AAV1-GFP or AAV1-NABD (i.e., AggregatinΔ81-452) at 1.5 month-old (*n* = 18 biologically independent samples in each group). Source data are provided as a Source Data file (Source Data for Statistics and Blots). Data are means ± s.e.m. All experiments were independently performed at least three times. Student's *t*-test. ns, non-significant.

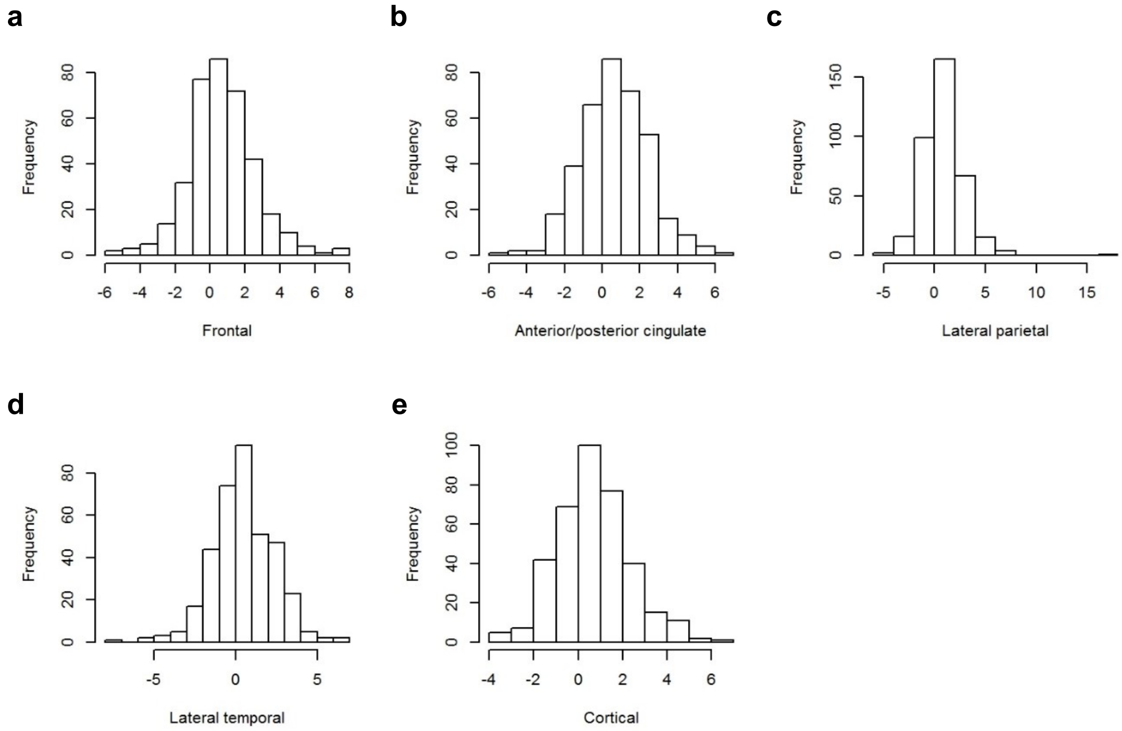

Supplementary Figure 20. Distribution of annual percent change of SUVR means for frontal (**a**), anterior/posterior cingulate (**b**), lateral parietal (**c**), lateral temporal (**d**) and composite cortical (**e**). Source data are provided as a Source Data file (Source Data for Statistics and Blots).

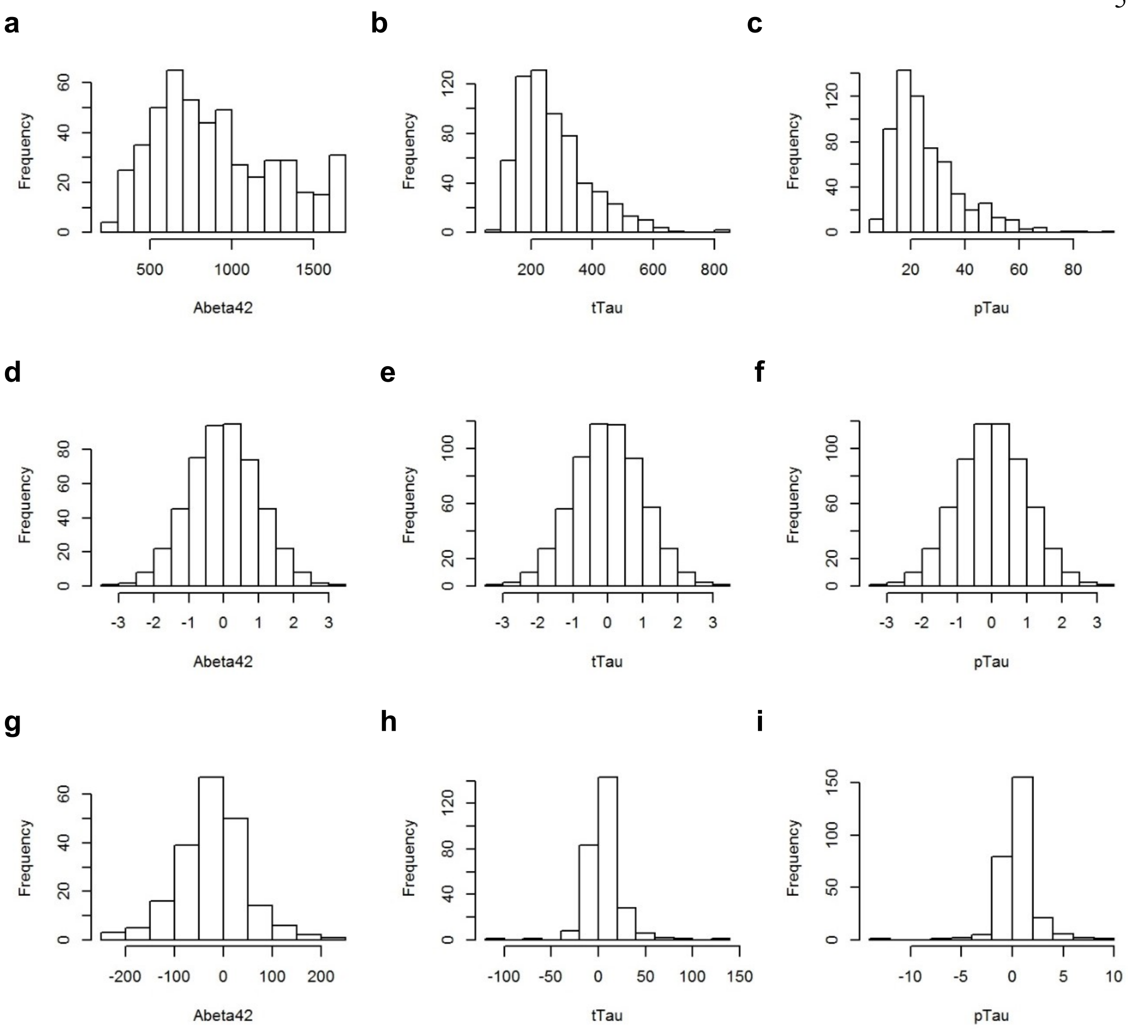

Supplementary Figure 21. Distribution of ADNI CSF biomarkers level. **(a–c)** Baseline CSF raw level of Aβ42 (a), tTau (b) and pTau (c); **(d–f)** Rank-normal transformed baseline CSF Aβ42 (d), tTau (e) and pTau (f); **(g–i)** Annual percent changes of CSF Aβ42 (g), tTau (h) and pTau (i). Source data are provided as a Source Data file (Source Data for Statistics and Blots).

Loci identified with P-value < 1E-7 in the within module CPASSOC analysis						
**Chr**	**SNP**	**Gene**	**P-value**	**Method**	**Brain Regions**	**Color**
2	rs4670544	*CRIM1*	3.24E-08	CPASSOC (S_{Hom})	*Left Pallidum; Left Putamen; Right/Left Thalamus Proper; Anterior Limb of Internal Capsule Right; Posterior Limb of Internal Capsule Right/Left; Corpus Callosum*	
13	rs729105	*SAP18/SKA3*	5.07E-08	CPASSOC (S_{Het})		
19	rs2075650	*TOMM40*	2.60E-08	CPASSOC (S_{Het})	*Right/Left Hippocampus; Right Basal Forebrain; Left Entorhinal Area; Right Planum Polare*	
19	rs429358	*APOE*	1.80E-12	CPASSOC (S_{Het})		
19	rs439401	*APOE/APOC1*	8.80E-08	CPASSOC (S_{Het})		
12	rs117028417	*FAM222A*	1.95E-08	CPASSOC (S_{Het})		
1	rs145582754	*BC042538*	4.57E-09	CPASSOC (S_{Het})	*Right Putamen; Left Lingual Gyrus; Left Planum Polare; right Temporal Pole*	
9	rs744216	*SMARCA2/ VLDLR*	2.87E-08	CPASSOC (S_{Het})	*Left Frontal Pole; Right Middle Cingulate Gyrus; Right Middle Occipital Gyrus; Right Occipital Pole; Right Opercular Part of Inferior Frontal Gyrus*	
10	rs11197596	*GFRA1*	9.60E-08	CPASSOC (S_{Hom})	*Right Anterior Cingulate Gyrus; Left Frontal Operculum; Left Superior Frontal Gyrus Medial Segment; Left Opercular Part of Inferior Frontal Gyrus; Right Para-hippocampal Gyrus*	
20	rs13044355	*SLC24A3*	2.18E-08	CPASSOC (S_{Het})		
1	rs1325440	*SLC6A9*	9.47E-08	CPASSOC (S_{Het})	*Temporal Lobe White Matter Right/Left; Left Parietal Operculum*	
3	rs6549225	*FRMD4B*	1.83E-08	CPASSOC (S_{Het})	*Brain Stem; Right Pallidum; Frontal Lobe WM Right/Left; Occipital Lobe WM Right/Left; Parietal Lobe WM Right/Left; Fornix Right; Right Entorhinal Area; Left Postcentral Gyrus Medial Segment; Left Occipital Fusiform Gyrus; Left Para-hippocampal Gyrus; Right/Left PIns Posterior insula*	
12	rs2657575	*TMEM117*	8.56E-08	CPASSOC (S_{Het})		
10	rs76434005	*MBL2*	2.75E-08	CPASSOC (S_{Het})	*Left Cuneus; Left Gyrus Rectus; Left Lateral Orbital Gurus; Left Postrior Orbital Gurus; Left Superior Occipital Gyrus*	
5	rs12658840	*ATP10B/ GABRB2*	9.51E-08	CPASSOC (S_{Het})	*Right/Left AIns Anterior Insula; Right Medial Orbital Gyrus*	

Supplementary Table 1. SNPs with P-value < 1E-7 in the within module CPASSOC analysis.

Association of rs117028417 with brain region volumes in ADNI-1								
**Consortium**	**Sample Size**	**Effect Allele**	**Non-effect Allele**	**Effect Allele Frequency**	**Effect Size**	**SE**	**P-value**	**Phenotype**
ADNI-1	175 AD; 366 MCI; 213 Controls	A	G	0.044	111.2	22.6	8.12×10^{-7}	Left Hippocampus
		A	G	0.044	115.0	23.5	9.03×10^{-7}	Right Hippocampus
		A	G	0.044	34.0	8.8	1.01×10^{-4}	Right Basal Forebrain
		A	G	0.044	84.2	20.9	5.75×10^{-5}	Left Entorhinal Area
		A	G	0.044	43.9	11.0	6.22×10^{-4}	Right Planum Polare
		A	G	NA	NA	N/A	1.95×10^{-8}	CPASSOC combine results of above 5 ROIs

Association of rs117028417 with mean hippocampus volume in in 30,717 subjects from ENIGMA consortium[a]								
**Consortium**	**Sample Size**	**Effect Allele**	**Non-effect Allele**	**Effect Allele Frequency**	**Effect Size**	**SE**	**P-value**	**Phenotype**
ENIGMA	12,778	A	G	0.047	38.1	14.4	8.29×10^{-3}	Mean Hippocampus

Supplementary Table 2. a: ENIGMA consortium is from a published large-scale subcortical regions and the intracranial volume GWAS[2]. The brain volume measures were obtained from structural MRI data and processed using software FIRST[3]. The mean hippocampus volume is average of left and right hippocampus. 21% of the participants in this study were diagnosed with anxiety, AD, attention-deficit/hyperactive disorder, bipolar disorder, epilepsy, major depression disorder or schizophrenia[2].

Brain region	Genetic Variant	Effect Allele	Non-effect Allele	Effect Allele Frequency	Effect Size	SE	*P* value
Frontal		A	G	0.032	-0.154	0.108	0.152
Anterior/posterior cingulate		A	G	0.032	-0.294	0.109	0.007
Lateral parietal	rs117028417	A	G	0.032	-0.182	0.078	0.021
Lateral temporal		A	G	0.032	-0.073	0.058	0.208
Composite cortical		A	G	0.032	-0.488	0.208	0.019
Frontal		ε4	ε2 or ε3	0.244	0.48	0.16	0.003
Anterior/posterior cingulate		ε4	ε2 or ε3	0.244	0.36	0.15	0.014
Lateral parietal	APOE ε4	ε4	ε2 or ε3	0.244	0.53	0.17	0.002
Lateral temporal		ε4	ε2 or ε3	0.244	0.49	0.16	0.002
Composite cortical		ε4	ε2 or ε3	0.244	0.46	0.13	0.0007

Supplementary Table 3. Single SNP association test of rs117028417 with longitudinal changes in AV-45 PET SUVR measures.

SNP	Position (GRCh37)	Allele1	Allele2	Minor Allele Frequency	Gene	Annotation
12:110205831_G/A	110205831	G	A	0.00481	*FAM222A*	Missense
12:110206090_T/C	110206090	T	C	0.00122	*FAM222A*	Missense
12:110206245_A/G	110206245	A	G	0.00189	*FAM222A*	Missense
12:110206588_G/A	110206588	G	A	0.00129	*FAM222A*	Missense
12:110205831_G/A	110205831	G	A	0.00248	*FAM222A*	Missense
12:110206090_T/C	110206090	T	C	0.00062	*FAM222A*	Missense
12:110206147_T/C	110206147	T	C	0.00062	*FAM222A*	Missense
12:110206245_A/G	110206245	A	G	0.00289	*FAM222A*	Missense
12:110221524_T/C	110221524	T	C	0.01182	*TRPV4*	Missense
12:110221544_C/T	110221544	C	T	0.00310	*TRPV4*	Missense
12:110224603_T/C	110224603	T	C	0.00371	*TRPV4*	Missense
12:110230568_C/G	110230568	C	G	0.00062	*TRPV4*	Missense
12:110230597_T/C	110230597	T	C	0.01052	*TRPV4*	Missense
12:110232247_A/G	110232247	A	G	0.00062	*TRPV4*	Missense
12:110236615_A/G	110236615	A	G	0.00062	*TRPV4*	Missense
12:110236721_T/C	110236721	T	C	0.00310	*TRPV4*	Splice region
12:110236722_A/G	110236722	A	G	0.00186	*TRPV4*	Splice region
12:110238507_C/G	110238507	C	G	0.00062	*TRPV4*	Missense
12:110246277_A/G	110246277	A	G	0.00124	*TRPV4*	Splice region
12:110252521_G/A	110252521	G	A	0.00433	*TRPV4*	Splice donor
12:110252547_A/G	110252547	A	G	0.02908	*TRPV4*	Missense
12:110252574_G/C	110252574	G	C	0.00062	*TRPV4*	Missense
12:110270116_A/G	110270116	A	G	0.00062	*TRPV4*	Splice region

Supplementary Table 4. Coding variants on *FAM222A* and *TRPV4* in ADNI WGS data.

Gene	Brain region	Burden P-value	SKAT P-value
FAM22A	Frontal	0.063	0.122
	Anterior/posterior cingulate	0.002	0.007
	Lateral parietal	0.004	0.035
	Lateral temporal	0.272	0.421
	Composite cortical	0.039	0.089
TRPV4	Frontal	0.233	0.431
	Anterior/posterior cingulate	0.451	0.287
	Lateral parietal	0.920	0.901
	Lateral temporal	0.178	0.235
	Composite cortical	0.366	0.276

Supplementary Table 5. Gene-based association tests of *FAM222A* and *TRPV4* with longitudinal changes in AV-45 PET SUVR measures.

Genetic Variant	Biomarker	Biomarker Measure	N	Effect Allele	Non-effect Allele	Effect Allele Frequency	Effect (SE)	P-value
rs117028417	Aβ42	Baseline level	494	A	G	0.031	0.22 (0.18)	0.24
	tTau		617	A	G	0.026	-0.11 (0.17)	0.51
	pTau		616	A	G	0.026	-0.12 (0.19)	0.53
	Aβ42	Annual change	203	A	G	0.034	4.99 (2.26)	0.27
	tTau		274	A	G	0.033	-3.61 (1.57)	0.02
	pTau		273	A	G	0.033	-0.53 (0.47)	0.26
APOE ε4	Aβ42	Baseline level	494	ε4	ε2 or ε3	0.235	-0.66 (0.06)	< 2E-16
	tTau		617	ε4	ε2 or ε3	0.237	0.55 (0.06)	< 2E-16
	pTau		616	ε4	ε2 or ε3	0.237	0.59 (0.06)	< 2E-16
	Aβ42	Annual change	203	ε4	ε2 or ε3	0.239	-2.03 (7.52)	0.79
	tTau		274	ε4	ε2 or ε3	0.238	0.35 (1.85)	0.85
	pTau		273	ε4	ε2 or ε3	0.238	-0.06 (0.17)	0.72

Supplementary Table 6. Single SNP association test of rs117028417 with CSF biomarkers baseline level and longitudinal annual changes.

Gene	Biomarker	Biomarker Measure	N	Burden P-value	SKAT P-value
FAM222A	Aβ42	Baseline level	494	0.717	0.138
	tTau		617	0.416	0.254
	pTau		616	0.209	0.108
	Aβ42	Annual change	203	0.341	0.680
	tTau		274	0.183	0.219
	pTau		273	0.069	0.083
TRPV4	Aβ42	Baseline level	494	0.233	0.412
	tTau		617	0.934	0.276
	pTau		616	0.109	0.188
	Aβ42	Annual change	203	0.783	0.254
	tTau		274	0.679	0.298
	pTau		273	0.308	0.726

Supplementary Table 7. Gene-based association tests of *FAM222A* and *TRPV4* with CSF biomarkers baseline level and longitudinal annual changes.

Brain Region	AD			Control			Welch t-test P-value
	N	***FAM222A* mRNA level mean**	***FAM222A* mRNA level SD**	***N***	***FAM222A* mRNA level mean**	***FAM222A* mRNA level SD**	
Frontal Pole	42	3.57	0.43	21	3.48	0.26	0.312
Occipital Visual Cortex	32	3.65	0.39	21	3.64	0.36	0.901
Inferior Temporal Gyrus	38	4.18	0.49	20	3.95	0.50	0.103
Middle Temporal Gyrus	36	4.01	0.40	22	3.94	0.37	0.497
Superior Temporal Gyrus	37	3.57	0.44	23	3.63	0.64	0.697
Posterior Cingulate Cortex	39	3.93	0.51	19	4.15	0.67	0.219
Anterior Cingulate	36	3.88	0.50	23	3.84	0.63	0.813
Parahippocampal Gyrus	39	3.66	0.41	21	3.44	0.25	0.014
Temporal Pole	39	3.30	0.28	19	3.29	0.25	0.925
Precentral Gyrus	34	3.91	0.39	15	3.99	0.36	0.472
Inferior Frontal Gyrus	35	3.71	0.54	18	3.57	0.38	0.280
Dorsolateral Prefrontal Cortex	33	4.08	0.79	24	3.75	0.24	0.031
Superior Parietal Lobule	30	3.62	0.44	20	3.61	0.38	0.881
Prefrontal Cortex	39	3.77	0.38	17	3.55	0.28	0.019
Amygdala	34	4.29	0.47	17	4.45	0.53	0.292
Caudate Nucleus	37	4.51	0.52	15	4.61	0.82	0.663
Hippocampus	38	4.07	0.55	17	4.32	0.87	0.302
Nucleus Accumbens	34	4.45	0.76	17	4.51	0.68	0.783
Putamen	36	4.98	0.71	16	4.67	0.56	0.100

Supplementary Table 8. The *FAM222A* mRNA levels in 19 brain regions of AD patients and age-matched controls from the MSBB cohort.

Brain Region	N	PLQ_Mn			NTrSum			NPrSum		
		Effect	SE	P-Value	Effect	SE	P-Value	Effect	SE	P-Value
Frontal Pole	63	6.20	2.56	1.90E-02	4.76	2.74	8.74E-02	6.40	3.47	7.04E-02
Occipital Visual Cortex	53	-0.78	2.20	7.24E-01	-1.18	2.47	6.34E-01	-0.79	3.59	8.26E-01
Inferior Temporal Gyrus	58	6.19	2.10	4.87E-03	8.58	2.02	9.35E-05	8.08	2.77	5.24E-03
Middle Temporal Gyrus	58	7.04	2.73	1.28E-02	7.70	2.69	6.03E-03	6.40	3.61	8.23E-02
Superior Temporal Gyrus	60	1.22	2.02	5.48E-01	1.14	2.24	6.14E-01	-0.57	2.85	8.41E-01
Posterior Cingulate Cortex	58	-1.59	1.78	3.76E-01	-0.92	2.05	6.56E-01	-1.72	2.55	5.02E-01
Anterior Cingulate	59	-1.17	1.93	5.49E-01	0.16	2.02	9.35E-01	-2.57	2.55	3.19E-01
Parahippocampal Gyrus	60	1.93	2.41	4.25E-01	5.62	2.64	3.76E-02	2.46	3.52	4.87E-01
Temporal Pole	58	3.43	4.09	4.04E-01	5.84	4.41	1.91E-01	1.18	5.13	8.19E-01
Precentral Gyrus	49	-3.37	2.48	1.82E-01	-1.99	3.36	5.57E-01	-6.79	3.68	7.22E-02
Inferior Frontal Gyrus	53	5.59	2.13	1.17E-02	6.73	2.13	2.61E-03	5.46	2.98	7.34E-02
Dorsolateral Prefrontal Cortex	57	6.18	1.63	4.03E-04	3.67	1.72	3.82E-02	8.31	2.07	1.99E-04
Superior Parietal Lobule	50	-3.33	2.42	1.75E-01	0.66	2.62	8.02E-01	-5.86	3.67	1.17E-01
Prefrontal Cortex	56	4.04	3.27	2.22E-01	6.98	3.19	3.34E-02	4.89	4.10	2.38E-01
Amygdala	51	0.52	1.79	7.71E-01	0.01	2.21	9.96E-01	0.61	2.64	8.17E-01
Caudate Nucleus	52	-0.17	1.84	9.27E-01	-0.16	2.01	9.37E-01	0.13	2.50	9.58E-01
Hippocampus	55	1.17	1.39	4.03E-01	2.01	1.70	2.44E-01	1.67	2.06	4.22E-01
Nucleus Accumbens	51	0.82	1.37	5.53E-01	2.78	1.66	1.02E-01	0.31	1.93	8.75E-01
Putamen	52	1.57	1.81	3.89E-01	3.30	1.77	6.78E-02	1.70	2.13	4.29E-01

Supplementary Table 9. Association of *FAM222A* mRNA expression levels with three AD neuropathological phenotypes in the MSBB cohort. PLQ_Mn: plaque density mean; NTrSum: sum of neurofibrillary tangles density estimates; NPrSum: sum of neuritic plaque density estimates. To evaluate relationships between *FAM222A* mRNA expression and AD neuropathology, we conducted association analysis of *FAM222A* mRNA expression level with three neuropathological outcomes in MSBB cohort in 19 brain regions. *FAM222A* mRNA levels were significant associated with plaque density mean (PLQ_Mn) with P-value of 4.03×10^{-4} and sum of neuritic plaque (NPrSum) with P-value of $1.99\ \times10^{-4}$ passing Bonferroni adjusted threshold in dorsolateral prefrontal cortex. *FAM222A* mRNA levels were also tested to be significant associated neurofibrillary tangles density estimates (NTrSum) with P-value of $9.35\ \times10^{-5}$ in inferior temporal gyrus and P-value of 2.61×10^{-3} in inferior frontal gyrus.

Dataset	Brain tissue	Case *N*	Control *N*	CpG Site	Chr	Position (GRCh37)	Effect (SE)	P-value
E-GEOD-45775	Hippocampus	15	5	cg01335367	12	110172210	-8.8 (5.5)	0.11
E-GEOD-76105	Superior temporal gyrus	34	34	cg19879537	12	110157714	-8.3 (4.8)	0.08
				cg07775420	12	110159514	-9.1 (6.7)	0.17
				cg01335367	12	110172210	-7.0 (6.1)	0.26
				cg08141615	12	110172228	-7.4 (6.2)	0.23
				cg11365617	12	110174628	-32.2 (13.6)	0.01
				cg17840166	12	110177691	-24.0 (11.0)	0.02
				cg07658702	12	110182958	-5.3 (5.6)	0.35

Supplementary Table 10. *FAM222A* methylation association analysis with AD.

	Analysis population (*N* = 369)	CN (*N* = 120)	EMCI (*N* = 159)	LMCI (*N* = 64)	AD (*N* = 26)
**Cohort, n (%)**					
ADNI-GO	86 (23.3)	0 (0.0)	86 (54.1)	0 (0.0)	0 (0.0)
ADNI-2	283 (76.7)	120 (100.0)	73 (45.9)	64 (100.0)	26 (100.0)
**Age, mean years (SD)**	72.1 (7.3)	73.6 (6.2)	70.3 (7.4)	71.7 (7.6)	76.4 (7.1)
**Gender, n (%)**					
Male	199 (53.9)	63 (52.5)	89 (56.0)	31 (48.4)	16 (61.5)
Female	170 (46.1)	57 (47.5)	70 (44.0)	33 (51.6)	10 (38.5)
**Education, mean years (SD)**	16.5 (2.6)	16.8 (2.5)	16.2 (2.6)	16.9 (2.3)	15.7 (2.8)
**APOE ε4 risk alleles, n (%)**					
0 ε4	216 (58.5)	86 (71.7)	94 (59.1)	31 (48.4)	5 (19.2)
1 ε4	126 (34.1)	30 (25.0)	53 (33.3)	27 (42.2)	16 (61.5)
2 ε4	27 (7.3)	4 (3.3)	12 (7.5)	6 (9.4)	5 (19.2)

Supplementary Table 11. Demographics and characteristics of ADNI samples for PET AV-45 longitudinal change genetic association analysis. Abbreviation: CN, cognitively normal; EMCI, early mild cognitive impairment; LMCI, mild cognitive impairment; AD, Alzheimer's disease; APOE, Apolipoprotein E.

	Analysis population (*N* = 617)	CN (*N* = 202)	EMCI (*N* = 213)	LMCI (*N* = 158)	AD (*N* = 44)
**Cohort, n (%)**					
ADNI-1	131 (21.2)	65 (32.2)	0 (0.0)	66 (41.8)	0 (0.0)
ADNI-2	371 (60.1)	137 (67.8)	98 (45.9)	92 (58.2)	44 (100.0)
ADNI-GO	115 (18.6)	0 (0.0)	115 (54.0)		0 (0.0)
**Age, mean years (SD)**	72.9 (7.2)	74.4 (5.8)	71.1 (7.5)	72.7 (7.2)	75.6 (9.5)
**Gender, n (%)**					
Male	341 (55.3)	100 (49.5)	116 (54.5)	98 (62.0)	27 (61.4)
Female	276 (44.7)	102 (50.5)	97 (45.5)	60 (38.0)	17 (38.6)
**Education, mean years (SD)**	16.2 (2.7)	16.3 (2.7)	15.8 (2.6)	16.5 (2.8)	15.6 (2.7)
**APOE ε4 risk alleles, n (%)**					
0 ε4	372 (60.3)	154 (76.2)	131 (61.5)	74 (46.8)	13 (29.5)
1 ε4	197 (31.9)	41 (20.3)	69 (32.4)	65 (41.1)	22 (50.0)
2 ε4	48 (7.8)	7 (3.5)	13 (6.1)	19 (12.0)	9 (20.5)
**Elecsys CSF biomarker, median pg/mL (MAD)**					
Aβ42	964.7 (561.6)	1290 (607.9)	1085 (610.0)	750.8 (306.5)	617.6 (224.0)
tTau	245.4 (97.0)	218.8 (75.4)	234 (90.0)	287.8 (122.5)	334.5 (128.3)
pTau	22.2 (10.0)	19.9 (7.2)	20.8 (8.7)	27.6 (13.1)	33.4 (15.0)

Supplementary Table 12. Demographics and characteristics of ADNI samples for CSF biomarkers baseline level genetic association analysis. Abbreviation: CN, cognitively normal; EMCI, early mild cognitive impairment; LMCI, late mild cognitive impairment; AD, Alzheimer's disease; APOE, Apolipoprotein E; CSF, cerebrospinal fluid; tTau, total tau; pTau, phosphorylated tau; MAD, median absolute deviation.

	Analysis population (N = 274)	CN (N = 91)	EMCI (N = 97)	LMCI (N = 70)	AD (N = 16)
**Cohort, n (%)**					
ADNI-1	40 (14.6)	17 (18.7)	0 (0.0)	23 (32.9)	0 (0.0)
ADNI-2	177 (64.6)	74 (81.3)	40 (41.2)	47 (67.1)	16 (100.0)
ADNI-GO	57 (20.8)	0 (0.0)	57 (58.8)		0 (0.0)
**Age, mean years (SD)**	72.8 (7.0)	74.2 (5.9)	71.1 (7.5)	72.1 (6.9)	77.4 (7.2)
**Gender, n (%)**					
Male	149 (54.4)	47 (51.7)	51 (52.6)	41 (58.6)	10 (62.5)
Female	125 (45.6)	44 (48.3)	46 (47.4)	29 (41.4)	6 (37.5)
**Education, mean years (SD)**	16.5 (2.6)	17.1 (2.4)	15.9 (2.7)	16.7 (2.4)	15.4 (3.3)
**APOE ε4 risk alleles, n (%)**					
0 ε4	162 (59.1)	69 (75.8)	55 (56.7)	34 (48.6)	4 (25.0)
1 ε4	90 (32.8)	20 (22.0)	33 (34.0)	30 (42.9)	7 (43.8)
2 ε4	22 (8.0)	2 (2.2)	9 (9.3)	6 (8.6)	5 (31.2)
**Elecsys CSF biomarker change, mean pg/mL/year (SD)**					
Aβ42	-21.5 (69.6)	-19.3 (90.6)	-13.4 (69.3)	-34.4 (49.0)	-15.3 (44.5)
tTau	5.9 (19.1)	5.4 (12.0)	3.5 (19.7)	7.8 (18.1)	15.0 (40.1)
pTau	0.4 (1.8)	0.6 (1.2)	0.4 (2.1)	0.4 (1.6)	0.06 (2.3)

Supplementary Table 13. Demographics and characteristics of ADNI samples for CSF biomarkers longitudinal change genetic association analysis. Abbreviation: CN, cognitively normal; EMCI, early mild cognitive impairment; LMCI, late mild cognitive impairment; AD, Alzheimer's disease; APOE, Apolipoprotein E; CSF, cerebrospinal fluid; tTau, total tau; pTau, phosphorylated tau; MAD, median absolute deviation.

	Analysis population (*N* = 20)	Control (*N* = 5)	Alzheimer Braak Stage I-II (*N* = 5)	Alzheimer Braak Stage III-IV (*N* = 5)	Alzheimer Braak Stage V-VI (*N* = 5)
**Gender, n (%)**					
Male	12 (60)	3 (60)	1 (20)	4 (80)	4 (80)
Female	8 (40)	2 (40)	4 (80)	1 (20)	1 (20)

Supplementary Table 14. Demographics and characteristics of EMBL-EBI study E-GEOD-45775.

	Analysis population (*N* = 68)	Control (*N* = 34)	AD (*N* = 34)
**Neuronal cells proportion, mean (SD)**	0.28 (0.07)	0.30 (0.07)	0.25 (0.06)
**Age, mean years (SD)**	79.8 (7.8)	80.6 (8.1)	79.0 (7.5)
**Gender, n (%)**			
Male	33 (48.5)	17 (50)	16 (47.1)
Female	35 (51.5)	17 (50)	18 (52.9)

Supplementary Table 15. Demographics and characteristics of EMBL-EBI study E-GEOD-70615.

Diagnosis	PMI (hours)	Age (years)	Disease stage	Gender	ApoE status
Control	N/A	40	Braak 0	M	N/A
Control	N/A	48	Braak 0	M	N/A
Control	2.5	50	Braak 0	M	E3/E3
Control	9	62	Braak 0	M	N/A
Control	46	66	Braak 0	M	N/A
Control	4	69	Braak I	F	N/A
Control	25	74	Braak 0	F	N/A
Control	4	80	Braak I/II	M	N/A
Control	22	82	Braak I	M	N/A
Control	5	84	Braak I	M	E3/E4
Control	10	86	Braak I/II	M	N/A
Control	4.5	92	Braak I	F	E3/E3
AD	25	64	A2,B2,C0/Braak III/IV	M	E3/E4
AD	26	67	A2,B3,C2/Braak V/VI	M	E4/E4
AD	3	68	Braak VI	F	E3/E4
AD	29	68	Braak IV	M	N/A
AD	4	69	Braak V/VI	M	E3/E4
AD	8	69	Braak V/VI	M	E3/E3
AD	19	72	A3,B2,C2/Braak III/IV	M	E3/E4
AD	12	72	Braak III	F	N/A
AD	9	72	Braak III	F	E3/E3
AD	19	72	A3,B2,C2/Braak III/IV	M	N/A
AD	46	73	A3,B3,C2/Braak V/VI	M	E3/E3
AD	5	73	Braak V/VI	M	E4/E4
AD	5	75	A3,B3,C3//Braak V/VI	M	E3/E3
AD	30	78	Braak II-III	F	N/A
AD	4	79	Braak III	M	E4/E4
AD	3	80	Braak III-IV	F	N/A
AD	26	84	Braak VI	N/A	N/A
AD	6	84	Braak V	F	N/A
AD	55	84	Braak IV	F	N/A
AD	31	89	A2, B1,C1/ Braak I	F	E3/E3
AD	2	90	Braak V/VI	F	E3/E4
AD	5	90	Braak V-VI	F	N/A
AD-PS1 mutation	N/A	38	N/A	M	N/A
AD-APP mutation	N/A	N/A	N/A	N/A	N/A
DS	N/A	31	N/A	M	N/A
DS	26	61	N/A	M	N/A
DS	N/A	65	N/A	N/A	N/A

Supplementary Table 16. Paraffin sections for immunocytochemistry and immunofluorescence. The neuropathologic assessment is based on the Braak staging and “ABC” scores (A: amyloid β deposits (A); B: staging of neurofibrillary tangles; and C: scoring of neuritic plaques). M: male, F: female; E3: APOE3, E4: APOE4. N/A: the information is not available. Note that for AD cases bearing APP/PS1 mutation or DS cases, the Braak staging information is not available. We may not be able to draw any conclusion based on the limited number of cases. All control cases are not non-demented individuals with AD neuropathological changes (ADNC) or individuals with primary age-related tauopathy (PART).

Diagnosis	PMI (Hours)	Age (Years)	Disease stage	Gender	ApoE status
**Diagnosis**	**PMI (Hours)**	**Age (Years)**	**Disease stage**	**Gender**	**ApoE status**
Control	17	66	Braak 0	M	E2/E3
Control	19	73	Braak I	F	E3/E3
Control	12	74	Braak I	F	E3/E3
Control	22	76	Braak I	M	E3/E3
Control	12	80	Braak I/II	M	E3/E3
Control	18	83	Braak I	F	E3/E3
Control	12	84	Braak I/II	M	E3/E3
AD	6	N/A	A2,B2,C3/Braak III/IV	M	E3/E3
AD	4	55	A3,B3,C3/Braak VI	F	E3/E4
AD	4	73	Braak VI	F	E3/E4
AD	4	78	Braak VI	M	E3/E4
AD	29	83	Braak V/VI	M	E3/E3
AD	35	84	A3,B3,C2/Braak V/VI	F	E4/E4
AD	6	87	A3,B2,C3/ Braak III/IV	F	E3/E3
AD	3	87	A3,B3,C3/Braak V/VI	F	E3/E4
AD	4	88	Braak III/IV	F	E3/E4

Supplementary Table 17. Frozen tissues for biochemical analysis. The neuropathologic assessment is based on the Braak staging and "ABC" scores (A: amyloid β deposits (A); B: staging of neurofibrillary tangles; and C: scoring of neuritic plaques). M: male, F: female; E3: APOE3, E4: APOE4. N/A: the information is not available. All control cases are not ADNC or PART.

Antibody	Application	Host	Source	Catalogue#
22C11	WB	Mouse monoclonal	EMD Millipore	MAB348
4G8	IF	Mouse monoclonal	BioLegend	SIG-39220
6E10	WB/IF/IHC	Mouse monoclonal	BioLegend	803001
82E1	IF	Mouse monoclonal	IBL	10323
Actin	WB	Mouse monoclonal	EMD Millipore	MAB1501
Aggregatin	WB/IF/IHC	Rabbit polyclonal	Abcam	ab122626
Aggregatin	IHC	Rabbit polyclonal	LifeSpan BioSciences	LS-C170630
Aggregatin	IHC	Rabbit polyclonal	Aviva Systems Biology	ARP69038_P050
APOE	WB	Rabbit polyclonal	DAKO	A0077
Calnexin	WB	Rabbit polyclonal	ENZO	ADI-SPA-860
Flag	WB/IHC	Mouse monoclonal	Sigma Aldrich	F1804
COX IV	WB	Mouse monoclonal	Cell Signaling Technology	11967S
Flag	IHC	Rabbit polyclonal	Thermo Fisher	PA1-984B
Flag	IHC	Rabbit polyclonal	Cell Signaling Technology	2368
Flag-HRP	IHC	Mouse monoclonal	Proteintech	HRP-66008
GAPDH	WB	Rabbit monoclonal	Cell Signaling Technology	2118
GFP	WB/IHC	Rabbit monoclonal	Abcam	ab32146
MOAB-2-HRP	ELISA	Mouse monoclonal	Novus	NBP2-13075H
Myc	WB/IHC	Mouse monoclonal	Thermo Fisher	MA1-21316
Myc	IHC	Mouse monoclonal	Cell Signaling Technology	2276
Nu4	IF/IHC	Mouse monoclonal	Klein lab	NA
TSG101	WB	Mouse monoclonal	BD biosciences	612696

Supplementary Table 18. **Antibodies used in this study.** WB: western blot; IF: Immunofluorescence; IHC: Immunohistochemistry.

Name	Primer Sequence(5'-3')
**Aggregatin-F**	GGATCCATGCTGGCCTGTCTGCAGAG
**Aggregatin-R**	TCTAGATTATCTGTAGACGGGTAGGCGGAT
**1-40-F**	GGATCCATGCTGGCCTGTCTGCAGAG
**1-40-R**	TCTAGATTAGTAGCGGGAGGAATGCATGGCG
**1-60-F**	GGATCCATGCTGGCCTGTCTGCAGAG
**1-60-R**	TCTAGATTAGATGGACAGCGGGCTGTTGGC
**1-65-F**	GGATCCATGCTGGCCTGTCTGCAGAG
**1-65-R**	TCTAGATTAGGTGGGGAAGATCTTGATGGA
**1-70-F**	GGATCCATGCTGGCCTGTCTGCAGAG
**1-70-R**	TCTAGATTAGGGCACACGGATGTTGGTGG
**1-75-F**	GGATCCATGCTGGCCTGTCTGCAGAG
**1-75-R**	TCTAGATTAGAGGTGCTTGTGCTGGGGCA
**1-80-F**	GGATCCATGCTGGCCTGTCTGCAGAG
**1-80-R**	TCTAGATTAATTGACTGTGCGGCTGAGGTG
**1-150-F**	GGATCCATGCTGGCCTGTCTGCAGAG
**1-150-R**	TCTAGATTACACTGGGTAGGGCGCAATGC
**151-300-F**	GGATCCCCCAGCACTCTGGGTCCCTTG
**151-300-R**	TCTAGATTACAGTGGCTGGGGCGGCGGTGG
**301-452-F**	GGATCCCGTGCCTACAGTGGGAGCACG
**301-452-R**	TCTAGATTATCTGTAGACGGGTAGGCGGAT
**21-80-F**	GGATCCAAGAGCCTGGAGCTGCGCAAGT
**21-80-R**	TCTAGATTAATTGACTGTGCGGCTGAGGTG
**31-80-F**	GGATCCGTGGCCAGCGCCATGCATTCCT
**31-80-R**	TCTAGATTAATTGACTGTGCGGCTGAGGTG
**41-80-F**	GGATCCCCGAGCCCAGCAGAACTGGACG
**41-80-R**	TCTAGATTAATTGACTGTGCGGCTGAGGTG
**51-80-F**	GGATCCGAGAAGGTGGCCAACAGCCCGC
**51-80-R**	TCTAGATTAATTGACTGTGCGGCTGAGGTG
**56-75-F**	gattcAGCCCGCTGTCCATCAAGATCTTCCCCACCAACATCCGTGTGCCCCAGCACAAGCACCTCTAAt
**56-75-R**	ctagaTTAGAGGTGCTTGTGCTGGGGCACACGGATGTTGGTGGGGAAGATCTTGATGGACAGCGGGCTg
**61-80-F**	gatccAAGATCTTCCCCACCAACATCCGTGTGCCCCAGCACAAGCACCTCAGCCGCACAGTCAATTAAt
**61-80-R**	ctagaTTAATTGACTGTGCGGCTGAGGTGCTTGTGCTGGGGCACACGGATGTTGGTGGGGAAGATCTTg
**61-70-F**	gatccAAGATCTTCCCCACCAACATCCGTGTGCCCTAAt
**61-70-R**	ctagaTTAGGGCACACGGATGTTGGTGGGGAAGATCTTg
**66-75-F**	gatccAACATCCGTGTGCCCCAGCACAAGCACCTCTAAt
**66-75-R**	ctagaTTAGAGGTGCTTGTGCTGGGGCACACGGATGTTg
**71-80-F**	gatccCAGCACAAGCACCTCAGCCGCACAGTCAATTAAt
**71-80-R**	ctagaTTAATTGACTGTGCGGCTGAGGTGCTTGTGCTGg
**81-452-F**	GGATCCGGCTATGACACCAGTGGCCAGC
**81-452-R**	TCTAGATTATCTGTAGACGGGTAGGCGGAT
**91-452-F**	GGATCCCCCTACCCACAGCACACCGCTG
**91-452-R**	TCTAGATTATCTGTAGACGGGTAGGCGGAT
**101-452-F**	GGATCCGGCCTTCTGGCCATTGTCAAGG
**101-452-R**	TCTAGATTATCTGTAGACGGGTAGGCGGAT
**Δ61-80-F**	GGATCCATGCTGGCCTGTCTGCAGAG
**Δ61-80-middle-R**	TGGCCACTGGTGTCATAGCCGATGGACAGCGGGCTGTTGGCC
**Δ61-80-middle-F**	GGCTATGACACCAGTGGCCAGC
**Δ61-80-R**	TCTAGATTATCTGTAGACGGGTAGGCGGAT
**Δ81-180-F**	GGATCCATGCTGGCCTGTCTGCAGAG
**Δ81-180-middle-R**	CGGCAGGGGGATGACATTGACTGTGCGGCTGAGGTG
**Δ81-180-middle-F**	GTCATCCCCCTGCCGGGCC
**Δ81-180-R**	TCTAGATTATCTGTAGACGGGTAGGCGGAT
**Δ171-270-F**	GGATCCATGCTGGCCTGTCTGCAGAG
**Δ171-270-middle-R**	TGCAGGCTTGGCGGCCCCGGGTGGTGGAGCAGGCGCCT
**Δ171-270-middle-F**	GGGGCCGCCAAGCCTGCAGGG
**Δ171-270-R**	TCTAGATTATCTGTAGACGGGTAGGCGGAT
**Δ261-360-F**	GGATCCATGCTGGCCTGTCTGCAGAG
**Δ261-360-middle-R**	CGCCGCCGCTGGGTTGTAGCCCAGCTCAGTGCCTTTCCG
**Δ261-360-middle-F**	TACAACCCAGCGGCGGCGGTG
**Δ261-360-R**	TCTAGATTATCTGTAGACGGGTAGGCGGAT
**Δ61-65-F**	GGATCCATGCTGGCCTGTCTGCAGAG
**Δ61-65-middle-R**	TGCTGGGGCACACGGATGTTGATGGACAGCGGGCTGTTGGCC
**Δ61-65-middle-F**	AACATCCGTGTGCCCCAGCACA
**Δ61-65-R**	TCTAGATTATCTGTAGACGGGTAGGCGGAT
**Δ66-70-F**	GGATCCATGCTGGCCTGTCTGCAGAG
**Δ66-70-middle-R**	CGGCTGAGGTGCTTGTGCTGGGTGGGGAAGATCTTGATGGAC
**Δ66-70-middle-F**	CAGCACAAGCACCTCAGCCGCA
**Δ66-70-R**	TCTAGATTATCTGTAGACGGGTAGGCGGAT
**Δ71-75-F**	GGATCCATGCTGGCCTGTCTGCAGAG
**Δ71-75-middle-R**	TAGCCATTGACTGTGCGGCTGGGCACACGGATGTTGGTGGGG
**Δ71-75-middle-F**	AGCCGCACAGTCAATGGCTATG
**Δ71-75-R**	TCTAGATTATCTGTAGACGGGTAGGCGGAT
**Δ76-80-F**	GGATCCATGCTGGCCTGTCTGCAGAG
**Δ76-80-middle-R**	TGGCCACTGGTGTCATAGCCGAGGTGCTTGTGCTGGGGCACA
**Δ76-80-middle-F**	GGCTATGACACCAGTGGCCAGC
**Δ76-80-R**	TCTAGATTATCTGTAGACGGGTAGGCGGAT
**3Myc-2LST-Aggregatin-F**	GGATCCATGCTGGCCTGTCTGCAGAG
**3Myc-2LST-Aggregatin-R**	CTCGAGTTAATTGACTGTGCGGCTGAGGTG

Supplementary Table 19. Primers used in this study. Primers used for constructing recombinant protein expression vectors (F: forward primer; R: reverse primer)

Supplementary References:

1. Wang, M., *et al.* Integrative network analysis of nineteen brain regions identifies molecular signatures and networks underlying selective regional vulnerability to Alzheimer's disease. *Genome Med* **8**, 104 (2016).
2. Hibar, D.P., *et al.* Common genetic variants influence human subcortical brain structures. *Nature* **520**, 224-229 (2015).
3. Patenaude, B., Smith, S.M., Kennedy, D.N. & Jenkinson, M. A Bayesian model of shape and appearance for subcortical brain segmentation. *Neuroimage* **56**, 907-922 (2011).
