# Supplementary material for: FAM222A encodes a protein which accumulates in plaques in Alzheimer’s disease
Source: Nat Commun. 2020 Jan 21;11:411. doi: 10.1038/s41467-019-13962-0 (PMC6972869; doi:10.1038/s41467-019-13962-0)
Supplement: Supplementary file 2 — Description of Additional Supplementary Files [file 41467_2019_13962_MOESM2_ESM.pdf]

File Name: Supplementary Data 1

Description: Sequences of Aggregatin and its mutant with N-terminal 4xFlag 1063 tag-Twin-Strep-tag (4xFlag-TST) or 3xMyc tag-Twin-Strep-tag (3xMyc-TST).
