# Supplementary material for: FAM222A encodes a protein which accumulates in plaques in Alzheimer’s disease
Source: Nat Commun. 2020 Jan 21;11:411. doi: 10.1038/s41467-019-13962-0 (PMC6972869; doi:10.1038/s41467-019-13962-0)
Supplement: Supplementary file 3 — Supplementary Data 1 [file 41467_2019_13962_MOESM3_ESM.pdf]

4xFlag-TST-Aggregatin full length

ATGAGATCTGACTACAAGGACGACGACGACAAGGGATCTGATTATAAAGATGACGATGATAAGGGATCTGACTACAAAGACGAT
GATGACAAAGGATCTGATTACAAGGATGATGACGATAAAGGATCTGCATGGAGCCATCCCCAGTTTGAGAAAGGTGGCGGATC
AGGCGGGGGTTCTGGTGGATCTGCATGGAGCCATCCCCAGTTTGAGAAAGGTGGCGGATCAGGCGGGGGTTCTGGTGGATCC
ATGCTGGCCTGTCTGCAGAGGACCCAGAACGCCCCGGGCCAACACCTGGCCTGCCCGAGCAAGAGCCTGGAGCTGCGCAAG
TGCGAGGCGGTGGCCAGCGCCATGCATTCCTCCCGCTACCCGAGCCCAGCAGAACTGGACGCCTATGCCGAGAAGGTGGCC
AACAGCCCGCTGTCCATCAAGATCTTCCCCACCAACATCCGTGTGCCCCAGCACAAGCACCTCAGCCGCACAGTCAATGGCTA
TGACACCAGTGGCCAGCGCTACAGCCCCTACCCACAGCACACCGCTGGCTACCAGGGCCTTCTGGCCATTGTCAAGGCCGCG
GTTTCCTCCTCCAGCACGGCCGCACCAGCTGGGCCCGCCAAAAGTGTGCTCAAGAGCGCCGAGGGCAAGCGGACCAAGCTGT
CACCGGCCGCCGTGCAGGTGGGCATTGCGCCCTACCCAGTGCCCAGCACTCTGGGTCCCTTGGCCTACCCCAAGCCACCTGA
GGCGCCTGCTCCACCACCCGGCCTGCCCGCAGCCGCCACTGCCGCCTCCGTCATCCCCCTGCCGGGCCGGGGCCTGCCCCT
GCCACCTTCCAACCTGCCCTCCATCCACAGCCTCCTGTACCAGCTCAACCAGCAGTGCCAGGCCCCGGGCGCCGCACCCCCT
GCCTGCCAGGGCATGGCTATTCCCCATCCCAGCCCTGCCAAGCACGGCCCAGTGCCCAGCTTCCCCAGCATGGCCTACTCGG
CTGCAGCCGGTCTGCCCGACTGCCGGAAAGGCACTGAGCTGGGCCAGGGAGCCACCCAAGCCTTGACGTTGGCTGGGGCCG
CCAAGCCTGCAGGGTACGCAGACAGCGGCCTGGATTACCTGCTGTGGCCGCAGAAACCGCCCCCACCGCCGCCCCAGCCAC
TGCGTGCCTACAGTGGGAGCACGGTGGCCAGCAAGTCCCCTGAGGCTTGCGGGGGCCGGGCATACGAGCGGGCCAGCGGG
TCACCCCTCAACTGTGGCGTGGGGCTGCCCACCAGCTTCACCGTAGGCCAGTACTTTGCGGCCCCGTGGAACAGTGTGCTGG
TGACACCCACCAGCGACTGCTACAACCCAGCGGCGGCGGTGGTGGTCACGGAGCTGGGGCCGGGGGCAGCCCGGGAGCTG
GCTGGGCCCCCTGCAGATGCCCTCTCGGGCCTGCCCAGCAAGAGTGTGTGCAACACATCGGTGCTGAGCAGCAGCCTGCAGT
CACTGGAGTATCTCATCAACGACATCCGGCCGCCCTGCATCAAGGAGCAGATGCTGGGCAAGGGCTATGAGACGGTGGCCGT
GCCCCGGCTACTCGACCACCAGCATGCCCACATCCGCCTACCCGTCTACAGATAA

4xFlag-TST-Aggregatin (1-40)

ATGAGATCTGACTACAAGGACGACGACGACAAGGGATCTGATTATAAAGATGACGATGATAAGGGATCTGACTACAAAGACGAT
GATGACAAAGGATCTGATTACAAGGATGATGACGATAAAGGATCTGCATGGAGCCATCCCCAGTTTGAGAAAGGTGGCGGATC
AGGCGGGGGTTCTGGTGGATCTGCATGGAGCCATCCCCAGTTTGAGAAAGGTGGCGGATCAGGCGGGGGTTCTGGTGGATCC
ATGCTGGCCTGTCTGCAGAGGACCCAGAACGCCCCGGGCCAACACCTGGCCTGCCCGAGCAAGAGCCTGGAGCTGCGCAAG
TGCGAGGCGGTGGCCAGCGCCATGCATTCCTCCCGCTACTAA

4xFlag-TST-Aggregatin (1-60)

ATGAGATCTGACTACAAGGACGACGACGACAAGGGATCTGATTATAAAGATGACGATGATAAGGGATCTGACTACAAAGACGAT
GATGACAAAGGATCTGATTACAAGGATGATGACGATAAAGGATCTGCATGGAGCCATCCCCAGTTTGAGAAAGGTGGCGGATC
AGGCGGGGGTTCTGGTGGATCTGCATGGAGCCATCCCCAGTTTGAGAAAGGTGGCGGATCAGGCGGGGGTTCTGGTGGATCC
ATGCTGGCCTGTCTGCAGAGGACCCAGAACGCCCCGGGCCAACACCTGGCCTGCCCGAGCAAGAGCCTGGAGCTGCGCAAG
TGCGAGGCGGTGGCCAGCGCCATGCATTCCTCCCGCTACCCGAGCCCAGCAGAACTGGACGCCTATGCCGAGAAGGTGGCC
AACAGCCCGCTGTCCATCTAA

4xFlag-TST-Aggregatin (1-65)

ATGAGATCTGACTACAAGGACGACGACGACAAGGGATCTGATTATAAAGATGACGATGATAAGGGATCTGACTACAAAGACGAT
GATGACAAAGGATCTGATTACAAGGATGATGACGATAAAGGATCTGCATGGAGCCATCCCCAGTTTGAGAAAGGTGGCGGATC
AGGCGGGGGTTCTGGTGGATCTGCATGGAGCCATCCCCAGTTTGAGAAAGGTGGCGGATCAGGCGGGGGTTCTGGTGGATCC
ATGCTGGCCTGTCTGCAGAGGACCCAGAACGCCCCGGGCCAACACCTGGCCTGCCCGAGCAAGAGCCTGGAGCTGCGCAAG
TGCGAGGCGGTGGCCAGCGCCATGCATTCCTCCCGCTACCCGAGCCCAGCAGAACTGGACGCCTATGCCGAGAAGGTGGCC
AACAGCCCGCTGTCCATCAAGATCTTCCCCACCTAA

4xFlag-TST-Aggregatin (1-70)

ATGAGATCTGACTACAAGGACGACGACGACAAGGGATCTGATTATAAAGATGACGATGATAAGGGATCTGACTACAAAGACGAT
GATGACAAAGGATCTGATTACAAGGATGATGACGATAAAGGATCTGCATGGAGCCATCCCCAGTTTGAGAAAGGTGGCGGATC
AGGCGGGGGTTCTGGTGGATCTGCATGGAGCCATCCCCAGTTTGAGAAAGGTGGCGGATCAGGCGGGGGTTCTGGTGGATCC
ATGCTGGCCTGTCTGCAGAGGACCCAGAACGCCCCGGGCCAACACCTGGCCTGCCCGAGCAAGAGCCTGGAGCTGCGCAAG
TGCGAGGCGGTGGCCAGCGCCATGCATTCCTCCCGCTACCCGAGCCCAGCAGAACTGGACGCCTATGCCGAGAAGGTGGCC
AACAGCCCGCTGTCCATCAAGATCTTCCCCACCAACATCCGTGTGCCCTAA

4xFlag-TST-Aggregatin (1-75)

ATGAGATCTGACTACAAGGACGACGACGACAAGGGATCTGATTATAAAGATGACGATGATAAGGGATCTGACTACAAAGACGAT
GATGACAAAGGATCTGATTACAAGGATGATGACGATAAAGGATCTGCATGGAGCCATCCCCAGTTTGAGAAAGGTGGCGGATC
AGGCGGGGGTTCTGGTGGATCTGCATGGAGCCATCCCCAGTTTGAGAAAGGTGGCGGATCAGGCGGGGGTTCTGGTGGATCC
ATGCTGGCCTGTCTGCAGAGGACCCAGAACGCCCCGGGCCAACACCTGGCCTGCCCGAGCAAGAGCCTGGAGCTGCGCAAG
TGCGAGGCGGTGGCCAGCGCCATGCATTCCTCCCGCTACCCGAGCCCAGCAGAACTGGACGCCTATGCCGAGAAGGTGGCC
AACAGCCCGCTGTCCATCAAGATCTTCCCCACCAACATCCGTGTGCCCCAGCACAAGCACCTCTAA

4xFlag-TST-Aggregatin (1-80)

ATGAGATCTGACTACAAGGACGACGACGACAAGGGATCTGATTATAAAGATGACGATGATAAGGGATCTGACTACAAAGACGAT
GATGACAAAGGATCTGATTACAAGGATGATGACGATAAAGGATCTGCATGGAGCCATCCCCAGTTTGAGAAAGGTGGCGGATC
AGGCGGGGGTTCTGGTGGATCTGCATGGAGCCATCCCCAGTTTGAGAAAGGTGGCGGATCAGGCGGGGGTTCTGGTGGATCC
ATGCTGGCCTGTCTGCAGAGGACCCAGAACGCCCCGGGCCAACACCTGGCCTGCCCGAGCAAGAGCCTGGAGCTGCGCAAG
TGCGAGGCGGTGGCCAGCGCCATGCATTCCTCCCGCTACCCGAGCCCAGCAGAACTGGACGCCTATGCCGAGAAGGTGGCC
AACAGCCCGCTGTCCATCAAGATCTTCCCCACCAACATCCGTGTGCCCCAGCACAAGCACCTCAGCCGCACAGTCAATTAA

4xFlag-TST-Aggregatin (1-150)

ATGAGATCTGACTACAAGGACGACGACGACAAGGGATCTGATTATAAAGATGACGATGATAAGGGATCTGACTACAAAGACGAT
GATGACAAAGGATCTGATTACAAGGATGATGACGATAAAGGATCTGCATGGAGCCATCCCCAGTTTGAGAAAGGTGGCGGATC
AGGCGGGGGTTCTGGTGGATCTGCATGGAGCCATCCCCAGTTTGAGAAAGGTGGCGGATCAGGCGGGGGTTCTGGTGGATCC
ATGCTGGCCTGTCTGCAGAGGACCCAGAACGCCCCGGGCCAACACCTGGCCTGCCCGAGCAAGAGCCTGGAGCTGCGCAAG
TGCGAGGCGGTGGCCAGCGCCATGCATTCCTCCCGCTACCCGAGCCCAGCAGAACTGGACGCCTATGCCGAGAAGGTGGCC
AACAGCCCGCTGTCCATCAAGATCTTCCCCACCAACATCCGTGTGCCCCAGCACAAGCACCTCAGCCGCACAGTCAATGGCTA
TGACACCAGTGGCCAGCGCTACAGCCCCTACCCACAGCACACCGCTGGCTACCAGGGCCTTCTGGCCATTGTCAAGGCCGCG
GTTTCCTCCTCCAGCACGGCCGCACCAGCTGGGCCCGCCAAAAGTGTGCTCAAGAGCGCCGAGGGCAAGCGGACCAAGCTGT
CACCGGCCGCCGTGCAGGTGGGCATTGCGCCCTACCCAGTGTAA

4xFlag-TST-Aggregatin (1-350)

ATGAGATCTGACTACAAGGACGACGACGACAAGGGATCTGATTATAAAGATGACGATGATAAGGGATCTGACTACAAAGACGAT
GATGACAAAGGATCTGATTACAAGGATGATGACGATAAAGGATCTGCATGGAGCCATCCCCAGTTTGAGAAAGGTGGCGGATC
AGGCGGGGGTTCTGGTGGATCTGCATGGAGCCATCCCCAGTTTGAGAAAGGTGGCGGATCAGGCGGGGGTTCTGGTGGATCC
ATGCTGGCCTGTCTGCAGAGGACCCAGAACGCCCCGGGCCAACACCTGGCCTGCCCGAGCAAGAGCCTGGAGCTGCGCAAG
TGCGAGGCGGTGGCCAGCGCCATGCATTCCTCCCGCTACCCGAGCCCAGCAGAACTGGACGCCTATGCCGAGAAGGTGGCC
AACAGCCCGCTGTCCATCAAGATCTTCCCCACCAACATCCGTGTGCCCCAGCACAAGCACCTCAGCCGCACAGTCAATGGCTA
TGACACCAGTGGCCAGCGCTACAGCCCCTACCCACAGCACACCGCTGGCTACCAGGGCCTTCTGGCCATTGTCAAGGCCGCG
GTTTCCTCCTCCAGCACGGCCGCACCAGCTGGGCCCGCCAAAAGTGTGCTCAAGAGCGCCGAGGGCAAGCGGACCAAGCTGT
CACCGGCCGCCGTGCAGGTGGGCATTGCGCCCTACCCAGTGCCCAGCACTCTGGGTCCCTTGGCCTACCCCAAGCCACCTGA
GGCGCCTGCTCCACCACCCGGCCTGCCCGCAGCCGCCACTGCCGCCTCCGTCATCCCCCTGCCGGGCCGGGGCCTGCCCCT
GCCACCTTCCAACCTGCCCTCCATCCACAGCCTCCTGTACCAGCTCAACCAGCAGTGCCAGGCCCCGGGCGCCGCACCCCCT
GCCTGCCAGGGCATGGCTATTCCCCATCCCAGCCCTGCCAAGCACGGCCCAGTGCCCAGCTTCCCCAGCATGGCCTACTCGG
CTGCAGCCGGTCTGCCCGACTGCCGGAAAGGCACTGAGCTGGGCCAGGGAGCCACCCAAGCCTTGACGTTGGCTGGGGCCG
CCAAGCCTGCAGGGTACGCAGACAGCGGCCTGGATTACCTGCTGTGGCCGCAGAAACCGCCCCCACCGCCGCCCCAGCCAC
TGCGTGCCTACAGTGGGAGCACGGTGGCCAGCAAGTCCCCTGAGGCTTGCGGGGGCCGGGCATACGAGCGGGCCAGCGGG
TCACCCCTCAACTGTGGCGTGGGGCTGCCCACCAGCTTCACCGTAGGCCAGTACTTTGCGGCCCCGTGGAACTAA

4xFlag-TST-Aggregatin (91-452)

ATGAGATCTGACTACAAGGACGACGACGACAAGGGATCTGATTATAAAGATGACGATGATAAGGGATCTGACTACAAAGACGAT
GATGACAAAGGATCTGATTACAAGGATGATGACGATAAAGGATCTGCATGGAGCCATCCCCAGTTTGAGAAAGGTGGCGGATC
AGGCGGGGGTTCTGGTGGATCTGCATGGAGCCATCCCCAGTTTGAGAAAGGTGGCGGATCAGGCGGGGGTTCTGGTGGATCC
CCCTACCCACAGCACACCGCTGGCTACCAGGGCCTTCTGGCCATTGTCAAGGCCGCGGTTTCCTCCTCCAGCACGGCCGCAC
CAGCTGGGCCCGCCAAAAGTGTGCTCAAGAGCGCCGAGGGCAAGCGGACCAAGCTGTCACCGGCCGCCGTGCAGGTGGGCA
TTGCGCCCTACCCAGTGCCCAGCACTCTGGGTCCCTTGGCCTACCCCAAGCCACCTGAGGCGCCTGCTCCACCACCCGGCCT
GCCCGCAGCCGCCACTGCCGCCTCCGTCATCCCCCTGCCGGGCCGGGGCCTGCCCCTGCCACCTTCCAACCTGCCCTCCATC
CACAGCCTCCTGTACCAGCTCAACCAGCAGTGCCAGGCCCCGGGCGCCGCACCCCCTGCCTGCCAGGGCATGGCTATTCCCC
ATCCCAGCCCTGCCAAGCACGGCCCAGTGCCCAGCTTCCCCAGCATGGCCTACTCGGCTGCAGCCGGTCTGCCCGACTGCCG
GAAAGGCACTGAGCTGGGCCAGGGAGCCACCCAAGCCTTGACGTTGGCTGGGGCCGCCAAGCCTGCAGGGTACGCAGACAG
CGGCCTGGATTACCTGCTGTGGCCGCAGAAACCGCCCCCACCGCCGCCCCAGCCACTGCGTGCCTACAGTGGGAGCACGGT
GGCCAGCAAGTCCCCTGAGGCTTGCGGGGGCCGGGCATACGAGCGGGCCAGCGGGTCACCCCTCAACTGTGGCGTGGGGC
TGCCCACCAGCTTCACCGTAGGCCAGTACTTTGCGGCCCCGTGGAACAGTGTGCTGGTGACACCCACCAGCGACTGCTACAA
CCCAGCGGCGGCGGTGGTGGTCACGGAGCTGGGGCCGGGGGCAGCCCGGGAGCTGGCTGGGCCCCCTGCAGATGCCCTCT
CGGGCCTGCCCAGCAAGAGTGTGTGCAACACATCGGTGCTGAGCAGCAGCCTGCAGTCACTGGAGTATCTCATCAACGACATC
CGGCCGCCCTGCATCAAGGAGCAGATGCTGGGCAAGGGCTATGAGACGGTGGCCGTGCCCCGGCTACTCGACCACCAGCAT
GCCCACATCCGCCTACCCGTCTACAGATAA

4xFlag-TST-Aggregatin (101-452)

ATGAGATCTGACTACAAGGACGACGACGACAAGGGATCTGATTATAAAGATGACGATGATAAGGGATCTGACTACAAAGACGAT
GATGACAAAGGATCTGATTACAAGGATGATGACGATAAAGGATCTGCATGGAGCCATCCCCAGTTTGAGAAAGGTGGCGGATC
AGGCGGGGGTTCTGGTGGATCTGCATGGAGCCATCCCCAGTTTGAGAAAGGTGGCGGATCAGGCGGGGGTTCTGGTGGATCC
GGCCTTCTGGCCATTGTCAAGGCCGCGGTTTCCTCCTCCAGCACGGCCGCACCAGCTGGGCCCGCCAAAAGTGTGCTCAAGA
GCGCCGAGGGCAAGCGGACCAAGCTGTCACCGGCCGCCGTGCAGGTGGGCATTGCGCCCTACCCAGTGCCCAGCACTCTGG
GTCCCTTGGCCTACCCCAAGCCACCTGAGGCGCCTGCTCCACCACCCGGCCTGCCCGCAGCCGCCACTGCCGCCTCCGTCAT
CCCCCTGCCGGGCCGGGGCCTGCCCCTGCCACCTTCCAACCTGCCCTCCATCCACAGCCTCCTGTACCAGCTCAACCAGCAG
TGCCAGGCCCCGGGCGCCGCACCCCCTGCCTGCCAGGGCATGGCTATTCCCCATCCCAGCCCTGCCAAGCACGGCCCAGTG
CCCAGCTTCCCCAGCATGGCCTACTCGGCTGCAGCCGGTCTGCCCGACTGCCGGAAAGGCACTGAGCTGGGCCAGGGAGCC
ACCCAAGCCTTGACGTTGGCTGGGGCCGCCAAGCCTGCAGGGTACGCAGACAGCGGCCTGGATTACCTGCTGTGGCCGCAG
AAACCGCCCCCACCGCCGCCCCAGCCACTGCGTGCCTACAGTGGGAGCACGGTGGCCAGCAAGTCCCCTGAGGCTTGCGGG
GGCCGGGCATACGAGCGGGCCAGCGGGTCACCCCTCAACTGTGGCGTGGGGCTGCCCACCAGCTTCACCGTAGGCCAGTAC
TTTGCGGCCCCGTGGAACAGTGTGCTGGTGACACCCACCAGCGACTGCTACAACCCAGCGGCGGCGGTGGTGGTCACGGAG
CTGGGGCCGGGGGCAGCCCGGGAGCTGGCTGGGCCCCCTGCAGATGCCCTCTCGGGCCTGCCCAGCAAGAGTGTGTGCAA
CACATCGGTGCTGAGCAGCAGCCTGCAGTCACTGGAGTATCTCATCAACGACATCCGGCCGCCCTGCATCAAGGAGCAGATG
CTGGGCAAGGGCTATGAGACGGTGGCCGTGCCCCGGCTACTCGACCACCAGCATGCCCACATCCGCCTACCCGTCTACAGAT
AA

4xFlag-TST-Aggregatin (151-300)

ATGAGATCTGACTACAAGGACGACGACGACAAGGGATCTGATTATAAAGATGACGATGATAAGGGATCTGACTACAAAGACGAT

GATGACAAAGGATCTGATTACAAGGATGATGACGATAAAGGATCTGCATGGAGCCATCCCCAGTTTGAGAAAGGTGGCGGATC
AGGCGGGGGTTCTGGTGGATCTGCATGGAGCCATCCCCAGTTTGAGAAAGGTGGCGGATCAGGCGGGGGTTCTGGTGGATCC
CCCAGCACTCTGGGTCCCTTGGCCTACCCCAAGCCACCTGAGGCGCCTGCTCCACCACCCGGCCTGCCCGCAGCCGCCACTG
CCGCCTCCGTCATCCCCCTGCCGGGCCGGGGCCTGCCCCTGCCACCTTCCAACCTGCCCTCCATCCACAGCCTCCTGTACCA
GCTCAACCAGCAGTGCCAGGCCCCGGGCGCCGCACCCCCTGCCTGCCAGGGCATGGCTATTCCCCATCCCAGCCCTGCCAA
GCACGGCCCAGTGCCCAGCTTCCCCAGCATGGCCTACTCGGCTGCAGCCGGTCTGCCCGACTGCCGGAAAGGCACTGAGCT
GGGCCAGGGAGCCACCCAAGCCTTGACGTTGGCTGGGGCCGCCAAGCCTGCAGGGTACGCAGACAGCGGCCTGGATTACCT
GCTGTGGCCGCAGAAACCGCCCCCACCGCCGCCCCAGCCACTGTAA

4xFlag-TST-Aggregatin (301-452)

ATGAGATCTGACTACAAGGACGACGACGACAAGGGATCTGATTATAAAGATGACGATGATAAGGGATCTGACTACAAAGACGAT
GATGACAAAGGATCTGATTACAAGGATGATGACGATAAAGGATCTGCATGGAGCCATCCCCAGTTTGAGAAAGGTGGCGGATC
AGGCGGGGGTTCTGGTGGATCTGCATGGAGCCATCCCCAGTTTGAGAAAGGTGGCGGATCAGGCGGGGGTTCTGGTGGATCC
CGTGCCTACAGTGGGAGCACGGTGGCCAGCAAGTCCCCTGAGGCTTGCGGGGGCCGGGCATACGAGCGGGCCAGCGGGTC
ACCCCTCAACTGTGGCGTGGGGCTGCCCACCAGCTTCACCGTAGGCCAGTACTTTGCGGCCCCGTGGAACAGTGTGCTGGTG
ACACCCACCAGCGACTGCTACAACCCAGCGGCGGCGGTGGTGGTCACGGAGCTGGGGCCGGGGGCAGCCCGGGAGCTGGC
TGGGCCCCCTGCAGATGCCCTCTCGGGCCTGCCCAGCAAGAGTGTGTGCAACACATCGGTGCTGAGCAGCAGCCTGCAGTCA
CTGGAGTATCTCATCAACGACATCCGGCCGCCCTGCATCAAGGAGCAGATGCTGGGCAAGGGCTATGAGACGGTGGCCGTGC
CCCGGCTACTCGACCACCAGCATGCCCACATCCGCCTACCCGTCTACAGATAA

4xFlag-TST-Aggregatin (61-70)

ATGAGATCTGACTACAAGGACGACGACGACAAGGGATCTGATTATAAAGATGACGATGATAAGGGATCTGACTACAAAGACGAT
GATGACAAAGGATCTGATTACAAGGATGATGACGATAAAGGATCTGCATGGAGCCATCCCCAGTTTGAGAAAGGTGGCGGATC
AGGCGGGGGTTCTGGTGGATCTGCATGGAGCCATCCCCAGTTTGAGAAAGGTGGCGGATCAGGCGGGGGTTCTGGTGGATCC
AAGATCTTCCCCACCAACATCCGTGTGCCCTAA

4xFlag-TST-Aggregatin (66-75)

ATGAGATCTGACTACAAGGACGACGACGACAAGGGATCTGATTATAAAGATGACGATGATAAGGGATCTGACTACAAAGACGAT
GATGACAAAGGATCTGATTACAAGGATGATGACGATAAAGGATCTGCATGGAGCCATCCCCAGTTTGAGAAAGGTGGCGGATC
AGGCGGGGGTTCTGGTGGATCTGCATGGAGCCATCCCCAGTTTGAGAAAGGTGGCGGATCAGGCGGGGGTTCTGGTGGATCC
AACATCCGTGTGCCCCAGCACAAGCACCTCTAA

4xFlag-TST-Aggregatin (71-80)

ATGAGATCTGACTACAAGGACGACGACGACAAGGGATCTGATTATAAAGATGACGATGATAAGGGATCTGACTACAAAGACGAT
GATGACAAAGGATCTGATTACAAGGATGATGACGATAAAGGATCTGCATGGAGCCATCCCCAGTTTGAGAAAGGTGGCGGATC
AGGCGGGGGTTCTGGTGGATCTGCATGGAGCCATCCCCAGTTTGAGAAAGGTGGCGGATCAGGCGGGGGTTCTGGTGGATCC
CAGCACAAGCACCTCAGCCGCACAGTCAATTAA

4xFlag-TST-Aggregatin (56-75)

ATGAGATCTGACTACAAGGACGACGACGACAAGGGATCTGATTATAAAGATGACGATGATAAGGGATCTGACTACAAAGACGAT
GATGACAAAGGATCTGATTACAAGGATGATGACGATAAAGGATCTGCATGGAGCCATCCCCAGTTTGAGAAAGGTGGCGGATC
AGGCGGGGGTTCTGGTGGATCTGCATGGAGCCATCCCCAGTTTGAGAAAGGTGGCGGATCAGGCGGGGGTTCTGGTGGATCC
AGCCCGCTGTCCATCAAGATCTTCCCCACCAACATCCGTGTGCCCCAGCACAAGCACCTCTAA

4xFlag-TST-Aggregatin (61-80)

ATGAGATCTGACTACAAGGACGACGACGACAAGGGATCTGATTATAAAGATGACGATGATAAGGGATCTGACTACAAAGACGAT
GATGACAAAGGATCTGATTACAAGGATGATGACGATAAAGGATCTGCATGGAGCCATCCCCAGTTTGAGAAAGGTGGCGGATC
AGGCGGGGGTTCTGGTGGATCTGCATGGAGCCATCCCCAGTTTGAGAAAGGTGGCGGATCAGGCGGGGGTTCTGGTGGATCC
AAGATCTTCCCCACCAACATCCGTGTGCCCCAGCACAAGCACCTCAGCCGCACAGTCAATTAA

4xFlag-TST-Aggregatin (41-80)

ATGAGATCTGACTACAAGGACGACGACGACAAGGGATCTGATTATAAAGATGACGATGATAAGGGATCTGACTACAAAGACGAT
GATGACAAAGGATCTGATTACAAGGATGATGACGATAAAGGATCTGCATGGAGCCATCCCCAGTTTGAGAAAGGTGGCGGATC
AGGCGGGGGTTCTGGTGGATCTGCATGGAGCCATCCCCAGTTTGAGAAAGGTGGCGGATCAGGCGGGGGTTCTGGTGGATCC
CCGAGCCCAGCAGAACTGGACGCCTATGCCGAGAAGGTGGCCAACAGCCCGCTGTCCATCAAGATCTTCCCCACCAACATCC
GTGTGCCCCAGCACAAGCACCTCAGCCGCACAGTCAATTAA

4xFlag-TST-Aggregatin (51-80)

ATGAGATCTGACTACAAGGACGACGACGACAAGGGATCTGATTATAAAGATGACGATGATAAGGGATCTGACTACAAAGACGAT
GATGACAAAGGATCTGATTACAAGGATGATGACGATAAAGGATCTGCATGGAGCCATCCCCAGTTTGAGAAAGGTGGCGGATC
AGGCGGGGGTTCTGGTGGATCTGCATGGAGCCATCCCCAGTTTGAGAAAGGTGGCGGATCAGGCGGGGGTTCTGGTGGATCC
GAGAAGGTGGCCAACAGCCCGCTGTCCATCAAGATCTTCCCCACCAACATCCGTGTGCCCCAGCACAAGCACCTCAGCCGCA
CAGTCAATTAA

4xFlag-TST-Aggregatin (21-80)

ATGAGATCTGACTACAAGGACGACGACGACAAGGGATCTGATTATAAAGATGACGATGATAAGGGATCTGACTACAAAGACGAT
GATGACAAAGGATCTGATTACAAGGATGATGACGATAAAGGATCTGCATGGAGCCATCCCCAGTTTGAGAAAGGTGGCGGATC
AGGCGGGGGTTCTGGTGGATCTGCATGGAGCCATCCCCAGTTTGAGAAAGGTGGCGGATCAGGCGGGGGTTCTGGTGGATCC
AAGAGCCTGGAGCTGCGCAAGTGCGAGGCGGTGGCCAGCGCCATGCATTCCTCCCGCTACCCGAGCCCAGCAGAACTGGAC

GCCTATGCCGAGAAGGTGGCCAACAGCCCGCTGTCCATCAAGATCTTCCCCACCAACATCCGTGTGCCCCAGCACAAGCACCT
CAGCCGCACAGTCAATTAA

4xFlag-TST-Aggregatin (31-80)

ATGAGATCTGACTACAAGGACGACGACGACAAGGGATCTGATTATAAAGATGACGATGATAAGGGATCTGACTACAAAGACGAT
GATGACAAAGGATCTGATTACAAGGATGATGACGATAAAGGATCTGCATGGAGCCATCCCCAGTTTGAGAAAGGTGGCGGATC
AGGCGGGGGTTCTGGTGGATCTGCATGGAGCCATCCCCAGTTTGAGAAAGGTGGCGGATCAGGCGGGGGTTCTGGTGGATCC
GTGGCCAGCGCCATGCATTCCTCCCGCTACCCGAGCCCAGCAGAACTGGACGCCTATGCCGAGAAGGTGGCCAACAGCCCGC
TGTCCATCAAGATCTTCCCCACCAACATCCGTGTGCCCCAGCACAAGCACCTCAGCCGCACAGTCAATTAA

4xFlag-TST-Aggregatin (81-452)

ATGAGATCTGACTACAAGGACGACGACGACAAGGGATCTGATTATAAAGATGACGATGATAAGGGATCTGACTACAAAGACGAT
GATGACAAAGGATCTGATTACAAGGATGATGACGATAAAGGATCTGCATGGAGCCATCCCCAGTTTGAGAAAGGTGGCGGATC
AGGCGGGGGTTCTGGTGGATCTGCATGGAGCCATCCCCAGTTTGAGAAAGGTGGCGGATCAGGCGGGGGTTCTGGTGGATCC
GGCTATGACACCAGTGGCCAGCGCTACAGCCCCTACCCACAGCACACCGCTGGCTACCAGGGCCTTCTGGCCATTGTCAAGG
CCGCGGTTTCCTCCTCCAGCACGGCCGCACCAGCTGGGCCCGCCAAAAGTGTGCTCAAGAGCGCCGAGGGCAAGCGGACCA
AGCTGTCACCGGCCGCCGTGCAGGTGGGCATTGCGCCCTACCCAGTGCCCAGCACTCTGGGTCCCTTGGCCTACCCCAAGCC
ACCTGAGGCGCCTGCTCCACCACCCGGCCTGCCCGCAGCCGCCACTGCCGCCTCCGTCATCCCCCTGCCGGGCCGGGGCCT
GCCCCTGCCACCTTCCAACCTGCCCTCCATCCACAGCCTCCTGTACCAGCTCAACCAGCAGTGCCAGGCCCCGGGCGCCGCA
CCCCCTGCCTGCCAGGGCATGGCTATTCCCCATCCCAGCCCTGCCAAGCACGGCCCAGTGCCCAGCTTCCCCAGCATGGCCT
ACTCGGCTGCAGCCGGTCTGCCCGACTGCCGGAAAGGCACTGAGCTGGGCCAGGGAGCCACCCAAGCCTTGACGTTGGCTG
GGGCCGCCAAGCCTGCAGGGTACGCAGACAGCGGCCTGGATTACCTGCTGTGGCCGCAGAAACCGCCCCCACCGCCGCCCC
AGCCACTGCGTGCCTACAGTGGGAGCACGGTGGCCAGCAAGTCCCCTGAGGCTTGCGGGGGCCGGGCATACGAGCGGGCCA
GCGGGTCACCCCTCAACTGTGGCGTGGGGCTGCCCACCAGCTTCACCGTAGGCCAGTACTTTGCGGCCCCGTGGAACAGTGT
GCTGGTGACACCCACCAGCGACTGCTACAACCCAGCGGCGGCGGTGGTGGTCACGGAGCTGGGGCCGGGGGCAGCCCGGG
AGCTGGCTGGGCCCCCTGCAGATGCCCTCTCGGGCCTGCCCAGCAAGAGTGTGTGCAACACATCGGTGCTGAGCAGCAGCCT
GCAGTCACTGGAGTATCTCATCAACGACATCCGGCCGCCCTGCATCAAGGAGCAGATGCTGGGCAAGGGCTATGAGACGGTG
GCCGTGCCCCGGCTACTCGACCACCAGCATGCCCACATCCGCCTACCCGTCTACAGATAA

4xFlag-TST-Aggregatin (Δ61-80)

ATGAGATCTGACTACAAGGACGACGACGACAAGGGATCTGATTATAAAGATGACGATGATAAGGGATCTGACTACAAAGACGAT
GATGACAAAGGATCTGATTACAAGGATGATGACGATAAAGGATCTGCATGGAGCCATCCCCAGTTTGAGAAAGGTGGCGGATC
AGGCGGGGGTTCTGGTGGATCTGCATGGAGCCATCCCCAGTTTGAGAAAGGTGGCGGATCAGGCGGGGGTTCTGGTGGATCC
ATGCTGGCCTGTCTGCAGAGGACCCAGAACGCCCCGGGCCAACACCTGGCCTGCCCGAGCAAGAGCCTGGAGCTGCGCAAG
TGCGAGGCGGTGGCCAGCGCCATGCATTCCTCCCGCTACCCGAGCCCAGCAGAACTGGACGCCTATGCCGAGAAGGTGGCC
AACAGCCCGCTGTCCATCGGCTATGACACCAGTGGCCAGCGCTACAGCCCCTACCCACAGCACACCGCTGGCTACCAGGGCC
TTCTGGCCATTGTCAAGGCCGCGGTTTCCTCCTCCAGCACGGCCGCACCAGCTGGGCCCGCCAAAAGTGTGCTCAAGAGCGC
CGAGGGCAAGCGGACCAAGCTGTCACCGGCCGCCGTGCAGGTGGGCATTGCGCCCTACCCAGTGCCCAGCACTCTGGGTCC
CTTGGCCTACCCCAAGCCACCTGAGGCGCCTGCTCCACCACCCGGCCTGCCCGCAGCCGCCACTGCCGCCTCCGTCATCCCC
CTGCCGGGCCGGGGCCTGCCCCTGCCACCTTCCAACCTGCCCTCCATCCACAGCCTCCTGTACCAGCTCAACCAGCAGTGCC
AGGCCCCGGGCGCCGCACCCCCTGCCTGCCAGGGCATGGCTATTCCCCATCCCAGCCCTGCCAAGCACGGCCCAGTGCCCA
GCTTCCCCAGCATGGCCTACTCGGCTGCAGCCGGTCTGCCCGACTGCCGGAAAGGCACTGAGCTGGGCCAGGGAGCCACCC
AAGCCTTGACGTTGGCTGGGGCCGCCAAGCCTGCAGGGTACGCAGACAGCGGCCTGGATTACCTGCTGTGGCCGCAGAAACC
GCCCCCACCGCCGCCCCAGCCACTGCGTGCCTACAGTGGGAGCACGGTGGCCAGCAAGTCCCCTGAGGCTTGCGGGGGCCG
GGCATACGAGCGGGCCAGCGGGTCACCCCTCAACTGTGGCGTGGGGCTGCCCACCAGCTTCACCGTAGGCCAGTACTTTGCG
GCCCCGTGGAACAGTGTGCTGGTGACACCCACCAGCGACTGCTACAACCCAGCGGCGGCGGTGGTGGTCACGGAGCTGGGG
CCGGGGGCAGCCCGGGAGCTGGCTGGGCCCCCTGCAGATGCCCTCTCGGGCCTGCCCAGCAAGAGTGTGTGCAACACATCG
GTGCTGAGCAGCAGCCTGCAGTCACTGGAGTATCTCATCAACGACATCCGGCCGCCCTGCATCAAGGAGCAGATGCTGGGCA
AGGGCTATGAGACGGTGGCCGTGCCCCGGCTACTCGACCACCAGCATGCCCACATCCGCCTACCCGTCTACAGATAA

4xFlag-TST-Aggregatin (Δ81-180)

ATGAGATCTGACTACAAGGACGACGACGACAAGGGATCTGATTATAAAGATGACGATGATAAGGGATCTGACTACAAAGACGAT
GATGACAAAGGATCTGATTACAAGGATGATGACGATAAAGGATCTGCATGGAGCCATCCCCAGTTTGAGAAAGGTGGCGGATC
AGGCGGGGGTTCTGGTGGATCTGCATGGAGCCATCCCCAGTTTGAGAAAGGTGGCGGATCAGGCGGGGGTTCTGGTGGATCC
ATGCTGGCCTGTCTGCAGAGGACCCAGAACGCCCCGGGCCAACACCTGGCCTGCCCGAGCAAGAGCCTGGAGCTGCGCAAG
TGCGAGGCGGTGGCCAGCGCCATGCATTCCTCCCGCTACCCGAGCCCAGCAGAACTGGACGCCTATGCCGAGAAGGTGGCC
AACAGCCCGCTGTCCATCAAGATCTTCCCCACCAACATCCGTGTGCCCCAGCACAAGCACCTCAGCCGCACAGTCAATGTCAT
CCCCCTGCCGGGCCGGGGCCTGCCCCTGCCACCTTCCAACCTGCCCTCCATCCACAGCCTCCTGTACCAGCTCAACCAGCAG
TGCCAGGCCCCGGGCGCCGCACCCCCTGCCTGCCAGGGCATGGCTATTCCCCATCCCAGCCCTGCCAAGCACGGCCCAGTG
CCCAGCTTCCCCAGCATGGCCTACTCGGCTGCAGCCGGTCTGCCCGACTGCCGGAAAGGCACTGAGCTGGGCCAGGGAGCC
ACCCAAGCCTTGACGTTGGCTGGGGCCGCCAAGCCTGCAGGGTACGCAGACAGCGGCCTGGATTACCTGCTGTGGCCGCAG
AAACCGCCCCCACCGCCGCCCCAGCCACTGCGTGCCTACAGTGGGAGCACGGTGGCCAGCAAGTCCCCTGAGGCTTGCGGG
GGCCGGGCATACGAGCGGGCCAGCGGGTCACCCCTCAACTGTGGCGTGGGGCTGCCCACCAGCTTCACCGTAGGCCAGTAC
TTTGCGGCCCCGTGGAACAGTGTGCTGGTGACACCCACCAGCGACTGCTACAACCCAGCGGCGGCGGTGGTGGTCACGGAG
CTGGGGCCGGGGGCAGCCCGGGAGCTGGCTGGGCCCCCTGCAGATGCCCTCTCGGGCCTGCCCAGCAAGAGTGTGTGCAA
CACATCGGTGCTGAGCAGCAGCCTGCAGTCACTGGAGTATCTCATCAACGACATCCGGCCGCCCTGCATCAAGGAGCAGATG
CTGGGCAAGGGCTATGAGACGGTGGCCGTGCCCCGGCTACTCGACCACCAGCATGCCCACATCCGCCTACCCGTCTACAGAT
AA

4xFlag-TST-Aggregatin (Δ171-270)
ATGAGATCTGACTACAAGGACGACGACGACAAGGGATCTGATTATAAAGATGACGATGATAAGGGATCTGACTACAAAGACGAT
GATGACAAAGGATCTGATTACAAGGATGATGACGATAAAGGATCTGCATGGAGCCATCCCCAGTTTGAGAAAGGTGGCGGATC
AGGCGGGGGTTCTGGTGGATCTGCATGGAGCCATCCCCAGTTTGAGAAAGGTGGCGGATCAGGCGGGGGTTCTGGTGGATCC
ATGCTGGCCTGTCTGCAGAGGACCCAGAACGCCCCGGGCCAACACCTGGCCTGCCCGAGCAAGAGCCTGGAGCTGCGCAAG
TGCGAGGCGGTGGCCAGCGCCATGCATTCCTCCCGCTACCCGAGCCCAGCAGAACTGGACGCCTATGCCGAGAAGGTGGCC
AACAGCCCGCTGTCCATCAAGATCTTCCCCACCAACATCCGTGTGCCCCAGCACAAGCACCTCAGCCGCACAGTCAATGGCTA
TGACACCAGTGGCCAGCGCTACAGCCCCTACCCACAGCACACCGCTGGCTACCAGGGCCTTCTGGCCATTGTCAAGGCCGCG
GTTTCCTCCTCCAGCACGGCCGCACCAGCTGGGCCCGCCAAAAGTGTGCTCAAGAGCGCCGAGGGCAAGCGGACCAAGCTGT
CACCGGCCGCCGTGCAGGTGGGCATTGCGCCCTACCCAGTGCCCAGCACTCTGGGTCCCTTGGCCTACCCCAAGCCACCTGA
GGCGCCTGCTCCACCACCCGGGGCCGCCAAGCCTGCAGGGTACGCAGACAGCGGCCTGGATTACCTGCTGTGGCCGCAGAA
ACCGCCCCCACCGCCGCCCCAGCCACTGCGTGCCTACAGTGGGAGCACGGTGGCCAGCAAGTCCCCTGAGGCTTGCGGGGG
CCGGGCATACGAGCGGGCCAGCGGGTCACCCCTCAACTGTGGCGTGGGGCTGCCCACCAGCTTCACCGTAGGCCAGTACTTT
GCGGCCCCGTGGAACAGTGTGCTGGTGACACCCACCAGCGACTGCTACAACCCAGCGGCGGCGGTGGTGGTCACGGAGCTG
GGGCCGGGGGCAGCCCGGGAGCTGGCTGGGCCCCCTGCAGATGCCCTCTCGGGCCTGCCCAGCAAGAGTGTGTGCAACACA
TCGGTGCTGAGCAGCAGCCTGCAGTCACTGGAGTATCTCATCAACGACATCCGGCCGCCCTGCATCAAGGAGCAGATGCTGG
GCAAGGGCTATGAGACGGTGGCCGTGCCCCGGCTACTCGACCACCAGCATGCCCACATCCGCCTACCCGTCTACAGATAA

4xFlag-TST-Aggregatin (Δ261-360)
ATGAGATCTGACTACAAGGACGACGACGACAAGGGATCTGATTATAAAGATGACGATGATAAGGGATCTGACTACAAAGACGAT
GATGACAAAGGATCTGATTACAAGGATGATGACGATAAAGGATCTGCATGGAGCCATCCCCAGTTTGAGAAAGGTGGCGGATC
AGGCGGGGGTTCTGGTGGATCTGCATGGAGCCATCCCCAGTTTGAGAAAGGTGGCGGATCAGGCGGGGGTTCTGGTGGATCC
ATGCTGGCCTGTCTGCAGAGGACCCAGAACGCCCCGGGCCAACACCTGGCCTGCCCGAGCAAGAGCCTGGAGCTGCGCAAG
TGCGAGGCGGTGGCCAGCGCCATGCATTCCTCCCGCTACCCGAGCCCAGCAGAACTGGACGCCTATGCCGAGAAGGTGGCC
AACAGCCCGCTGTCCATCAAGATCTTCCCCACCAACATCCGTGTGCCCCAGCACAAGCACCTCAGCCGCACAGTCAATGGCTA
TGACACCAGTGGCCAGCGCTACAGCCCCTACCCACAGCACACCGCTGGCTACCAGGGCCTTCTGGCCATTGTCAAGGCCGCG
GTTTCCTCCTCCAGCACGGCCGCACCAGCTGGGCCCGCCAAAAGTGTGCTCAAGAGCGCCGAGGGCAAGCGGACCAAGCTGT
CACCGGCCGCCGTGCAGGTGGGCATTGCGCCCTACCCAGTGCCCAGCACTCTGGGTCCCTTGGCCTACCCCAAGCCACCTGA
GGCGCCTGCTCCACCACCCGGCCTGCCCGCAGCCGCCACTGCCGCCTCCGTCATCCCCCTGCCGGGCCGGGGCCTGCCCCT
GCCACCTTCCAACCTGCCCTCCATCCACAGCCTCCTGTACCAGCTCAACCAGCAGTGCCAGGCCCCGGGCGCCGCACCCCCT
GCCTGCCAGGGCATGGCTATTCCCCATCCCAGCCCTGCCAAGCACGGCCCAGTGCCCAGCTTCCCCAGCATGGCCTACTCGG
CTGCAGCCGGTCTGCCCGACTGCCGGAAAGGCACTGAGCTGGGCTACAACCCAGCGGCGGCGGTGGTGGTCACGGAGCTGG
GGCCGGGGGCAGCCCGGGAGCTGGCTGGGCCCCCTGCAGATGCCCTCTCGGGCCTGCCCAGCAAGAGTGTGTGCAACACAT
CGGTGCTGAGCAGCAGCCTGCAGTCACTGGAGTATCTCATCAACGACATCCGGCCGCCCTGCATCAAGGAGCAGATGCTGGG
CAAGGGCTATGAGACGGTGGCCGTGCCCCGGCTACTCGACCACCAGCATGCCCACATCCGCCTACCCGTCTACAGATAA

4xFlag-TST-Aggregatin (Δ61-65)
ATGAGATCTGACTACAAGGACGACGACGACAAGGGATCTGATTATAAAGATGACGATGATAAGGGATCTGACTACAAAGACGAT
GATGACAAAGGATCTGATTACAAGGATGATGACGATAAAGGATCTGCATGGAGCCATCCCCAGTTTGAGAAAGGTGGCGGATC
AGGCGGGGGTTCTGGTGGATCTGCATGGAGCCATCCCCAGTTTGAGAAAGGTGGCGGATCAGGCGGGGGTTCTGGTGGATCC
ATGCTGGCCTGTCTGCAGAGGACCCAGAACGCCCCGGGCCAACACCTGGCCTGCCCGAGCAAGAGCCTGGAGCTGCGCAAG
TGCGAGGCGGTGGCCAGCGCCATGCATTCCTCCCGCTACCCGAGCCCAGCAGAACTGGACGCCTATGCCGAGAAGGTGGCC
AACAGCCCGCTGTCCATCAACATCCGTGTGCCCCAGCACAAGCACCTCAGCCGCACAGTCAATGGCTATGACACCAGTGGCCA
GCGCTACAGCCCCTACCCACAGCACACCGCTGGCTACCAGGGCCTTCTGGCCATTGTCAAGGCCGCGGTTTCCTCCTCCAGC
ACGGCCGCACCAGCTGGGCCCGCCAAAAGTGTGCTCAAGAGCGCCGAGGGCAAGCGGACCAAGCTGTCACCGGCCGCCGTG
CAGGTGGGCATTGCGCCCTACCCAGTGCCCAGCACTCTGGGTCCCTTGGCCTACCCCAAGCCACCTGAGGCGCCTGCTCCAC
CACCCGGCCTGCCCGCAGCCGCCACTGCCGCCTCCGTCATCCCCCTGCCGGGCCGGGGCCTGCCCCTGCCACCTTCCAACC
TGCCCTCCATCCACAGCCTCCTGTACCAGCTCAACCAGCAGTGCCAGGCCCCGGGCGCCGCACCCCCTGCCTGCCAGGGCAT
GGCTATTCCCCATCCCAGCCCTGCCAAGCACGGCCCAGTGCCCAGCTTCCCCAGCATGGCCTACTCGGCTGCAGCCGGTCTG
CCCGACTGCCGGAAAGGCACTGAGCTGGGCCAGGGAGCCACCCAAGCCTTGACGTTGGCTGGGGCCGCCAAGCCTGCAGGG
TACGCAGACAGCGGCCTGGATTACCTGCTGTGGCCGCAGAAACCGCCCCCACCGCCGCCCCAGCCACTGCGTGCCTACAGTG
GGAGCACGGTGGCCAGCAAGTCCCCTGAGGCTTGCGGGGGCCGGGCATACGAGCGGGCCAGCGGGTCACCCCTCAACTGTG
GCGTGGGGCTGCCCACCAGCTTCACCGTAGGCCAGTACTTTGCGGCCCCGTGGAACAGTGTGCTGGTGACACCCACCAGCGA
CTGCTACAACCCAGCGGCGGCGGTGGTGGTCACGGAGCTGGGGCCGGGGGCAGCCCGGGAGCTGGCTGGGCCCCCTGCAG
ATGCCCTCTCGGGCCTGCCCAGCAAGAGTGTGTGCAACACATCGGTGCTGAGCAGCAGCCTGCAGTCACTGGAGTATCTCATC
AACGACATCCGGCCGCCCTGCATCAAGGAGCAGATGCTGGGCAAGGGCTATGAGACGGTGGCCGTGCCCCGGCTACTCGAC
CACCAGCATGCCCACATCCGCCTACCCGTCTACAGATAA

4xFlag-TST-Aggregatin (Δ66-70)
ATGAGATCTGACTACAAGGACGACGACGACAAGGGATCTGATTATAAAGATGACGATGATAAGGGATCTGACTACAAAGACGAT
GATGACAAAGGATCTGATTACAAGGATGATGACGATAAAGGATCTGCATGGAGCCATCCCCAGTTTGAGAAAGGTGGCGGATC
AGGCGGGGGTTCTGGTGGATCTGCATGGAGCCATCCCCAGTTTGAGAAAGGTGGCGGATCAGGCGGGGGTTCTGGTGGATCC
ATGCTGGCCTGTCTGCAGAGGACCCAGAACGCCCCGGGCCAACACCTGGCCTGCCCGAGCAAGAGCCTGGAGCTGCGCAAG
TGCGAGGCGGTGGCCAGCGCCATGCATTCCTCCCGCTACCCGAGCCCAGCAGAACTGGACGCCTATGCCGAGAAGGTGGCC
AACAGCCCGCTGTCCATCAAGATCTTCCCCACCCAGCACAAGCACCTCAGCCGCACAGTCAATGGCTATGACACCAGTGGCCA
GCGCTACAGCCCCTACCCACAGCACACCGCTGGCTACCAGGGCCTTCTGGCCATTGTCAAGGCCGCGGTTTCCTCCTCCAGC
ACGGCCGCACCAGCTGGGCCCGCCAAAAGTGTGCTCAAGAGCGCCGAGGGCAAGCGGACCAAGCTGTCACCGGCCGCCGTG
CAGGTGGGCATTGCGCCCTACCCAGTGCCCAGCACTCTGGGTCCCTTGGCCTACCCCAAGCCACCTGAGGCGCCTGCTCCAC
CACCCGGCCTGCCCGCAGCCGCCACTGCCGCCTCCGTCATCCCCCTGCCGGGCCGGGGCCTGCCCCTGCCACCTTCCAACC

TGCCCTCCATCCACAGCCTCCTGTACCAGCTCAACCAGCAGTGCCAGGCCCCGGGCGCCGCACCCCCTGCCTGCCAGGGCAT
GGCTATTCCCCATCCCAGCCCTGCCAAGCACGGCCCAGTGCCCAGCTTCCCCAGCATGGCCTACTCGGCTGCAGCCGGTCTG
CCCGACTGCCGGAAAGGCACTGAGCTGGGCCAGGGAGCCACCCAAGCCTTGACGTTGGCTGGGGCCGCCAAGCCTGCAGGG
TACGCAGACAGCGGCCTGGATTACCTGCTGTGGCCGCAGAAACCGCCCCCACCGCCGCCCCAGCCACTGCGTGCCTACAGTG
GGAGCACGGTGGCCAGCAAGTCCCCTGAGGCTTGCGGGGGCCGGGCATACGAGCGGGCCAGCGGGTCACCCCTCAACTGTG
GCGTGGGGCTGCCCACCAGCTTCACCGTAGGCCAGTACTTTGCGGCCCCGTGGAACAGTGTGCTGGTGACACCCACCAGCGA
CTGCTACAACCCAGCGGCGGCGGTGGTGGTCACGGAGCTGGGGCCGGGGGCAGCCCGGGAGCTGGCTGGGCCCCCTGCAG
ATGCCCTCTCGGGCCTGCCCAGCAAGAGTGTGTGCAACACATCGGTGCTGAGCAGCAGCCTGCAGTCACTGGAGTATCTCATC
AACGACATCCGGCCGCCCTGCATCAAGGAGCAGATGCTGGGCAAGGGCTATGAGACGGTGGCCGTGCCCCGGCTACTCGAC
CACCAGCATGCCCACATCCGCCTACCCGTCTACAGATAA

4xFlag-TST-Aggregatin (Δ71-75)

ATGAGATCTGACTACAAGGACGACGACGACAAGGGATCTGATTATAAAGATGACGATGATAAGGGATCTGACTACAAAGACGAT
GATGACAAAGGATCTGATTACAAGGATGATGACGATAAAGGATCTGCATGGAGCCATCCCCAGTTTGAGAAAGGTGGCGGATC
AGGCGGGGGTTCTGGTGGATCTGCATGGAGCCATCCCCAGTTTGAGAAAGGTGGCGGATCAGGCGGGGGTTCTGGTGGATCC
ATGCTGGCCTGTCTGCAGAGGACCCAGAACGCCCCGGGCCAACACCTGGCCTGCCCGAGCAAGAGCCTGGAGCTGCGCAAG
TGCGAGGCGGTGGCCAGCGCCATGCATTCCTCCCGCTACCCGAGCCCAGCAGAACTGGACGCCTATGCCGAGAAGGTGGCC
AACAGCCCGCTGTCCATCAAGATCTTCCCCACCAACATCCGTGTGCCCAGCCGCACAGTCAATGGCTATGACACCAGTGGCCA
GCGCTACAGCCCCTACCCACAGCACACCGCTGGCTACCAGGGCCTTCTGGCCATTGTCAAGGCCGCGGTTTCCTCCTCCAGC
ACGGCCGCACCAGCTGGGCCCGCCAAAAGTGTGCTCAAGAGCGCCGAGGGCAAGCGGACCAAGCTGTCACCGGCCGCCGTG
CAGGTGGGCATTGCGCCCTACCCAGTGCCCAGCACTCTGGGTCCCTTGGCCTACCCCAAGCCACCTGAGGCGCCTGCTCCAC
CACCCGGCCTGCCCGCAGCCGCCACTGCCGCCTCCGTCATCCCCCTGCCGGGCCGGGGCCTGCCCCTGCCACCTTCCAACC
TGCCCTCCATCCACAGCCTCCTGTACCAGCTCAACCAGCAGTGCCAGGCCCCGGGCGCCGCACCCCCTGCCTGCCAGGGCAT
GGCTATTCCCCATCCCAGCCCTGCCAAGCACGGCCCAGTGCCCAGCTTCCCCAGCATGGCCTACTCGGCTGCAGCCGGTCTG
CCCGACTGCCGGAAAGGCACTGAGCTGGGCCAGGGAGCCACCCAAGCCTTGACGTTGGCTGGGGCCGCCAAGCCTGCAGGG
TACGCAGACAGCGGCCTGGATTACCTGCTGTGGCCGCAGAAACCGCCCCCACCGCCGCCCCAGCCACTGCGTGCCTACAGTG
GGAGCACGGTGGCCAGCAAGTCCCCTGAGGCTTGCGGGGGCCGGGCATACGAGCGGGCCAGCGGGTCACCCCTCAACTGTG
GCGTGGGGCTGCCCACCAGCTTCACCGTAGGCCAGTACTTTGCGGCCCCGTGGAACAGTGTGCTGGTGACACCCACCAGCGA
CTGCTACAACCCAGCGGCGGCGGTGGTGGTCACGGAGCTGGGGCCGGGGGCAGCCCGGGAGCTGGCTGGGCCCCCTGCAG
ATGCCCTCTCGGGCCTGCCCAGCAAGAGTGTGTGCAACACATCGGTGCTGAGCAGCAGCCTGCAGTCACTGGAGTATCTCATC
AACGACATCCGGCCGCCCTGCATCAAGGAGCAGATGCTGGGCAAGGGCTATGAGACGGTGGCCGTGCCCCGGCTACTCGAC
CACCAGCATGCCCACATCCGCCTACCCGTCTACAGATAA

4xFlag-TST-Aggregatin (Δ76-80)

ATGAGATCTGACTACAAGGACGACGACGACAAGGGATCTGATTATAAAGATGACGATGATAAGGGATCTGACTACAAAGACGAT
GATGACAAAGGATCTGATTACAAGGATGATGACGATAAAGGATCTGCATGGAGCCATCCCCAGTTTGAGAAAGGTGGCGGATC
AGGCGGGGGTTCTGGTGGATCTGCATGGAGCCATCCCCAGTTTGAGAAAGGTGGCGGATCAGGCGGGGGTTCTGGTGGATCC
ATGCTGGCCTGTCTGCAGAGGACCCAGAACGCCCCGGGCCAACACCTGGCCTGCCCGAGCAAGAGCCTGGAGCTGCGCAAG
TGCGAGGCGGTGGCCAGCGCCATGCATTCCTCCCGCTACCCGAGCCCAGCAGAACTGGACGCCTATGCCGAGAAGGTGGCC
AACAGCCCGCTGTCCATCAAGATCTTCCCCACCAACATCCGTGTGCCCCAGCACAAGCACCTCGGCTATGACACCAGTGGCCA
GCGCTACAGCCCCTACCCACAGCACACCGCTGGCTACCAGGGCCTTCTGGCCATTGTCAAGGCCGCGGTTTCCTCCTCCAGC
ACGGCCGCACCAGCTGGGCCCGCCAAAAGTGTGCTCAAGAGCGCCGAGGGCAAGCGGACCAAGCTGTCACCGGCCGCCGTG
CAGGTGGGCATTGCGCCCTACCCAGTGCCCAGCACTCTGGGTCCCTTGGCCTACCCCAAGCCACCTGAGGCGCCTGCTCCAC
CACCCGGCCTGCCCGCAGCCGCCACTGCCGCCTCCGTCATCCCCCTGCCGGGCCGGGGCCTGCCCCTGCCACCTTCCAACC
TGCCCTCCATCCACAGCCTCCTGTACCAGCTCAACCAGCAGTGCCAGGCCCCGGGCGCCGCACCCCCTGCCTGCCAGGGCAT
GGCTATTCCCCATCCCAGCCCTGCCAAGCACGGCCCAGTGCCCAGCTTCCCCAGCATGGCCTACTCGGCTGCAGCCGGTCTG
CCCGACTGCCGGAAAGGCACTGAGCTGGGCCAGGGAGCCACCCAAGCCTTGACGTTGGCTGGGGCCGCCAAGCCTGCAGGG
TACGCAGACAGCGGCCTGGATTACCTGCTGTGGCCGCAGAAACCGCCCCCACCGCCGCCCCAGCCACTGCGTGCCTACAGTG
GGAGCACGGTGGCCAGCAAGTCCCCTGAGGCTTGCGGGGGCCGGGCATACGAGCGGGCCAGCGGGTCACCCCTCAACTGTG
GCGTGGGGCTGCCCACCAGCTTCACCGTAGGCCAGTACTTTGCGGCCCCGTGGAACAGTGTGCTGGTGACACCCACCAGCGA
CTGCTACAACCCAGCGGCGGCGGTGGTGGTCACGGAGCTGGGGCCGGGGGCAGCCCGGGAGCTGGCTGGGCCCCCTGCAG
ATGCCCTCTCGGGCCTGCCCAGCAAGAGTGTGTGCAACACATCGGTGCTGAGCAGCAGCCTGCAGTCACTGGAGTATCTCATC
AACGACATCCGGCCGCCCTGCATCAAGGAGCAGATGCTGGGCAAGGGCTATGAGACGGTGGCCGTGCCCCGGCTACTCGAC
CACCAGCATGCCCACATCCGCCTACCCGTCTACAGATAA

3xMyc-TST-Aggregatin (1-80)

ATGGAGCAGAAACTCATCTCTGAAGAAGATCTGGAACAAAAGTTGATTTCAGAAGAAGATCTGGAACAGAAGCTCATCTCTGAG
GAAGATCTGGCCCGGGCGGGATCTGATGACGATAAAGGATCTGCATGGAGCCATCCCCAGTTTGAGAAAGGTGGCGGATCAG
GCGGGGGTTCTGGTGGATCTGCATGGAGCCATCCCCAGTTTGAGAAAGGTGGCGGATCAGGCGGGGGTTCTGGTGGATCCAT
GCTGGCCTGTCTGCAGAGGACCCAGAACGCCCCGGGCCAACACCTGGCCTGCCCGAGCAAGAGCCTGGAGCTGCGCAAGTG
CGAGGCGGTGGCCAGCGCCATGCATTCCTCCCGCTACCCGAGCCCAGCAGAACTGGACGCCTATGCCGAGAAGGTGGCCAA
CAGCCCGCTGTCCATCAAGATCTTCCCCACCAACATCCGTGTGCCCCAGCACAAGCACCTCAGCCGCACAGTCAATTAA
