# Supplementary material for: FAM222A encodes a protein which accumulates in plaques in Alzheimer’s disease
Source: Nat Commun. 2020 Jan 21;11:411. doi: 10.1038/s41467-019-13962-0 (PMC6972869; doi:10.1038/s41467-019-13962-0)
Supplement: Supplementary file 4 — Reporting Summary [file 41467_2019_13962_MOESM4_ESM.pdf]

nature research

Corresponding author(s): Xinglong Wang

Last updated by author(s): Nov 22, 2019

Reporting Summary

Nature Research wishes to improve the reproducibility of the work that we publish. This form provides structure for consistency and transparency in reporting. For further information on Nature Research policies, see Authors & Referees and the Editorial Policy Checklist .

Statistics

For all statistical analyses, confirm that the following items are present in the figure legend, table legend, main text, or Methods section.

n/a	Confirmed	
☐	☒	The exact sample size (*n*) for each experimental group/condition, given as a discrete number and unit of measurement
☐	☒	A statement on whether measurements were taken from distinct samples or whether the same sample was measured repeatedly
☐	☒	The statistical test(s) used AND whether they are one- or two-sided<br>*Only common tests should be described solely by name; describe more complex techniques in the Methods section.*
☐	☒	A description of all covariates tested
☐	☒	A description of any assumptions or corrections, such as tests of normality and adjustment for multiple comparisons
☐	☒	A full description of the statistical parameters including central tendency (e.g. means) or other basic estimates (e.g. regression coefficient) AND variation (e.g. standard deviation) or associated estimates of uncertainty (e.g. confidence intervals)
☐	☒	For null hypothesis testing, the test statistic (e.g. *F*, *t*, *r*) with confidence intervals, effect sizes, degrees of freedom and *P* value noted<br>*Give P values as exact values whenever suitable.*
☒	☐	For Bayesian analysis, information on the choice of priors and Markov chain Monte Carlo settings
☒	☐	For hierarchical and complex designs, identification of the appropriate level for tests and full reporting of outcomes
☒	☐	Estimates of effect sizes (e.g. Cohen's *d*, Pearson's *r*), indicating how they were calculated

Our web collection on statistics for biologists contains articles on many of the points above.

Software and code

Policy information about availability of computer code

Data collection	Zen software (Zeiss) and LAS X software (Leica) for image analysis and recording
Data analysis	GraphPad Prism

For manuscripts utilizing custom algorithms or software that are central to the research but not yet described in published literature, software must be made available to editors/reviewers. We strongly encourage code deposition in a community repository (e.g. GitHub). See the Nature Research guidelines for submitting code & software for further information.

Data

Policy information about availability of data

All manuscripts must include a data availability statement. This statement should provide the following information, where applicable:

- Accession codes, unique identifiers, or web links for publicly available datasets
- A list of figures that have associated raw data
- A description of any restrictions on data availability

Case Western Reserve University supports the NIH Guidelines for the Sharing of Research Resources including the “Sharing of Biomedical Research Resources: Principles and Guidelines for Recipients of NIH Grants and Contracts”. If any intellectual property is pursued, the animals or reagents will be shared and distributed following advice from the authorities of Case Western Reserve University.

Field-specific reporting

Please select the one below that is the best fit for your research. If you are not sure, read the appropriate sections before making your selection.

Life sciences study design

All studies must disclose on these points even when the disclosure is negative.

Sample size	The sample size is calculated by power analysis based on a detectable difference of 5% between the groups with an estimated standard deviation using a power of 80% at 0.05/3 significance level for correcting for multiple comparisons. The pilot studies suggest that the difference of 5% between the groups is reachable.
Data exclusions	No sample was excluded in this study.
Replication	The majority of experiments were performed using animals and replicated more than three times with amazingly reproducibility (including analysis of animals or the surgery of animals). These limited in vitro experiments were also replicated more than three times and could be reliably reproduced.
Randomization	No such method was used.
Blinding	All the outcomes were independently assessed by investigator without knowledge of treatments or animal genotypes.

Reporting for specific materials, systems and methods

We require information from authors about some types of materials, experimental systems and methods used in many studies. Here, indicate whether each material, system or method listed is relevant to your study. If you are not sure if a list item applies to your research, read the appropriate section before selecting a response.

Materials & experimental systems

n/a	Involved in the study	
☐	☒	Antibodies
☐	☒	Eukaryotic cell lines
☒	☐	Palaeontology
☐	☒	Animals and other organisms
☒	☐	Human research participants
☒	☐	Clinical data

Methods

n/a	Involved in the study	
☒	☐	ChIP-seq
☒	☐	Flow cytometry
☒	☐	MRI-based neuroimaging

Antibodies

Antibodies used	We provide the detailed information of antibodies in the method section.
Validation	All antibodies used were obtained from commercial sources with clear validation profiles (application, host, source and catalogue number provided). For these antibodies with different lot number, in addition to data sheets provided by the vendors, we will always confirm their authenticity by immunoblot of cells with the knock-down and overexpression of targeted protein or by immunostaining with pre-incubation with antigenic peptides.

Eukaryotic cell lines

Policy information about cell lines

Cell line source(s)	HEK293 cells were obtained from American Type Culture Collection (ATCC).
Authentication	Yes. Cells from ATCC are authenticated by morphology, karyotyping and PCR based approaches according to ATCC.
Mycoplasma contamination	HEK293 cells used in the study were tested free of mycoplasma contamination.
Commonly misidentified lines (See ICLAC register)	HEK293 cells are not listed by ICLAC.

Animals and other organisms

Policy information about studies involving animals; ARRIVE guidelines recommended for reporting animal research

Laboratory animals	All aged NTG and 5XFAD female mice (B6SJL-Tg (APPSwFlLon, PSEN1*M146L*L286V) 6799Vas/Mmjax, stock no. 34840-JAX) used in behavioral tests were born at the same day, directly purchased from the Mutant Mouse Resource & Research Centers (MMRRC) and maintained at Case Western Reserve University.
Wild animals	N/A

Field-collected samples	N/A
Ethics oversight	Mouse surgery and procedures were performed according to NIH guidelines and were approved by the Institutional Animal Care

Note that full information on the approval of the study protocol must also be provided in the manuscript.
